# Programmable multifunctional microrobots for synergistic cuproptosis-immunotherapy

**DOI:** 10.1093/nsr/nwag491

**Published:** 2026-08-13

**Authors:** Chuanhua Li, Jiaxin Li, Xuejia Liu, Xuyang Chen, Shixiao Ding, Yu Zhao, Xiao Luo, Leike Li, Laishou Yang, Shimin Yu, Hao Chang, Fengtong Ji, Tianlong Li, Jie Zhao

**Affiliations:** State Key Laboratory of Robotics and Systems, Harbin Institute of Technology, Harbin 150001, China; Suzhou Research Institute of HIT, Suzhou 215104, China; State Key Laboratory of Robotics and Systems, Harbin Institute of Technology, Harbin 150001, China; Suzhou Research Institute of HIT, Suzhou 215104, China; State Key Laboratory of Robotics and Systems, Harbin Institute of Technology, Harbin 150001, China; State Key Laboratory of Robotics and Systems, Harbin Institute of Technology, Harbin 150001, China; State Key Laboratory of Robotics and Systems, Harbin Institute of Technology, Harbin 150001, China; Key Laboratory of Automotive Power Train and Electronic Control, Hubei University of Automotive Technology, Shiyan 442002, China; State Key Laboratory of Robotics and Systems, Harbin Institute of Technology, Harbin 150001, China; State Key Laboratory of Robotics and Systems, Harbin Institute of Technology, Harbin 150001, China; Department of Oncology Surgery, Harbin Medical University Cancer Hospital, Harbin 150081, China; College of Engineering, Ocean University of China, Qingdao 266100, China; Department of Thoracic Surgery, The First Affiliated Hospital of Harbin Medical University, Harbin 150001, China; Wellcome Trust/Cancer Research UK Gurdon Institute, University of Cambridge, Cambridge CB2 1QN, UK; Department of Physiology, Development and Neuroscience, University of Cambridge, Cambridge CB2 3DY, UK; State Key Laboratory of Robotics and Systems, Harbin Institute of Technology, Harbin 150001, China; Suzhou Research Institute of HIT, Suzhou 215104, China; State Key Laboratory of Robotics and Systems, Harbin Institute of Technology, Harbin 150001, China

**Keywords:** programmable temporal-responsive microrobot (PTRM), magnetic guidance, sequential drug-release program, cuproptosis, immune cell infiltration, immunogenic cell death (ICD)

## Abstract

Oral microrobots as drug carriers have shown great promise in enhancing the therapeutic efficacy of colorectal cancer (CRC). However, existing platforms frequently lack the programmable temporal precision required to execute complex, multistage dosing regimens. Here, we report a programmable temporal-responsive microrobot (PTRM) that autonomously navigates across the gastric acid barrier under external magnetic guidance to execute a sequential drug-release program at orthotopic tumor sites. The PTRM is engineered with a logic-gated architecture, featuring a spatially specific shell for gastric-resistant shielding and exclusive intestinal pH-triggered activation. The system’s internal ‘timer’ is governed by the precise modulation of crosslinking densities within its dual gelatin cores, enabling finely-tuned biphasic release kinetics. Phase 1 executes the programmed release of elesclomol to induce tumor cell cuproptosis, successfully priming the tumor microenvironment by recruiting infiltrating immune cells. Following a precisely tuned 5.5 h interval, Phase 2 triggers the release of PD-1 inhibitors to activate these cells, thereby amplifying immunogenic cell death. This orchestrated sequential cytotoxicity achieved a 76% remission rate in an orthotopic CRC mouse model. Furthermore, the PTRM architecture is highly scalable, accommodating up to six-core configurations for multiphase release—establishing a versatile robotic platform for complex combinatorial therapeutic strategies against recalcitrant cancers.

## Teaser

Oral magnetic multicore microrobots with programmed sequential drug release integrate cuproptosis and immunotherapy to synergistically treat orthotopic colorectal tumors.

## INTRODUCTION

In recent years, the global incidence of colorectal cancer (CRC) has risen significantly, with ∼900 000 annual deaths, ranking second among all cancers in mortality, emerging as a major public health issue that poses a severe threat to human health [1,2]. Currently, surgery, radiotherapy, and chemotherapy are common cancer treatments [3]. These approaches suffer from poor targeting accuracy and severe side effects, which damage patients' normal cells and physical functions [4]. The emergence of immunotherapy has opened up a new avenue for cancer treatment [5–11]. Represented by immune checkpoint inhibitors such as anti-PD-1/PD-L1 drugs, it aims to activate patients' immune systems, driving immune cells to precisely target and attack tumor cells [12]. However, clinical practice has shown that in some patients, the tumor microenvironment (TME) exhibits a state of ‘cold tumor’ with low immune cell infiltration [13]. The number of immune cells within the tumor tissue is scarce [14–17]. This makes the space for stimulating immune activation through single immune inhibitor treatment extremely limited [18–21]. Cuproptosis, a novel cell death modality, has emerged as a focal area in oncology [22–26]. During the process of cuproptosis, cancer cells release a large number of damage-associated molecular patterns (DAMPs), like ‘signal flares,’ which can attract immune cells, such as T cells and macrophages, to infiltrate massively into the tumor site [27,28], facilitating the transformation of the TME from ‘cold’ to ‘hot’ and creating favorable conditions for immunotherapy [26,29]. Based on this, the combination therapy of cuproptosis and PD-1 inhibitor is a highly promising treatment strategy [30,31]. Ideal drug delivery systems, if capable of precise spatial targeting and sequential phased release, first release cuproptosis inducers to remodel the TME into a ‘hot’ tumor state. After sufficient immune cell infiltration, they then release PD-1 inhibitor to activate immune-mediated killing. This not only aligns with the biological progression of combination therapy to maximize synergistic efficacy but also reduces off-target drug exposure to mitigate toxic side effects on normal tissues. However, the existing drug delivery systems fail to achieve the sequential phased drug release, making it difficult to precisely match the complex biological processes of cuproptosis, immune infiltration and immune activation in the body [32–35]. As a result, drugs cannot be administered at the optimal time, greatly limiting the clinical application of this method.

In gastrointestinal (GI) cancer combination therapy, active microrobotic systems have shown unique advantages in tumor targeting, deep tissue penetration, and therapeutic efficacy, as recently highlighted in the literature [36–38]. Notably, biodegradability and hydrolyzability are promising properties in drug delivery. Polymer-based magnetically driven microrobots have been studied for their controllable drug release and gastric acid tolerance [4,39–49]. According to Darcy’s law of diffusion, the porous structure of polymers has a significant impact on the drug release profile, and the diffusion kinetics of drugs in polymers follow the Stokes-Einstein equation [50]. For CRC treatment, the oral administration route is particularly advantageous due to its convenience, high patient compliance, and the ability to deliver drugs locally along the GI tract, thereby minimizing systemic toxicity. However, drug carriers must traverse the harsh gastric acidic environment (pH = 1–3) before reaching intestinal target sites [51,52]. During this process, encapsulated drugs risk degradation and lose efficiency [53]. Hydrogels can leverage their porous networks, superior biocompatibility, and unique swelling-deswelling behavior to enhance encapsulated drug protection [54]. Consequently, they have become leading drug carriers. For CRC intestinal therapy, pH-responsive hydrogels are favored [55–58], because they ensure loaded drugs remain intact through gastric acid and trigger release upon entering the intestinal environment [59]. In CRC, combining multiple drugs synergistically targets multiple pathways such as tumor cell death and immune evasion, effectively overcoming tumor heterogeneity and multidrug resistance (issues that single drugs struggle to address), while achieving enhanced efficacy and reduced toxic responses by reducing the dosage of individual drugs. However, single-stimulus-responsive hydrogels suffer from premature excessive release [60]. In addition, when multiple drugs are combined, their action timelines fail to align with ICD. This not only diminishes efficacy but also risks toxic damage to normal tissues *via* uncontrolled drug release.

To overcome the above challenges, we design programmable temporal-responsive microrobots (PTRMs) with the ability of two-phase drug release in sequence (Fig. 1a). Under the control of a rotating magnetic field (RMF), PTRMs can smoothly pass through the gastric acid barrier, efficiently migrate to the tumor site along the GI tract, and then initiate the drug release program to achieve the sequential and synergistic killing of tumors through cuproptosis and ICD. The PTRMs consist of a calcium alginate shell and two magnetic gelatin cores loaded with elesclomol and PD-1 inhibitor, respectively. The calcium alginate outer membrane plays a crucial protective role, endowing the microrobot with the ability to resist gastric acid erosion and precisely trigger the release of the cores according to the intestinal pH level, effectively preventing premature drug leakage and ensuring the activity of drug molecules. Two gelatin cores are specially designed with different degrees of crosslinking to achieve sequential release of the two drugs (at 0.5 and 6 h, respectively), which matches the immune chemotactic induction time triggered by cuproptosis. The synergistic effect of cuproptosis and ICD brought about by immune chemotaxis activation can kill tumor cells continuously and effectively, providing a new strategy for the treatment of GI malignancies (Fig. 1b). Furthermore, the PTRM architecture is highly scalable, accommodating up to six-core configurations for multiphase release—establishing a versatile robotic platform for complex combinatorial therapeutic strategies against recalcitrant cancers.

**Figure 1.**
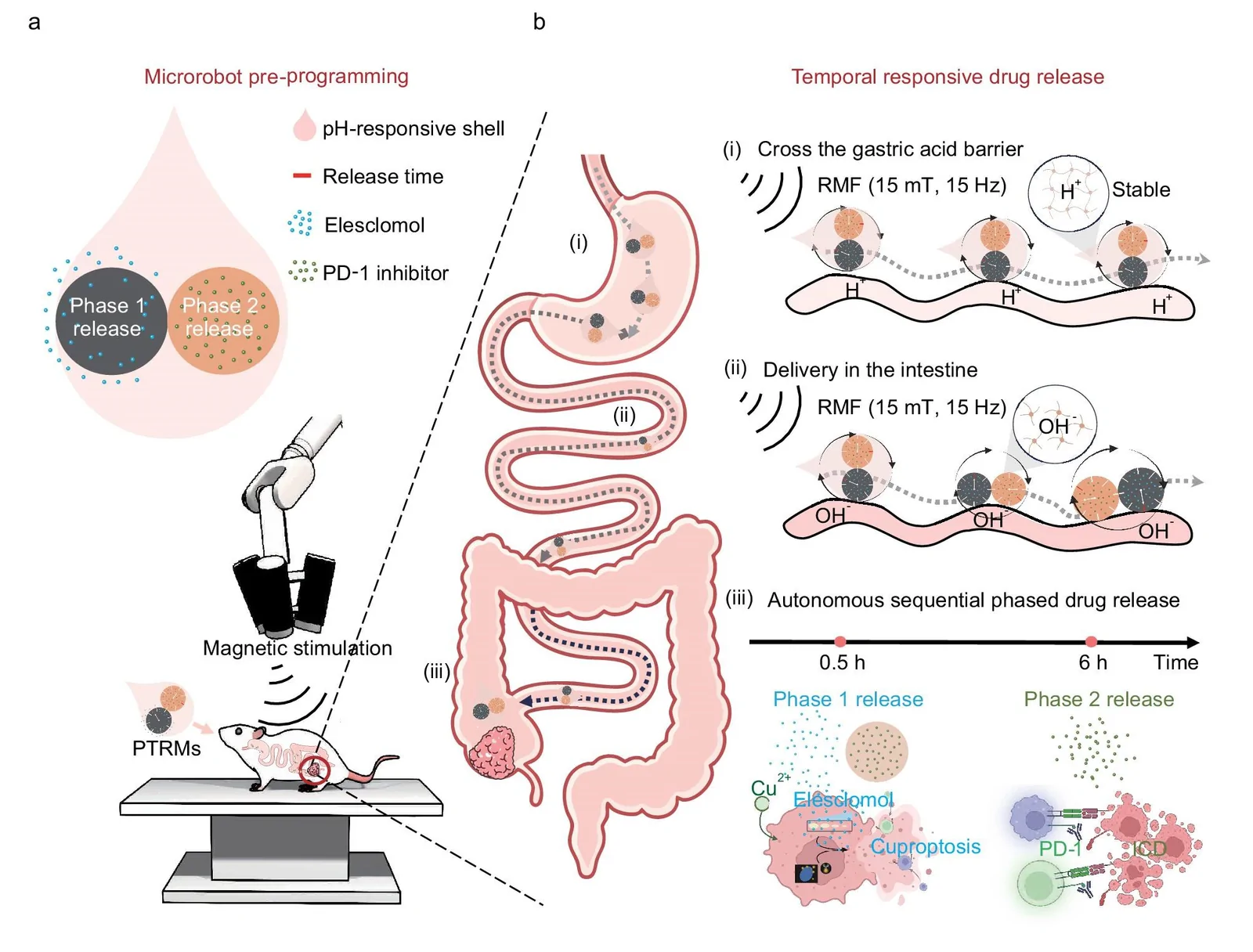
Schematic illustration of PTRMs preparation and the mechanism of temporally responsive drug release for CRC treatment. (a) Scheme illustrating the PTRMs’ structure. The dual-core structure comprises a calcium alginate shell and two gelatin microsphere cores. The two gelatin microsphere cores are a Phase 1 release microsphere loaded with elesclomol (GMS 1) and a Phase 2 release microsphere containing PD-1 inhibitor (GMS 2). (b) Mechanism of autonomous sequential phased drug release with a time interval for CRC treatment. (i) Upon oral administration, PTRMs maintain structural stability in the acidic gastric environment, and are directionally propelled by an external rotating magnetic field (RMF) to advance through the gastric lumen. (ii) Under precise magnetic guidance, PTRMs actively overcome the topographical barriers of intestinal surface folds and the mucus layer to achieve efficient targeted locomotion toward the colonic tumor region, with the magnetic anchoring effect enhancing lesion retention. Upon reaching the colorectal region, the calcium alginate encapsulation disintegrates in the neutral intestinal environment, releasing the integrated dual-sphere core. (iii) Benefiting from *in situ* magnetic anchoring that resists intestinal peristalsis and prevents premature clearance, PTRMs stably reside in the tumor lesion area to execute autonomous sequential phased drug release of elesclomol and PD-1 inhibitor. Elesclomol released in Phase 1 induces tumor cell cuproptosis, accompanied by DAMP release to recruit immune cells. At the 6 h mark, the PD-1 inhibitor is released in Phase 2 to activate recruited immune cells and amplify immunogenic cell death (ICD), and the two effects synergistically exert potent anticancer effects. Created in BioRender (https://BioRender.com/).

## RESULTS

### Fabrication and characterization of PTRMs

To realize autonomous sequential phased drug release and magnetic control, two types of drug-loaded gelatin microspheres were fabricated by leveraging concentrations of gelatin and magnetic particles of varying sizes. Variations in gelatin concentration and magnetic particle content modulated the internal crosslinking degree of gelatin microspheres, thereby leading to differences in their release times at physiological temperatures. A calcium alginate outer membrane was applied to provide acid stability. Figure 2a and Fig. S4 illustrate the process of continuous and integrated preparation of PTRMs. The fabrication process of PTRMs primarily comprised three consecutive steps: (1) extrusion of GMS 1 (5% gelatin with 150 nm Fe_3_O_4_) and GMS 2 (5% gelatin with 20 nm Fe_3_O_4_), (2) extrusion of sodium alginate microspheres enwrapping a double-gelatin core, and (3) cross-linking and solidification *via* Ca^2^⁺ under low-temperature treatment.

**Figure 2.**
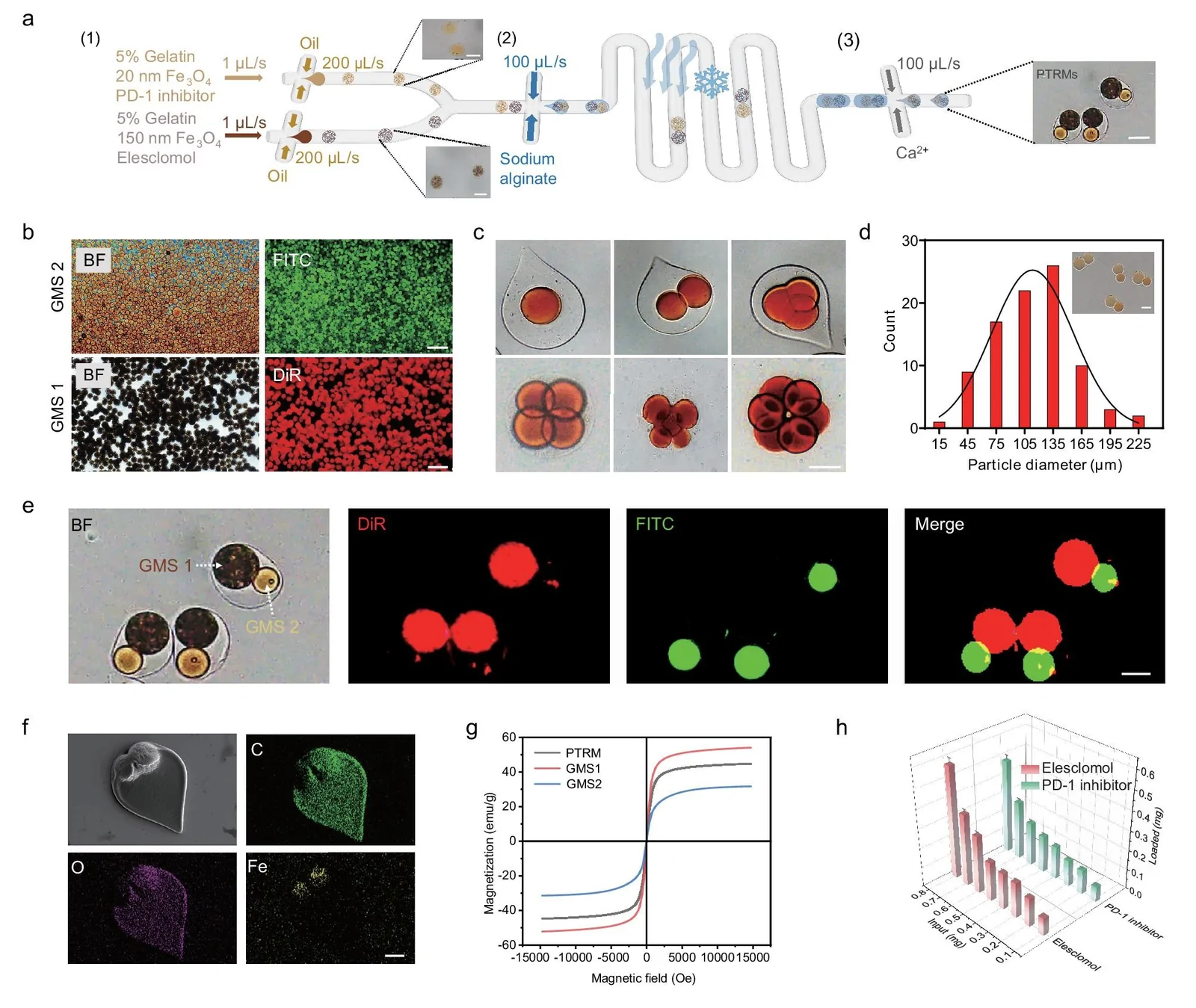
Fabrication and characterization of PTRMs. (a) Schematic showing the integrated preparation method of the PTRMs. Scale bars, 50 μm. (b) Bright-field (BF) and fluorescence images demonstrating the differential loading of Fe_3_O_4_ in the two gelatin cores, as well as the loading of elesclomol (with DiR) and PD-1 inhibitor (with FITC). Scale bars, 200 μm. (c) Image showing the scalable preparation of multi-core microrobots. Scale bar, 50 μm. (d) Size distribution of PTRMs. Scale bar, 50 μm. (e) The bright field and fluorescence images demonstrating the preparation of dual-core microrobots. Scale bar, 50 μm. (f) SEM and EDS images demonstrating the dual-core structure and the distribution of carbon (C), oxygen (O) and iron (Fe) elements within PTRMs. Scale bar, 50 μm. (g) Magnetization (*M*) hysteresis from Superconducting Quantum Interference Device (SQUID) magnetometry of GMS 1, GMS 2 and PTRMs. (h) The drug-loading capacity of elesclomol in GMS 1 and PD-1 inhibitor in GMS 2.

In the first step, we tailored gelatin core properties to tune drug release kinetics, fabricating distinct gelatin microspheres by varying gelatin concentration and the size of loaded Fe_3_O_4_ nanoparticles. The dispersed phase and the continuous phase were introduced into the channels of the microfluidic chip at a flow rate ratio of 200:1, and gelatin microdroplets were obtained *via* shear force. The scanning electron microscope images are shown in Fig. S1. As shown in Fig. 2b, two formulations were produced: GMS 1 and GMS 2. Under bright-field microscopy, GMS 1 exhibited a dark brown hue due to larger Fe_3_O_4_ nanoparticles, whereas GMS 2 appeared pale yellow owing to the incorporation of smaller Fe_3_O_4_ particles (Fig. S3). Under fluorescence microscopy, the microspheres emitted bright red (GMS 1) and green (GMS 2) fluorescence, attributed to efficient encapsulation of 1,1′-Dioctadecyl-3,3,3′,3′-tetramethylindotricarbocyanine perchlorate (DiR)-labeled elesclomol and fluorescein isothiocyanate (FITC)-labeled PD-1 inhibitor, respectively.

Multicore microrobots were fabricated *via* microfluidic technology, wherein preprepared gelatin microspheres were encapsulated within a calcium alginate shell (Fig. 2c). During the fabrication of multicore structures, the particle size of the calcium alginate shell must be precisely matched to the diameter of the inner gelatin cores. The quantitative correlation between the flow rate ratio and the particle size of both gelatin cores and alginate shells was systematically characterized and presented in Fig. S2. Multicore microrobots can be designed with several cores (*n* = 1–6), allowing them to potentially load up to six drugs and achieve preprogrammable release in a desired sequence. In this work, we selected the 3-core microrobot (*n* = 3) to demonstrate sequential multidrug release behavior (Fig. S5), while the 2-core microrobot (*n* = 2) was adopted for combined antitumor therapy validation throughout our study. As a proof of concept, here we demonstrated a two-drug combination—elesclomol and PD-1 inhibitor—by fabricating PTRMs. Particle size distribution analysis (Fig. 2d and Fig. S3) reveals that PTRMs have an average particle size of 120 μm, a size that facilitates navigation within diverse complex luminal environments, including the intestinal tract and blood vessels. Fluorescence microscopy images (Fig. 2e) demonstrate that GMS 1 and GMS 2 are successfully integrated into the interior of the calcium alginate shell. Notably, the length of the water droplet-shaped tail of the shell is significantly shortened at this point, which facilitates its high-speed navigation in the complex GI tract.

Scanning electron microscopy (SEM) images and energy-dispersive X-ray spectroscopy results (Fig. 2f) demonstrated the successful loading of magnetic iron oxide onto the microspheres, thereby enabling PTRMs to respond to the external magnetic field. Hysteresis loops (Fig. 2g and Fig. S6) characterize the magnetic responses of PTRMs and two drug-loaded gelatin microspheres (GMS 1, GMS 2). All three samples exhibit hysteretic behavior, enabling magnetic manipulation *via* external fields. At 15 000 Oe, GMS 1 reaches a saturation magnetization of ∼55 emu/g, GMS 2–32 emu/g, and PTRM ∼45 emu/g—reflecting that PTRM inherits combinatorial magnetic properties from its two encapsulated cores. The magnetic properties enable PTRMs to achieve a 100% response rate under external magnetic fields. Furthermore, high-performance liquid chromatography (Fig. 2h) analyses revealed that the two cores of the solidified PTRMs were loaded with the cuproptosis inducer elesclomol and the PD-1 inhibitor, respectively, with drug encapsulation efficiencies of 60.0% and 50.5%.

### Evaluation of PTRMs' movement in the GI tract *in vitro*

The interior of the GI tract presents an intricate microenvironmental system, characterized by the presence of highly viscous mucus and a surface replete with numerous folds. To explore the motion characteristics under an external magnetic field in the GI environment, we established an *in vitro* experimental system (Fig. S7) to conduct a systematic characterization and analyze the locomotion and manipulation capabilities under diverse viscosity conditions and on the surface of the GI tract. The control and locomotion capabilities are realized in an *in vitro* phosphate-buffered saline (PBS) environment (Figs S8–S11 and Movie S1).

Figure 3a demonstrates the viscosity-dependent locomotion behavior of PTRMs in separately prepared simulated gastric fluid (SGF) and simulated intestinal fluid (SIF), with viscosity gradients precisely tuned via sucrose addition and both fluids calibrated to 5 cP to mimic the postprandial GI environment (full viscosity calibrations in Fig. S12). Quantitative results showed that PTRMs reached maximum velocities of ∼950 μm/s in 1 cP SGF and ∼850 μm/s in 1.2 cP SIF, with the velocity decreasing significantly as viscosity increased in both media due to enhanced viscous drag, while still retaining stable propulsion velocities of ∼550 μm/s and ∼450 μm/s in 5 cP high-viscosity SGF and SIF, respectively (Fig. 3b and c).

**Figure 3.**
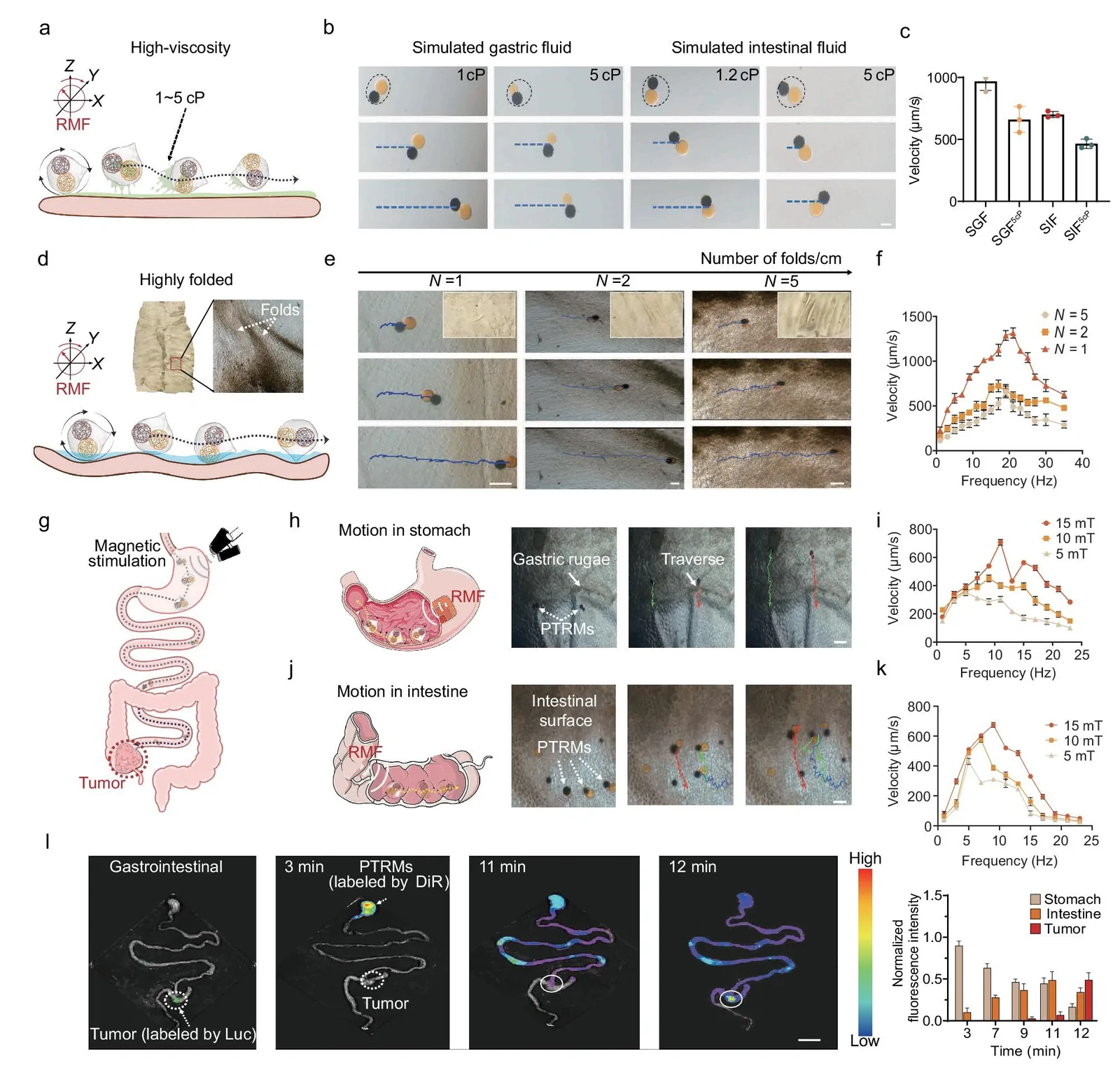
*In vitro* locomotion of PTRMs in the complex GI environment. (a) Schematic illustrating the movement of PTRMs in high-viscosity fluid under RMF-induced resistance. (b) Optical images depicting the movement of PTRMs in simulated gastrointestinal fluids. Scale bar, 50 μm. (c) Velocity of PTRMs under RMF with different viscosities (*n* = 3; means ± SEM). (d) Schematic illustration of PTRMs moving efficiently across manifold surfaces under an RMF. (e) Optical microscopy images show the movement performance of PTRMs on simulated GI surfaces with different folding degrees. Scale bars, 100 μm. (f) The velocity of PTRMs on bioinspired GI surfaces with tunable folding complexity. (g) Schematic illustrating that PTRMs move efficiently in the GI tract for targeting tumors. Created in BioRender. (h) Time-lapse images illustrating the movement of PTRMs in a stomach. Scale bar, 200 μm. (i) Velocity of PTRMs on the stomach surface under the RMF with different magnetic intensities and frequencies (*n* = 3; means ± SEM). (j) Time-lapse images illustrating the movement of PTRMs in the intestine. Scale bar, 100 μm. (k) Velocity–frequency profiles of PTRMs on the intestinal surface under the RMF with different magnetic intensities and frequencies (*n* = 3; means ± SEM). (l) Time-lapse *in vitro* live fluorescence images depicting the targeted movement of PTRMs toward tumor tissues (left) and fluorescence quantification of PTRMs in the stomach, intestine and tumor (right). Scale bar, 1 cm. Luc, luciferase.

To further investigate the locomotion performance of PTRMs within the complex GI environment, we conducted experiments on multifolded surfaces (Fig. 3d). Under varying fold densities (*N* = 1, 2, 5, representing folds per centimeter), PTRMs were actuated by RMFs, and their trajectories were recorded (Fig. 3e). Velocity profiles (Fig. 3f) revealed that across all fold densities, velocity initially increased and then declined with increasing magnetic frequency. Notably, PTRMs demonstrated exceptional adaptability to topological complexities: at low fold density (*N* = 1), they maintained high velocities (over 1300 μm/s) across a broad frequency range; even at the highest density (*N* = 5), where peak velocity decreased to ∼657 μm/s with a narrowed optimal frequency window, PTRMs retained efficient propulsion. These results indicate that while GI folds alter fluid dynamics and spatial constraints, thereby increasing viscous drag and reducing magnetic coupling efficiency, PTRMs exhibit robust locomotion capabilities. This highlights their potential for clinical applications, where tailored magnetic parameters and robotic designs could further optimize their performance on the native GI surface.

To further evaluate the controllable movement of the target object on the complex and multifolded surface of the real GI tract, we surgically dissected and isolated the stomach and intestines of mice. Figure 3g schematically represents that PTRMs are targeted to the tumor site under magnetic-field control. As depicted in Fig. 3h, the magnetically controlled movements of PTRMs on the stomach surface are showcased (Movie S2). It can be observed that two PTRMs successively achieved the traversal of the folds on the stomach surface. Figure 3i presents the velocity-frequency curve of PTRMs responding to the RMF on the stomach surface. Under a magnetic field intensity of 15 mT, the PTRMs attained a high movement velocity of 708 μm/s.

In contrast to the gastric surface, the intestinal surface exhibits a more alkaline environment. The progressive rupture of the calcium alginate outer membrane prompts the dual-core structure to manifest distinct locomotion patterns. As illustrated in Fig. 3j, PTRMs move along the inner intestinal surface. It can be discerned that after the rupture of the calcium alginate outer membrane, due to the adhesion at the contact points of the two gelatin cores during solidification and crosslinking, the dual-core structure still moves as a single unit (Movie S3). Figure 3k displays the velocity-frequency curve of PTRMs responding to the RMF on the intestinal surface. Under a magnetic field intensity of 15 mT, the PTRMs achieved a high movement velocity of 675 μm/s. The degradation of the calcium alginate outer membrane, which reduces its mass, enables the PTRMs to execute more efficient movements within the intestinal environment.

The GI tract presents a convoluted and multibend geometry. In order to evaluate the multiangle variable control and targeting capabilities of PTRMs in the physiologically relevant intestinal environment, we dissected intact GI tracts from mice. First, DiR was used to label the PTRMs, and the *in vitro* simulated flow channel live imaging result verified the successful labeling of the PTRMs (Fig. S13). Subsequently, the PTRMs were introduced into the stomach, enabling them to be driven toward the tumor site (marked with Luc). Eventually, it can be observed from Fig. 3l and Fig. S14 that the DiR-labeled PTRMs demonstrated precise multiangle navigation and smooth targeting within the complex, multibend gastric environment, showing their excellent controllability and tumor-targeting capability.

### Mechanism of the sequential phased drug release and synergistic treatment

In the treatment of CRC, the autonomous sequential phased drug-release process of PTRMs, which enables temporally coordinated and synergistic tumor-killing effect, plays a pivotal role. To elucidate this process, we conducted *in vitro* phased release of drugs, the cuproptosis tumor-killing mechanism, the cuproptosis-mediated immune cell recruitment process, and the ICD process triggered by PD-1 inhibitor-mediated immune suppression reversal.

Figure 4a schematically shows PTRMs’ acid stability, alkali disintegration, autonomous two-phase drug release, and tumor-killing process. The disintegration of the PTRMs' shell in response to the alkaline environment and the autonomous phased release of the two gelatin cores were evidenced in Fig. 4b and Movie S4. The gelatin microsphere disintegration time at 37°C (Fig. 4c) demonstrated that the dual-core sequential phased drug release was achieved by modulating the gelatin concentration and the particle size of the magnetic particles embedded within the gelatin microspheres. Lower gelatin concentrations and larger magnetic nanoparticle sizes led to changes in the looseness of the gelatin internal structure, thereby enabling the controllable release kinetics. We further validated via *in vitro* tumor cytotoxicity assays and orthotopic colorectal carcinoma models established in C57BL mice that the optimal administration interval between Elesclomol and PD-1 inhibitor was 5.5 h (Fig. S15). We used 5% gelatin microspheres loaded with 150 nm magnetic particles and 5% gelatin microspheres loaded with 20 nm magnetic particles as GMS 1 and GMS 2, respectively, to investigate the drug release of elesclomol and PD-1 inhibitor. Figure 4d and Movie S5 show the Phase 1 drug release of elesclomol and the Phase 2 drug release of the PD-1 inhibitor under co-culture conditions with MC38 cells. Furthermore, a 5.5 h time interval between Phase 1 and Phase 2 was validated in Fig. 4e. The cuproptosis process triggered by the Phase 1 elesclomol drug release was schematically depicted in Fig. 4f. Cuproptosis is characterized by DLAT protein oligomerization and degradation of FDX1 and LIAS proteins, prompting us to characterize the expression levels of these three proteins. Figure 4g demonstrates that obvious DLAT aggregation occurred after elesclomol release, and in the western blot results (Fig. 4h), bands appeared at integer multiples of 70 kDa (DLAT), namely 140 kDa and 200–220 kDa, further indicating the aggregation of DLAT protein. Figure 4i shows the degradation of FDX1 and LIAS, suggesting that cuproptosis disrupted mitochondrial-related functions, which is a cause of cell death. Additionally, the cuproptosis process generated high levels of reactive oxygen species (ROS) that killed tumor cells (Fig. 4j). This was because when divalent copper ions were reduced to monovalent copper ions by FDX1, Cu^2+^ was re-oxidized and transferred electrons to molecular oxygen, generating superoxide anions.

**Figure 4.**
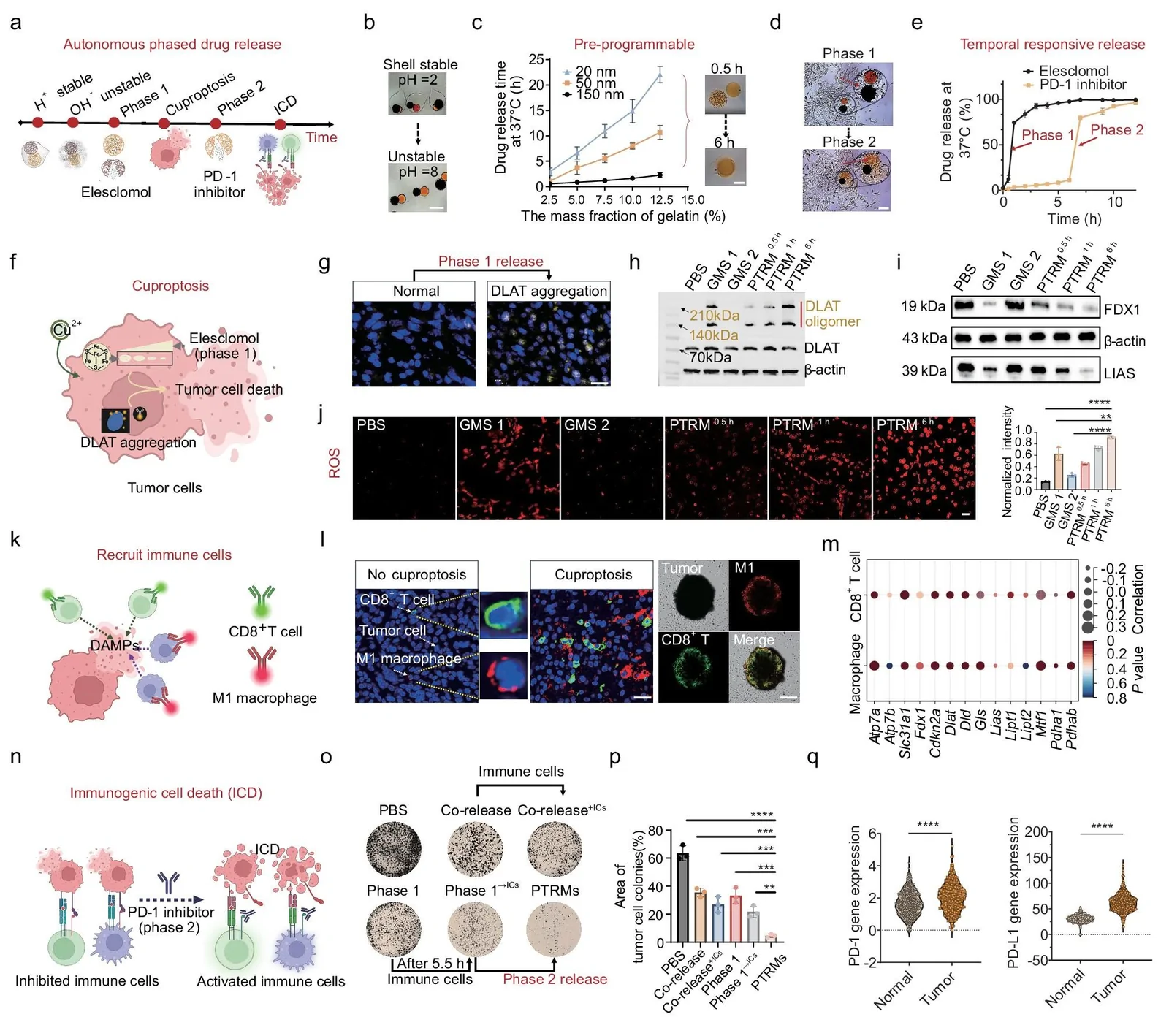
Mechanism of the sequential phased drug release and synergistic treatment. (a) Schematic of the mechanism of sequential phased drug release for tumor cell killing. Created in BioRender. (b) Bright-field microscopic images demonstrating that the calcium alginate shells remain intact in a gastric acid liquid environment (pH ∼ 2) and rupture in an intestinal environment (pH ∼ 8). Scale bar, 100 μm. (c) The sequential rupture of the two gelatin microspheres at an interval time at 37°C, and this time interval is determined by the gelatin concentration and the particle size of the encapsulated Fe_3_O_4_. Scale bar, 50 μm. (d) Bright-field microscopic images demonstrating the Phase 1 drug release of elesclomol and the Phase 2 drug release of the PD-1 inhibitor under co-culture conditions with MC38 cells. Scale bar, 100 μm. (e) The drug release kinetics of GMS 1 loaded with elesclomol and GMS 2 loaded with the PD-1 inhibitor. (f) Schematic illustration of the process during which cuproptosis occurs. Created in BioRender. (g) Confocal Laser Scanning Microscope (CLSM) images of DLAT protein before (left) and after (right) cuproptosis. Scale bar, 20 μm. (h) Western blot assay of DLAT expression in MC38 cells. Six groups: PBS, Phase-1-release gelatin microsphere with elesclomol (GMS 1) after 6 h incubation, Phase-2-release gelatin microsphere with PD-1 inhibitor (GMS 2) after 6 h incubation, and PTRMs after 0.5 h, 1 h, and 6 h incubation, respectively. (i) Western blot assay of expression of iron-sulfur protein clusters (FDX1 and LIAS) in MC38 cells. Groups are the same as (h). (j) CLSM images of MC38 cells staining with DHE (indicating elesclomol-induced ROS generation). Scale bar, 40 μm. (k) Schematic illustration of the promotion of immune cell recruitment by cuproptosis. Created in BioRender. (l) CLSM images of tumor spheres in the cuproptosis or non-cuproptosis following a 6 h co-culture period with macrophages (red) and T cells (green). Scale bar (left), 20 μm. Scale bar (right), 200 μm. (m) Correlation analysis between cuproptosis-related genes and immune cell infiltration. (n) Schematic illustration of the process during which ICD occurs due to the release of PD-1 inhibitor by the delayed-release gelatin microsphere with PD-1 inhibitor (GMS 2). Created in BioRender. (o) Assessment of cytotoxic effects by clonogenic assay. MC38 tumor cell colonies were subjected to six distinct treatment regimens: PBS group, Co-release of Elesclomol and PD-1 inhibitor (Co-release group), Co-release with concurrent immune cells (ICs) (Co-release^+ICs^ group), PTRMs executing Phase 1 release (Phase 1 group), Phase 1 release followed by ICs supplementation after 5.5 h (Phase 1^→ICs^ group), and the complete PTRMs sequence (colonies were treated with PTRMs for 5.5 h to allow for Phase 1 drug release. The medium was then replaced to remove the supernatant and supplemented with ICs, immediately followed by the activation of the Phase 2 release) (PTRMs group). Twelve hours after the final treatment, the plates were stained with crystal violet to quantify the surviving colonies. (*n* = 3; means ± SEM). (p) Quantification of tumor cell colonies’ area in various groups. (*n* = 3; means ± SEM). (q) Analysis of PD-1 and PD-L1 gene expression differences between normal and tumor tissues based on clinical transcriptome data. Statistical significance was determined by one-way ANOVA followed by Tukey’s post hoc test for (j), (o) and (p), and by Student’s *t*-test for (q). **P < 0.01; ***P < 0.001; ****P < 0.0001.

During cuproptosis, a series of DAMPs were released, which could act as signaling molecules to recruit immune cells. Figure 4k, l and Fig. S16 illustrate the cuproptosis-mediated immune cell infiltration process and provide cellular evidence for the subsequent initiation of immunogenic cell death (ICD), with substantial infiltration of CD8⁺ T cells (green), M1 macrophages (red), neutrophils (pink) and natural killer (NK) cells (yellow) detected in *in vitro*-cultured tumor cells at 6 h after cuproptosis induction. In stark contrast, cuproptosis-insensitive FDX1-knockdown tumor cells failed to trigger robust infiltration of CD8⁺ T cells and M1 macrophages under identical treatment conditions. This loss-of-function phenotype inversely confirms that immune cell recruitment is strictly dependent on tumor cell cuproptosis and downstream DAMP release (Fig. S17). In addition, cuproptosis also drives the recruitment of Tregs and dendritic cells (DCs). The absolute number of infiltrated Tregs remains relatively low, whereas abundant DCs accumulate within the TME. More importantly, the recruited DCs can be further activated upon exposure to the sequentially released PD-1 inhibitor (Fig. S18). Correlation analysis between cuproptosis-related genes and immune cell infiltration based on clinical transcriptome data of human CRC (Fig. 4m and Figs S19–S21) revealed that 8 out of 14 cuproptosis-associated genes exhibited significant associations with immune infiltration. This finding further validates the generalizability of cuproptosis-mediated immune cell infiltration to humans. After cuproptosis-mediated immune cell infiltration occurred, the infiltrated immune cells were in an immunosuppressed state due to the influence of PD-L1 on the tumor cell surface. Consistent with clinical transcriptomic data, immunofluorescence staining verified abundant PD-1 expression in colorectal cancer tissues (Fig. S22a), indicating widespread immune suppression in the TME. Elesclomol-induced cuproptosis successfully recruited CD86⁺ M1 macrophages and CD8⁺ cytotoxic T cells into tumor lesions (Fig. S22b). Further experiments demonstrated that subsequent treatment with PD-1 inhibitor relieved PD-1-mediated immunosuppression, restored the activation of infiltrated immune cells and markedly inhibited tumor growth (Fig. S22c and d). Figure 4n illustrates the process by which PD-1 inhibitor blocked the binding of PD-1 to reverse immunosuppression and induce ICD. The clonogenic assay revealed that the potent cytotoxic effect, mediated by ICD downstream of cuproptosis-driven immune infiltration, was critically dependent on the orchestrated, sequential phased drug release enabled by the PTRMs platform. Against MC38 tumor cell colonies subjected to six treatment regimens—PBS, Co-release, Co-release^+ICs^, Phase 1 only, Phase 1^→ICs^, and the full PTRMs sequence—only the complete PTRMs protocol achieved near-complete eradication of colonies (Fig. 4o and p). This stark contrast, quantified 12 h post-treatment, definitively shows that cuproptosis-induced immune priming must be followed by checkpoint blockade to unleash effective ICD and abolish clonogenic survival. The immune difference analysis of PD-1 and PD-L1 in normal and tumor tissues based on human CRC clinical transcriptome data indicated that PD-1 and PD-L1 were highly expressed in tumor tissues (Fig. 4q), representing a highly immunosuppressive environment, demonstrating the clinical effectiveness of the Phase 2 drug release of PD-1 inhibitor.

### Active drug delivery to CRC *in vivo*

Subsequent to the characterization of the *in vitro* motility and autonomous sequential phased drug release of the PTRMs, we proceeded to explore their active targeting and delivery to colorectal tumors *in vivo*. To achieve this, orthotopic and metastatic CRC models were constructed *via* cecal orthotopic injection of luciferase-expressing MC38 cells. Subsequently, all tumor-bearing mice were categorized into a magnetically controlled group and a non-magnetically controlled group. To visualize the distribution of PTRMs within the mice, we used an *in vivo* fluorescence imaging system (IVIS). DiR was incorporated into the PTRMs prior to oral administration. As depicted in Fig. 5a, following oral ingestion, the PTRMs were directed toward the cecal tumor under the influence of an RMF.

**Figure 5.**
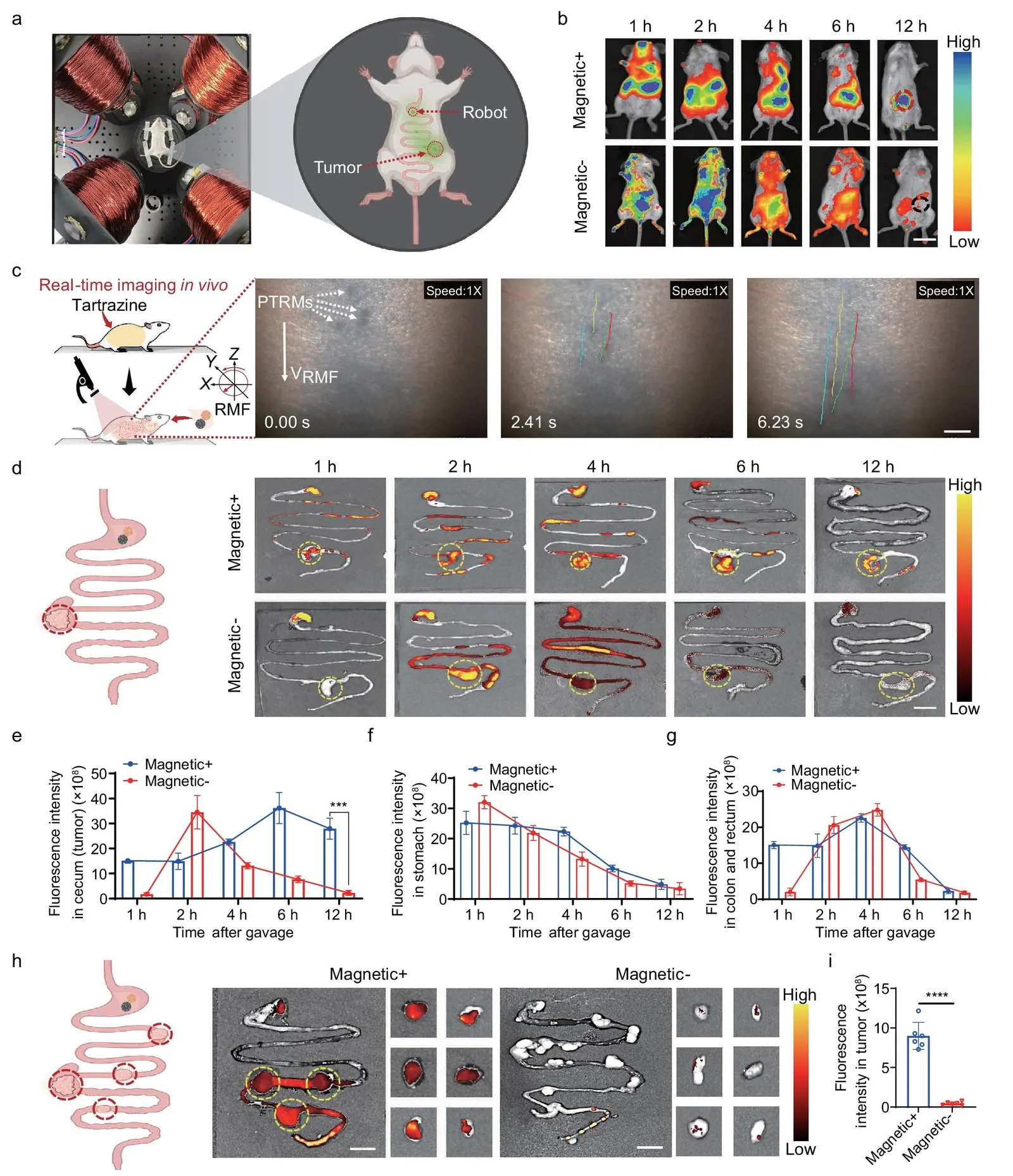
Characterization of oral PTRMs for *in vivo* targeted drug delivery. (a) Active accumulation of PTRMs (labeled with DiR) to CRC (where the luciferase protein is utilized for bioluminescence) through magnetic propulsion. Created in BioRender. (b) After oral administration of PTRMs, *in vivo* fluorescence images of non-magnetically controlled CRC-bearing mice (magnetic-group), and CRC-bearing mice treated with magnetic control (magnetic + group) for 1 h, 2 h, 4 h, 6 h, and 12 h. Scale bar, 3 cm. (c) Stereomicroscopic time-lapse images of PTRMs movement under an RMF in the intestinal tract of *in vivo* tartrazine-cleared mice. Scale bar, 400 μm. (d) Fluorescence images showing the distribution of PTRMs within the GI tract of CRC-bearing mice after oral administration for 1 h, 2 h, 4 h, 6 h, and 12 h (magnetic- group) and magnetic control following oral administration for 1 h, 2 h, 4 h, 6 h, and 12 h (magnetic + group). Scale bar, 1 cm. (e–g) Quantitative analysis of the fluorescence intensity at the cecum (tumor sites), stomach and colorectum. (h) Fluorescence images showing the distribution of PTRMs within the GI tract of mice bearing metastatic CRC after oral administration (magnetic- group) and magnetic control following oral administration (magnetic + group). Scale bars, 1 cm. (i) Quantitative analysis of the fluorescence intensity at the metastatic CRC tumors. Statistical significance was determined by Student’s *t*-test for (e) and (i). ***P < 0.001; ****P < 0.0001.

We first conducted experiments in an orthotopic CRC model. As the PTRMs were labeled with DiR, they emit near-infrared fluorescence in the 800 nm wavelength band upon excitation at 743 nm. Thus, an IVIS was utilized to visualize and monitor their distribution within the body. The *in vivo* fluorescence imaging outcomes (Fig. 5b and Fig. S23) illustrated the distribution of PTRMs in both the magnetically controlled (magnetic+) and non-magnetically controlled groups (magnetic-) at 1, 2, 4, 6, and 12 h intervals post-oral administration. To enable real-time observation of PTRMs’ motion under an external magnetic field, anesthetized live Balb/c mice underwent abdominal clearing with tartrazine, allowing direct visualization of PTRMs within the intestinal tracts via a stereomicroscope (Fig. 5c and Movie S6). Quantitative analysis of these *in vivo* recordings revealed that the average locomotion velocity of PTRMs in live intestinal tracts was significantly lower than in *in vitro* SIF, owing to the additional hydrodynamic drag and mechanical obstruction imposed by native mucosal folds, heterogeneous mucus layers and intestinal peristalsis (Fig. S24). In the magnetic-group, the fluorescence intensity was diffusely distributed across the abdominal region with pronounced signals during the initial 1 h and 2 h periods. However, at 4 h, 6 h, and 12 h time points, a conspicuous attenuation in the abdominal fluorescence intensity, especially at the tumor inoculation site, was observed. This attenuation was attributed to the expulsion of PTRMs from the body as a consequence of the murine GI peristalsis. In the magnetic+ group, under magnetic guidance, the PTRMs achieved early cecal tumor targeting (within 1 h) amid initial GI distribution, with pronounced enrichment at 4 h and durable signal retention at the tumor site for over 12 h. This finding implies that the PTRMs, in response to the RMF, can overcome the intricate GI environment *in vivo* and be precisely targeted to the cecal tumor locus. To further scrutinize the distribution of PTRMs within the GI tract, the GI tracts of mice subjected to RMF control for 1 h, 2 h, 4 h, 6 h, and 12 h were surgically retrieved and imaged using *in vivo* fluorescence imaging (Fig. 5d). The quantified fluorescence intensity results at the stomach, colon, rectum, and cecal (tumor) are presented in Fig. 5e–g. Remarkably, at 12 h time points, the tumor fluorescence signal intensity in the magnetically controlled group was 12.5-fold greater than that in the non-magnetically controlled group, thereby validating the efficacious targeting and delivery of PTRMs to the tumor site under RMF.

Then, to ascertain the capacity of PTRMs to effectuate drug delivery to multiple lesion sites, experiments were conducted on mice with metastatic CRC. In contrast to the orthotopic CRC experiment, owing to the necessity for multisite targeted delivery, the oral PTRMs were administered in aliquots, and targeted manipulation of PTRMs was executed for diverse tumor lesion sites. As illustrated in Fig. 5h, in magnetic-group, no appreciable fluorescence signals were detected at multiple metastatic foci. Conversely, in magnetic+ group, conspicuous fluorescence signal distributions were observable at the cecal tumor and two metastatic tumors within the colon. The results of fluorescence quantitative analysis are presented in Fig. 5i, indicating a substantially elevated fluorescence signal in the magnetically controlled group compared with magnetic-group. This demonstrates that we can accomplish drug delivery to multiple lesion sites through multiple oral administrations and sequential targeted manipulation. Ultimately, the PTRMs achieve autonomous and sequential phased drug release at the lesion site (Fig. S25 and Movie S7), laying the foundation for subsequent synergistic therapy.

### Evaluation of the anticancer therapeutic efficacy of PTRMs *in vivo*

The therapeutic efficacy of PTRMs was assessed *in vivo* through a murine orthotopic colorectal tumor model established by the inoculation of luciferase-expressing MC38 into mice over 5 days (Fig. 6a). Additionally, we constructed an additional orthotopic colorectal tumor model using CT26 cells to conduct multimodel verification of therapeutic effects (Fig. S26). Tumor-bearing mice received treatment of either PBS, oral administration of PD-1 inhibitor and elesclomol (PD1^i^ + elesclomol^p.o.^), caudal vein injection of PD-1 inhibitor and elesclomol (PD1^i^ + elesclomol^I.V.^), passive PTRMs (PTRM^magnetic−^) or magnetically guided PTRMs (PTRM^magnetic+^) at 5-day intervals over three sessions. Tumor progression was monitored *via* the bioluminescence signal emitted by MC38 cells throughout the treatment period (Fig. 6b and c). By day 30, a ∼92% decrease in bioluminescence was observed in the PTRM^magnetic+^ group, indicating a large reduction in tumor sizes. In contrast, PD1^i^ + elesclomol^p.o.^, PD1^i^ + elesclomol^I.V.^ and PTRM^magnetic−^ groups showed a less complete reduction in bioluminescence relative to PBS-treated controls. To verify the unique role of programmed sequential drug release, we additionally established two magnetically controlled co-release groups (Magnetic GMSA&B^co-release^ and Magnetic PTRM^co-release^). With identical drug dosages and magnetic guidance conditions, these two groups exerted markedly inferior tumor inhibition effects compared with the PTRM^magnetic+^ group, demonstrating that the temporally programmed release strategy greatly enhances the synergistic therapeutic effect of the two drugs (Fig. S27).

**Figure 6.**
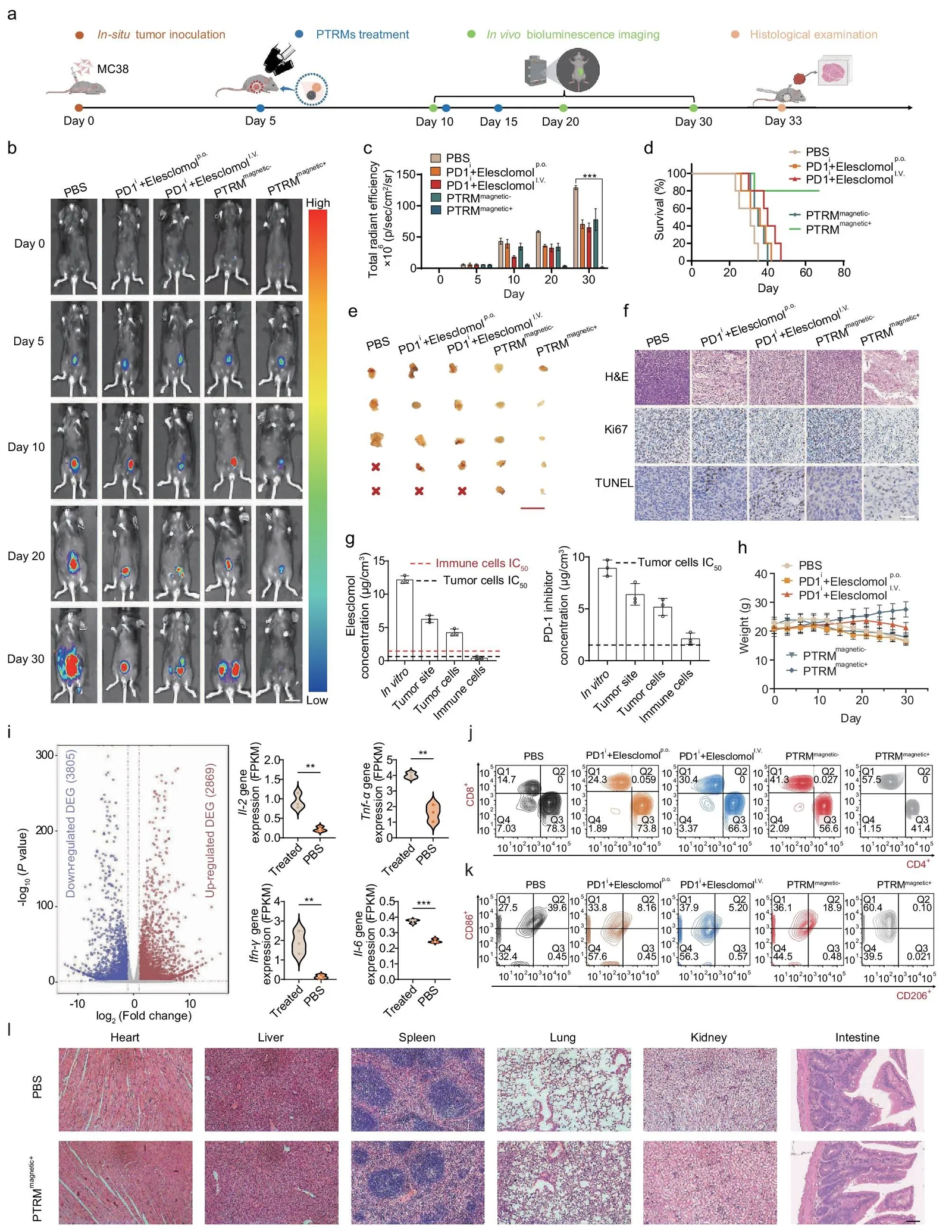
The PTRMs effectively alleviate CRC. (a) Schematic of the study protocol in establishing orthotopic MC38 colorectal tumors in C57BL/6 mice, followed by treatment and tumor section detection. Created in BioRender. (b and c) The utilization of *in vivo* bioluminescence imaging to monitor the growth and metastases of MC38 CRC after different treatment regimens. Error bar represents mean ± SD (*n* = 3). Representative *in vivo* imaging system (IVIS) images of the luciferase-expressing MC38 colorectal tumor-bearing mice (b) and quantified relative bioluminescence intensity (c) upon various administrations over the treatment course, including PBS, PD1^i^ + elesclomol^p.o.^, PD1^i^ + elesclomol^I.V.^, PTRM^magnetic−^ and PTRM^magnetic+^ on Days 5, 10 and 15. Error bars in (c) represent the SD from three mice. Scale bar, 3 cm. (d) Survivorship curves of MC38 tumor-bearing mice after the treatments. (e) Photomicrographs of colorectal tumor tissues from different groups of mice. Scale bar, 1 cm. (f) Hematoxylin and eosin (H&E), Ki67 and TUNEL staining of colorectal tumor cross sections from MC38 tumor-bearing mice of different groups at the end of the study. Scale bar, 100 μm. (g) Concentrations and half-maximal inhibitory concentration (IC₅₀) values of elesclomol and PD-1 inhibitor *in vitro*, at tumor sites, in tumor cells, and in immune cells following PTRM administration. (h) Body weights of mice in different groups during the whole treatment process. (i) Volcano plots displayed the differentially expressed genes of the PTRM^magnetic+^ group (Treated group) compared to the PBS group (left). Gene expression levels of cytokine-related genes in PTRM^magnetic+^ group (Treated group) and PBS group (right). (j) FACS plots and statistical data of CD4^+^ T cells and CD8^+^ T cells induced by the above-mentioned treatments on mice. (k) FACS plots and statistical data of M1 cells (CD86^+^) and M2 (CD206^+^) cells induced by the above-mentioned treatments on mice. (l) Histological evaluation of biosafety. Representative H&E-stained sections of major organs (heart, liver, spleen, lung, kidney, intestine) from mice treated with PTRM^magnetic+^ or the PBS group are shown. Scale bar, 250 μm. Statistical significance was determined by one-way ANOVA followed by Tukey’s post hoc test for (c), and by Student’s *t*-test for (i). **P < 0.01; ***P < 0.001.

Furthermore, the therapeutic efficacy of PTRMs on colorectal tumors was evaluated by recording the survival time of tumor-bearing mice (Fig. 6d). In the phosphate-buffered saline (PBS) control group, all mice died on Day 35 after tumor inoculation. Notably, although tumor suppression was observed in PD1^i^ + elesclomol^p.o.^ group in prior *in vivo* fluorescence imaging, the median survival time of mice in this group showed no significant difference compared with that of the PBS control group. This was mainly attributed to the fact that it was difficult for the PD-1 inhibitor to penetrate the gastric acid barrier when administered orally, leading to its inactivation. While elesclomol alone had a certain tumor-killing ability, it failed to induce a long-term immune response, resulting in rapid tumor recurrence after the drug action ended and threatening the survival of mice. In PTRM^magnetic−^ group, PTRMs were ineffective due to a lack of magnetically guided targeting. The median survival time of mice in the PD1^i^ + elesclomol^I.V.^ group was prolonged to a certain extent. However, the nonsequential drug action prevented the achievement of optimal efficacy. Notably, in the PTRM^magnetic+^ group, the PTRMs were precisely targeted to the tumor site through a magnetic field, achieving autonomous sequential phased drug release. With the synergistic killing effect of cuproptosis and ICD, the median survival time of mice was significantly prolonged. On Day 30, the tumor tissues of surviving mice were dissected and photographed, and the results showed that the tumor mass in the PTRM^magnetic+^ treatment group was significantly smaller than that in other groups (Fig. 6e). At the histological level, compared with other experimental groups, obvious tumor tissue damage was observed in the PTRM^magnetic+^ group, while the cell proliferation marker Ki67 was lowly expressed and the apoptosis-related marker TUNEL was highly expressed (Fig. 6f). These phenomena indicate that the proliferation ability of tumor cells in this group was significantly inhibited, and the apoptosis process was effectively activated, further confirming the excellent therapeutic effect and unique advantages of PTRM^magnetic+^ in the treatment of CRC. Quantitative drug analysis of the cecum (tumor site), sorted tumor cells and intratumoral immune cells from mice treated with magnetic PTRMs further confirmed that the intratumoral elesclomol concentration was sufficient to trigger cuproptosis in tumor cells, yet fell below the threshold required to induce cuproptosis in intratumoral immune cells. Beyond the inherent difference in cuproptosis sensitivity between tumor and immune cells, the programmed sequential drug release feature of dual-core PTRMs serves as an additional protective barrier, ensuring that immune cells recruited to the tumor site at later stages are barely exposed to high-dose elesclomol. For the PD-1 inhibitor, its concentration at the tumor site was well above its half-maximal inhibitory concentration (IC₅₀), which is sufficient to effectively activate immune cells and further drive ICD (Fig. 6g and Fig. S28). No significant changes in body weight were observed in mice across all groups throughout the experiment (Fig. 6h).

To further define the *in vivo* mechanism underlying PTRM-mediated sequential therapy, tumors collected after treatment were analyzed by immunostaining, flow cytometry, and transcriptomics (Fig. S29). Immunofluorescence staining of tumor sections showed pronounced DLAT aggregation accompanied by ROS accumulation in the PTRM^magnetic+^ group, whereas only weak signals were detected after free-drug administration or passive PTRM treatment (Figs S30–S32). These results indicate that magnetic enrichment of PTRMs at the tumor site efficiently initiated cuproptosis *in vivo*.

Transcriptomic profiling further supported this mechanism. Compared with PBS treatment, PTRM^magnetic+^ induced broad transcriptional reprogramming, with 3805 genes up-regulated and 2869 genes down-regulated (Fig. S33). Cuproptosis-related genes, including CDKN2A, FDX1, and LIPT2, were markedly increased, whereas DLD, GLS, LIAS, MTF1, and SLC31A1 were reduced (Fig. S34), consistent with disrupted copper homeostasis, mitochondrial metabolism, and redox balance. In parallel, genes encoding immune effector cytokines, including IL-2, IFN-γ, TNF-α, and IL-6, were strongly induced after PTRM^magnetic+^ treatment (Fig. 6i). KEGG and GSEA analyses revealed enrichment of IL-2–STAT5, IL-6–JAK–STAT3, IFN-γ response, and TNF-α–NF-κB signaling pathways (Figs S35–S37), indicating activation of cytokine-driven antitumor immunity.

Consistent with these transcriptional changes, flow cytometry confirmed substantial immune remodeling within the TME. PTRM^magnetic+^ increased the proportion of intratumoral CD8^+^ T cells to 57.5%, compared with 14.7% in the PBS group, 24.3% in the PD1^i^ + elesclomol^p.o.^ group, 30.4% in the PD1^i^ + elesclomol^I.V.^ group, and 41.3% in the PTRM^magnetic−^ group (Fig. 6j and Fig. S38). Similarly, the fraction of M1-like CD86^+^ macrophages reached 60.4% after PTRM^magnetic+^ treatment, exceeding that in the PBS, PD1^i^ + elesclomol^p.o.^, PD1^i^ + elesclomol^I.V.^, and PTRM^magnetic−^ groups (Fig. 6k and Fig. S39). Together, these data show that magnetically guided PTRMs first induce cuproptosis-associated tumor damage and then promote PD-1 blockade–mediated immune activation, thereby coupling ICD with a remodeled antitumor microenvironment.

In addition to the therapeutic efficacy, the biocompatibility of PTRMs during the *in vivo* treatment was evaluated. Histological examination of major organs including the intestine, heart, liver, spleen, lung and kidney revealed no obvious pathological changes relative to the PBS group (Fig. 6l).

## DISCUSSION

The application of oral microrobot systems in CRC treatment confronts three core challenges: (1) drugs are susceptible to premature leakage or inactivation when traversing the gastric acid barrier, requiring preservation of their tumor-killing activity until reaching the intestinal tract; (2) the complex GI environment characterized by high viscosity and multiple folds leads to difficulty in achieving efficient targeted navigation and control of microrobots; (3) the release timing is difficult to synchronize with tumor-killing biological processes (e.g., immune responses, cell death mechanisms), resulting in suboptimal combination therapy efficacy, in particular for multiple-drugs-in-one treatment.

To overcome these three challenges, this study proposes a temporally responsive microrobot-based strategy, enabling the robot to navigate through complex spaces and execute sequential phased drug release in accordance with a predefined program. For the issue of drug leakage and inactivation caused by the gastric acid barrier, an acid-stable calcium alginate shell is used to encapsulate the microrobot core structure. This shell maintains structural integrity in the strongly acidic gastric environment, preventing premature drug exposure to gastric acid, and ruptures in response to pH changes in the alkaline intestinal environment to release drugs, achieving protection of drugs during their transit through the gastric acid barrier. For the challenge of efficient targeted movement in complex GI environments, the microrobot core undergoes droplet-shaped morphological parameter design and asymmetric structure construction (a dual-core structure prepared using different gelatin concentrations and magnetic particle sizes). The droplet-shaped streamlined design reduces mucus resistance, while the asymmetric core generates a unique rotational propulsion mode under magnetic field action. Combined with dual-core cross-linking technology to ensure structural integrity after shell rupture, this enables efficient motion control on highly viscous and folded GI surfaces. For the problem of weak synergistic drug action and mismatched timing, precise regulation of gelatin microsphere cross-linking degree achieves autonomous sequential phased drug release. The cuproptosis inducer (elesclomol) and PD-1 inhibitor are loaded into gelatin microsphere cores with different cross-linking degrees. By adjusting gelatin concentration and magnetic particle size, the low-cross-linking core preferentially releases elesclomol to induce cuproptosis and initiate immune cell chemotaxis, while the high-cross-linking core delays PD-1 inhibitor release to match the timing of ICD, achieving a ‘kill-first, immunize-second’ synergistic effect which maximizes the overall therapy efficacy.

Compared with conventional injection or oral drug delivery systems, our PTRM platform enables active magnetic navigation and spatiotemporally controlled drug release that cannot be achieved by traditional passive systems. The efficient targeted navigation and control performance of PTRMs achieves a tumor lesion drug concentration of 43.2 μg/g, far higher than that of traditional drug delivery systems (≤20.0 μg/g). Owing to the highly synergistic killing effect of cuproptosis and ICD mediated by elesclomol-induced immune cell infiltration and subsequent PD-1 inhibitor–mediated immunosuppression reversal, the tumor remission rate reaches 76%, significantly higher than that of traditional drug delivery systems (≤58%).

In clinical practice, the real-time imaging of PTRMs may still face certain challenges. In this work, the IVIS has provided one demonstration for the active target delivery of dual-responsive PTRMs in the mouse GI tract. The system tracks the dynamic positions of PTRMs, but due to low spatial resolution and low tissue penetration depth, it is unable to identify individual PTRM or determine the number of PTRMs in human tissues. A more advanced real-time, multiscale *in vivo* imaging system needs further investigation for the PTRM system. Moreover, with the expansion of the cluster size, the emergence and navigation of PTRM clusters exposed to RMF are expected to be directly visualized *in vivo*, which can be detected using current imaging techniques, providing a possible solution to the real-time imaging problem.

## MATERIALS AND METHODS

### Preparation and characterization of PTRMs

PTRMs were synthesized *via* a dual-tube microfluidic channel architecture. 1 mL of 5% A gelatin, pre-incubated at 40°C, incorporating 250 mg of 150 nm Fe_3_O_4_ nanoparticles and 6 mg of DiR-tagged elesclomol, and 1 mL of 5% B gelatin, preincubated at the same temperature, containing 250 mg of 20 nm Fe_3_O_4_ nanoparticles and 6 mg of FITC-conjugated PD-1 inhibitor, were employed in the dispersed phase. Natural vegetable oil supplemented with 2% Tween 20 functioned in the continuous phase. GMS 1 and GMS 2 were extruded through two parallel-arrayed cross-microfluidic channels, respectively. Upon convergence, the combined GMS 1 and GMS 2 were dispersed within a 5% sodium alginate aqueous solution (serving as the dispersed phase), with natural vegetable oil containing 2% Tween 20 remaining as the continuous phase. Dual-core micro-droplets were extruded through a cross-microchannel and then cross-linked and solidified using 5% CaCl_2_ to generate PTRMs. A fluorescence-inverted microscope (Olympus) was utilized to capture both bright-field and fluorescence images. SEM images and energy-dispersive X-ray (EDX) spectra were acquired using a HITACHI SU3800 SEM. The drug-loading capacities of elesclomol and PD-1 inhibitor were determined by high-performance liquid chromatography (HPLC) and enzyme-linked immunosorbent assay (ELISA), respectively. Particle size analysis was performed using ImageJ software.

Detailed methods are provided in the online supplementary material.

## Supplementary Material

nwag491_Supplemental_Files
