# Supplementary material for: Programmable multifunctional microrobots for synergistic cuproptosis-immunotherapy
Source: Natl Sci Rev. 2026 Aug 13;13(20):nwag491. doi: 10.1093/nsr/nwag491 (PMC13750163; doi:10.1093/nsr/nwag491)
Supplement: Supplementary file 1 [file nwag491_supplemental_files.zip › Supplementary materials-NSR-Revised-Clean.pdf]

Supplementary Materials for

Programmable multifunctional microrobots for synergistic cuproptosis-immunotherapy

Chuanhua Li, Jiaxin Li, Xuejia Liu, Xuyang Chen, Shixiao Ding, Yu Zhao, Xiao Luo, Leike Li, Laishou Yang, Shimin Yu, Hao Chang, Fengtong Ji, and Tianlong Li, Jie Zhao

Corresponding author: doctorhaochang@sohu.com (H.C.); fj284@cam.ac.uk (F.J.); tianlongli@hit.edu.cn (T.L.); jzhao@hit.edu.cn(J.Z.)

The PDF file includes:

Supplementary Text

Figures S1 to S39

Movies S1 to S7

Other Supplementary Material for this manuscript includes the following:

Movies S1 to S7

Movie S1 (.MP4 format). PTRMs move along the predefined HIT trajectory under RMF.

Movie S2 (.MP4 format). PTRMs move on the real *ex vivo* gastric surface under RMF.

Movie S3 (.MP4 format). PTRMs move on the real *ex vivo* intestinal surface under RMF.

Movie S4 (.MP4 format). PTRMs undergo shell degradation in response to an alkaline environment.

Movie S5 (.MP4 format). After targeting tumor cells, PTRMs achieve phased drug release at the 0.5 h and 6 h time points.

Movie S6 (.MP4 format). PTRMs move in the *in vivo* intestinal tracts under RMF.

Movie S7 (.MP4 format). PTRMs achieve phased drug release in the *in vivo* intestinal tracts under RMF.

SUPPLEMENTARY TEXT

Section S1. Methods

In vitro motion of PTRMs

The controlled magnetic-field-driven motion of PTRMs was achieved by a uniform RMF generated by a vision-based magnetic navigation system. Sucrose solutions with mass fractions of 10%, 20%, 30%, 40%, 50%, 60%, 70%, and 80% were prepared to simulate different GI mucus environments. These solutions were placed beneath a three-axis Helmholtz coil, and the motion of PTRMs driven by the RMF was observed under an Olympus microscope. The motion behavior of PTRMs was recorded by a charge-coupled device (CCD) camera mounted on the microscope and analyzed using ImageJ software. The applied magnetic field strength ranged from 0 to 15 mT, and the frequency varied from 0 to 50 Hz. COMSOL software was employed to conduct fluid simulations of the PTRMs' motion.

Regarding the study of motion on the real GI surface, fresh GI tracts were dissected from mice. After being rinsed with 0.9% normal saline, the inner surfaces were carefully unfolded using a surgical blade and placed under the microscope to observe the motion of PTRMs under the RMF. The motion behavior of the PTRMs was recorded by a CCD camera mounted on the microscope and analyzed using ImageJ software.

In vitro drug release and cell killing of PTRMs

Drug release kinetics under physiological conditions: PTRMs were incubated in pre-warmed phosphate-buffered saline (PBS, pH 7.4) maintained at 37 °C to simulate physiological conditions. The disintegration of the calcium alginate outer membrane and the subsequent sequential release of the two gelatin cores (GMS 1 and GMS 2) were monitored in real-time using an inverted microscope (Olympus). The time points of shell disintegration and core rupture were recorded to assess the pH-responsive and time-programmed release behavior.

Gelatin core rupture time analysis: To systematically evaluate the influence of gelatin concentration and magnetic nanoparticle size on drug release kinetics, a series of gelatin microspheres were prepared by mixing gelatin at concentrations of 2.5%, 5%, 7.5%, 10%, and 12.5% with iron(III) oxide nanoparticles of 20 nm, 50 nm, and 150 nm diameters, respectively. The rupture times of these microspheres were recorded during incubation in a 37 °C water bath. The release interval between GMS 1 and GMS 2 was determined to be approximately 5.5 hours under optimal formulation conditions (5% gelatin with 150 nm Fe_3O_4 for GMS 1 and 5% gelatin with 20 nm Fe_3O_4 for GMS 2).

Drug release profiling in cell culture: MC38 murine colorectal cancer cells (obtained from ATCC) were cultured in Dulbecco's Modified Eagle Medium (DMEM) supplemented with 10% fetal bovine serum (FBS) and maintained at 37 °C in a 5% CO_2 atmosphere. Cells were seeded in T25 flasks and allowed to form cell clones. PTRMs were introduced into the culture and magnetically guided into the cell clones using a RMF. The release of elesclomol and PD-1 inhibitor was observed under an inverted microscope. Supernatant samples (20 μL) were collected every 30 minutes for high-performance liquid chromatography (HPLC) and enzyme-linked immunosorbent assay (ELISA) to quantify the release profiles of elesclomol and PD-1 inhibitor, respectively.

Identification of the optimal time interval for sequential drug release: The tumoricidal efficacy of PTRMs with different sequential release intervals was evaluated using 24-well Transwell chambers fitted with 3 μm porous polycarbonate membranes, which enable transmigration of immune cells. MC38 colorectal cancer cells were seeded in the lower chamber at a density of 1×10^4 cells per well and allowed to adhere overnight. Freshly isolated human peripheral blood mononuclear cells

(PBMCs) were then added to the upper chamber at a tumor cell-to-PBMC ratio of 1:3. PBMCs could migrate across the porous membrane into the lower chamber to interact with tumor cells and exert cytotoxic effects. After incubation for the preset durations, the viability of tumor cells in the lower chamber was quantified using a CCK-8 kit.

Assessment of cuproptosis and immunogenic cell death (ICD): To evaluate the induction of cuproptosis, MC38 cells treated with PTRMs were stained with a DLAT antibody conjugated with yellow fluorescence and a reactive oxygen species (ROS) probe labeled with red fluorescence. Samples were imaged using confocal laser scanning microscopy (CLSM) to visualize DLAT oligomerization and ROS generation. Western blot analysis was performed to detect the expression levels of DLAT, FDX1, and LIAS proteins. For ICD assessment, a clonogenic assay was conducted to evaluate the cytotoxic effects of sequential drug release. MC38 cells were subjected to six treatment conditions: PBS, Co-release of elesclomol and PD-1 inhibitor, Co-release with immune cells, Phase 1 release only, 5.5h after Phase 1 release followed by combined immune cell treatment, and 5.5h after Phase 1 release followed by combined immune cells treatment plus Phase 2 drug release. Colonies were stained with crystal violet and counted 12 hours post-treatment.

Immune cell recruitment analysis: To investigate cuproptosis-mediated immune cell infiltration, tumor spheroids were co-cultured with macrophages (stained red) and T cells (stained green) for 6 hours following cuproptosis induction. Infiltration of $CD8^+$ T cells and M1 macrophages was visualized using CLSM. Correlation analysis between cuproptosis-related genes and immune cell infiltration was performed based on human CRC transcriptome data to validate the translational relevance of the findings.

Western blotting

For detection of DLAT oligomers, cells were lysed in ice-cold RIPA buffer supplemented with protease inhibitor cocktail, 1 mM PMSF and 10 mM N-ethylmaleimide (NEM) to preserve endogenous disulfide bonds. Protein samples (30 μg per lane) were mixed with non-reducing SDS loading buffer (without β-mercaptoethanol or DTT) and loaded directly without boiling. SDS-PAGE was performed on 10% gels under ice-bath conditions to prevent thermal dissociation of DLAT oligomers, followed by electrotransfer onto 0.22 μm PVDF membranes.

For detection of FDX1, LIAS and the internal reference β-actin, standard reducing Western blotting was performed. Protein samples were mixed with reducing SDS loading buffer containing 5% β-mercaptoethanol, boiled at 100 °C for 10 min, separated by 10% SDS-PAGE, and transferred onto 0.22 μm PVDF membranes.

After blocking with 5% skim milk in TBST for 1 h at room temperature, membranes were incubated overnight at 4 °C with the following primary antibodies: anti-DLAT (Proteintech, 13426-1-AP, 1:1000), anti-FDX1 (Proteintech, 12592-1-AP, 1:1000), anti-LIAS (Proteintech, 15719-1-AP, 1:1000) and anti-β-actin (Cell Signaling Technology, 4970, 1:2000). Membranes were then incubated with HRP-conjugated secondary antibody (1:5000) for 1 h at room temperature, and protein bands were visualized using an enhanced chemiluminescence (ECL) system.

***In vivo* motion of PTRMs**

The *in vivo* locomotion and targeted delivery of PTRMs were assessed through a combination of non-invasive fluorescence imaging and direct optical observation enabled by local tissue clearing. To facilitate transparent visualization of the GI tract, a tartrazine-based clearing protocol was employed. Specifically, following depilation of the abdominal region, anesthetized mice received a topical application of a tartrazine solution to the abdominal skin. This procedure effectively clears the local abdominal tissues and renders the intestinal wall transparent, allowing for direct stereomicroscopic observation of the PTRMs within the intestinal lumen. Simultaneously, for

quantitative tracking of distribution, the PTRMs were pre-labeled with the near-infrared dye DiR. Mice, fasted for 24 hours, were orally administered a suspension of PTRMs. They were then allocated into two groups (*n* = 3): one subjected to an external RMF and a control group without magnetic field. The biodistribution of PTRMs was monitored at 1h, 2h, 4h, 6h, and 12h post-administration using an *in vivo* fluorescence imaging system (IVIS). The fluorescence intensity in the colorectum and cecum (tumor site) was quantified using the instrument's software. This dual-method approach provided both macroscopic, quantitative distribution data and direct, visual confirmation of PTRM locomotion and magnetic control within the native GI environment.

Establishment of orthotopic CRC model in mice

6–8-week-old female C57BL/6 mice were housed in a specific pathogen-free (SPF) environment. For model establishment, mice were anesthetized with 2% isoflurane; the abdominal area was depilated, disinfected, and a midline incision was made to expose the cecum. A total of 1×10^6 MC38 murine CRC cells (resuspended in 50 μL PBS) were injected into the submucosal layer of the cecal wall using a 30G needle. The injection site was compressed for 30 seconds to prevent leakage, after which the abdominal wall was sutured in layers. Postoperatively, mice were monitored for general condition and tumor progression.

***In vivo* tumor ablation by PTRMs**

The mice bearing colorectal tumors were randomly divided into five groups (*n* = 3), and were subjected to treatments with PBS, intravenous injection of PD-1 inhibitor and elesclomol *via* the tail vein, oral administration of PD-1 inhibitor and elesclomol, oral administration of passive PTRMs, and oral administration of PTRMs with magnet-driven targeted therapy, respectively. Twenty-four hours after the treatments, the tumor tissues of the mice in each group were surgically resected. After being embedded in paraffin and sectioned, the sections were co-incubated with DLAT antibody and ROS probe, respectively, and then observed under a CLSM.

In addition, the tumor tissues of the mice were digested with collagenase and ground. Subsequently, they were co-incubated with flow cytometry antibodies against CD86, CD206, CD8, and CD4 for 15min. Then, the samples were analyzed using a flow cytometer, and the flow cytometry data were processed and analyzed with FlowJo software.

***In vivo* transcriptome sequencing and analysis**

The tumor tissues of the oral PTRMs magnet-driven targeted treatment group and the PBS group were subjected to transcriptome sequencing. Quality control and filtering were performed on the obtained raw data to remove low-quality reads and adapter sequences. Tools like FastQC were used to assess the data quality, and data cleaning was carried out through Trimmomatic [1,2]. The pre-processed reads were aligned with the reference genome, and the alignment analysis was conducted using the HISAT2 software to determine the position of each read on the genome [3]. Based on the alignment results, the StringTie software was employed to conduct a quantitative analysis of gene expression levels and calculate the FPKM (Fragments Per Kilobase of transcript per Million mapped reads) value for each gene [4].

The R package DESeq2 was used to perform a differential analysis on the gene expression data of the two groups of samples, and genes with significantly different expression between the PTRMs magnetic field-driven targeted treatment group and the PBS group were screened out. The fold change ($|\log_2 FC|>1$) and the adjusted P-value (padj<0.05) were set as the screening criteria. The ggplot2 package in the R language was utilized to draw a volcano plot. The expression values of cuproptosis-related genes and cytokine-related genes in the two groups of samples were extracted from the gene expression quantification results, and statistical analysis was performed using GraphPad Prism. In the R environment, the clusterProfiler package was used to conduct KEGG and

GO enrichment analyses, and the GSVA package was used to perform pathway enrichment analysis on the samples.

***In vivo* therapeutic efficacy of PTRMs**

The therapeutic efficacy of PTRMs was evaluated using a C57 mouse orthotopic CRC model. C57 mice were randomly divided into five experimental groups. On Day 0, 3×10^6 luciferase (LUC)-labeled MC38 cells were surgically injected into the cecal submucosa of each mouse to establish orthotopic colorectal tumors.

Treatments were implemented on Day 5, Day 10 and Day 15 post tumor inoculation, corresponding to the five groups: (1) PBS control group; (2) intravenous combination group (tail vein injection of free elesclomol + free PD-1 inhibitor); (3) free oral combination group (oral gavage of free elesclomol + free PD-1 inhibitor); (4) passive PTRM group (oral administration of non-magnetically guided PTRMs); (5) magnetic-targeted PTRM group (oral PTRMs combined with external magnetic field guidance). To eliminate confounding variables for cross-group comparison, all groups received identical total drug doses per mouse: 11.3 μg elesclomol and 9.4 μg PD-1 inhibitor per treatment cycle. For PTRM-containing groups, each mouse was orally given 1×10^6 drug-loaded PTRMs per administration, where every 1×10^6 PTRMs co-encapsulated 11.3 μg elesclomol and 9.4 μg PD-1 inhibitor, consistent with the uniform systemic drug dosage adopted in free-drug treatment groups.

On Day 30 post tumor inoculation, all mice were sacrificed, and primary tumor tissues as well as major visceral organs were harvested. Harvested tissues were fixed, paraffin-embedded, sliced, and subjected to H&E staining and immunohistochemical analysis for subsequent pathological assessment. Throughout the 30-day observation period, intraperitoneal injection of D-luciferin potassium salt was performed every 5 days, and in *vivo* bioluminescence imaging was applied to dynamically monitor tumor growth. The body weight of each mouse was recorded once every 3 days, and all mouse survival events were tracked to generate overall survival curves.

Section S2. External magnetic field setup

Magnetic generation consists of a three degrees of freedom Helmholtz coil, a pair of vertically arranged coils, multifunction data acquisition (DAQ NI-PCI-6259) and four single-channel output power amplifiers. The drive signal generated by DAQ was amplified by the amplifiers and excited the coil to generate magnetic fields. The Helmholtz coil generated a rotating magnetic field to achieve the propulsion and retention of the microrobot. The giant three-orthogonal Helmholtz coils pair used the same drive system to navigate the locomotion of the microrobot *in vivo*

SUPPLEMENTARY FIGURES

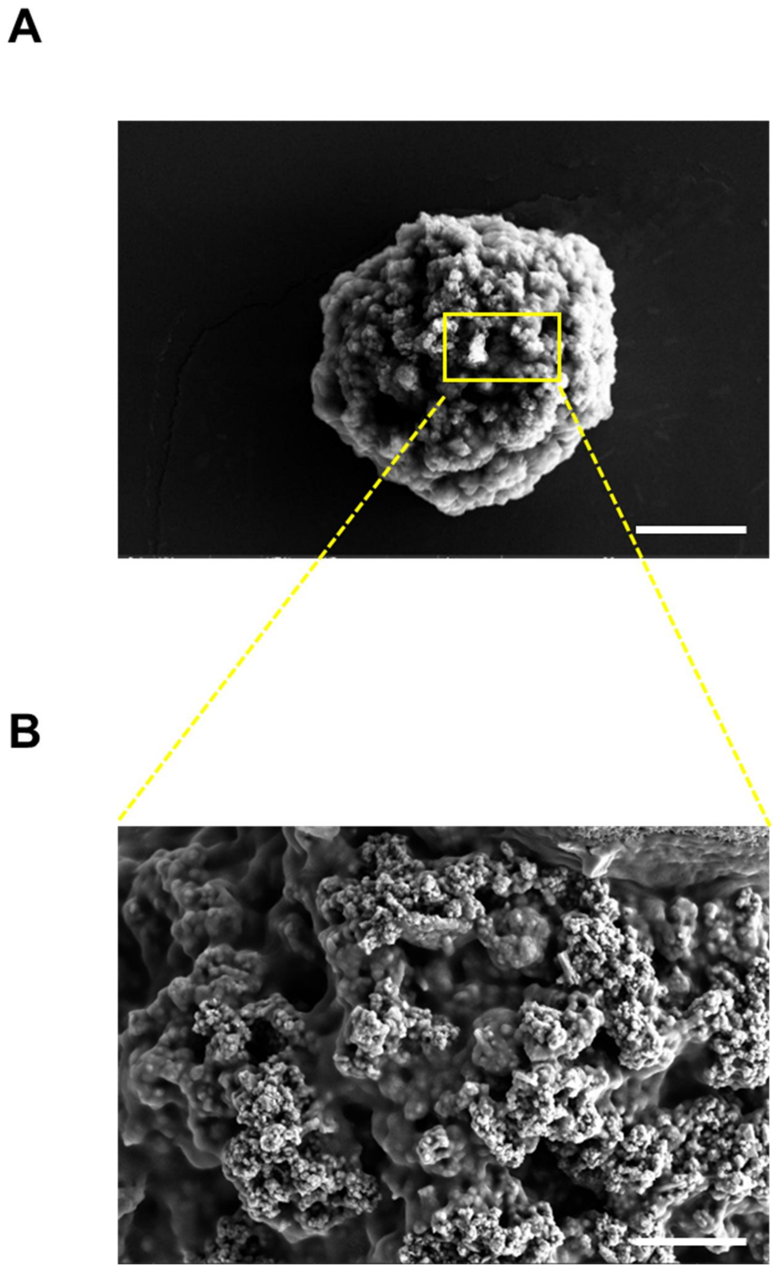

Fig. S1. (A) SEM characterization of gelatin microsphere at 2500× magnification. Scale bar, 50 µm. (B) SEM characterization of GMS at 10,000× magnification. Scale bars (top), 15 µm. Scale bar (bottom), 5 µm. This scanning electron microscopy (SEM) characterization validates the structural properties of gelatin microspheres (GMS)—a core component of programmable temporal-responsive microrobots (PTRMs, synthesized via one-step microfluidic chip)—by capturing both macro-morphology and micro-surface details that underpin PTRMs' functional performance.

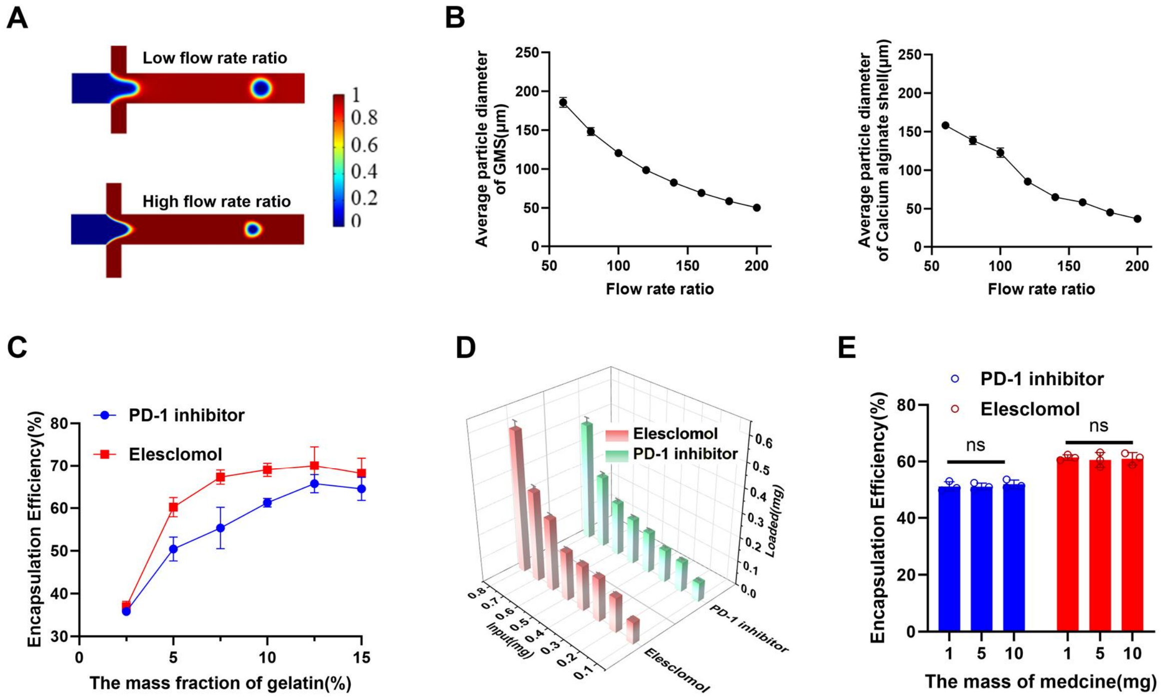

Fig. S2. Screening and optimization of key fabrication parameters for the PTRM system. **(A)** CFD simulation of droplet formation in the microfluidic chip at low and high flow rate ratios, showing the shear-driven droplet generation process. **(B)** Effect of flow rate ratio on the average particle diameter of GMS core (left) and calcium alginate shell (right). **(C)** Encapsulation efficiency of elesclomol and PD-1 inhibitor under different gelatin mass fractions. **(D)** Three-dimensional histogram displaying the actual drug loading of PTRMs corresponding to different input dosages of elesclomol and PD-1 inhibitor. (E) Encapsulation efficiency of the two drugs at different initial drug input masses; ns indicates no statistically significant difference.

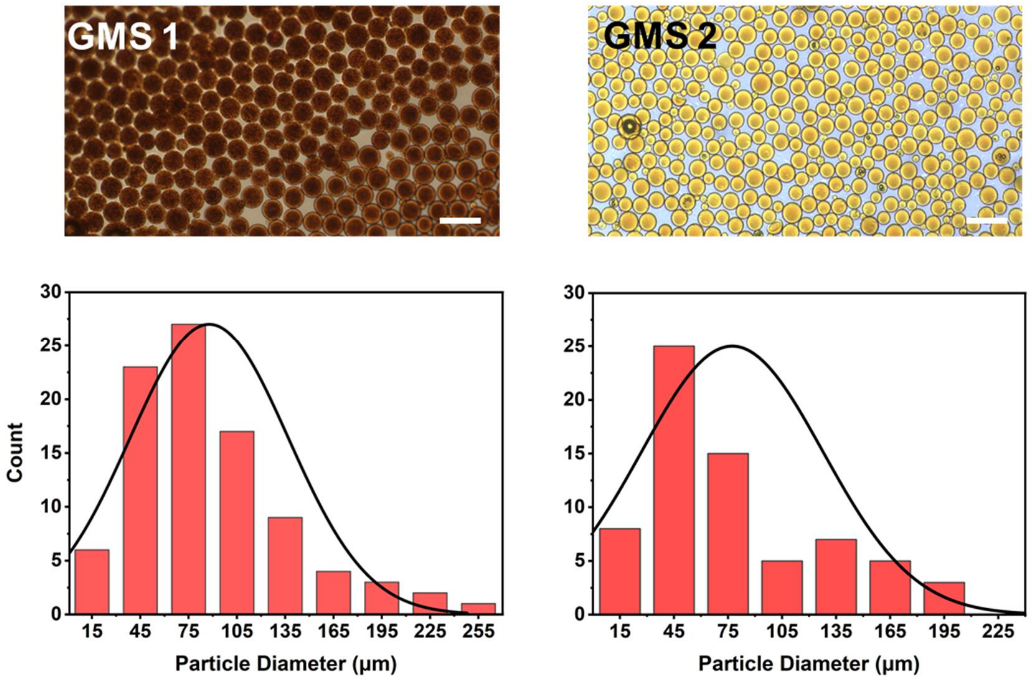

Fig. S3. Bright field images and particle size distributions of GMS 1 (left) and GMS 2 (right). Scale bar, 150 μm. For GMS 1 (left panel): The bright-field micrograph displays densely packed, uniformly shaped brown particles with tight interparticle spacing, exhibiting high morphological monodispersity. The corresponding particle size distribution (red histogram = count frequency; black curve = distribution profile) spans a diameter range of ~15–255 μm, with the count peak centered at 75–105 μm. Particle frequency rises to this peak before declining gradually; large-diameter particles (>195 μm) are present in negligible quantities. For GMS 2 (right panel): Its bright-field micrograph shows light-yellow particles dispersed in a translucent matrix, retaining consistent morphological regularity. The size distribution covers a comparable diameter range (~15–225 μm) and shares a count peak at 75–105 μm, but differs in the tail region: the frequency decay of mid-to-large-diameter particles (⩾105 μm) is gentler than that of GMS 1, indicating subtle heterogeneity in size population.

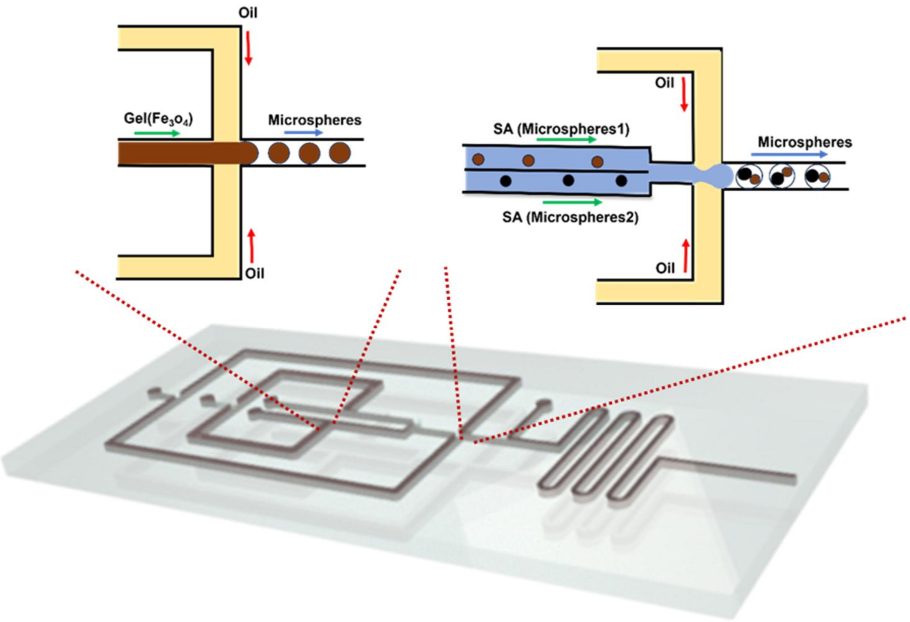

Fig. S4. Schematic diagram of a one-step microfluidic chip for PTRMs synthesis. One-step microfluidic chip represents a streamlined platform for programmable temporally responsive microrobot (PTRM) synthesis: its integrated microchannel architecture addresses the batch-to-batch heterogeneity of multi-step fabrication, enabling precise co-encapsulation of sodium alginate (SA) shell, GMS1 and GMS2 in a single fluidic process. This design not only standardizes PTRM structural uniformity but also pre-programs two critical functionalities—magnetic actuation (for targeted navigation) and sequential drug release (for synergistic therapy)—directly during synthesis, bridging the gap between lab-scale microrobot fabrication and translatable, application-ready systems for coordinated cancer treatment.

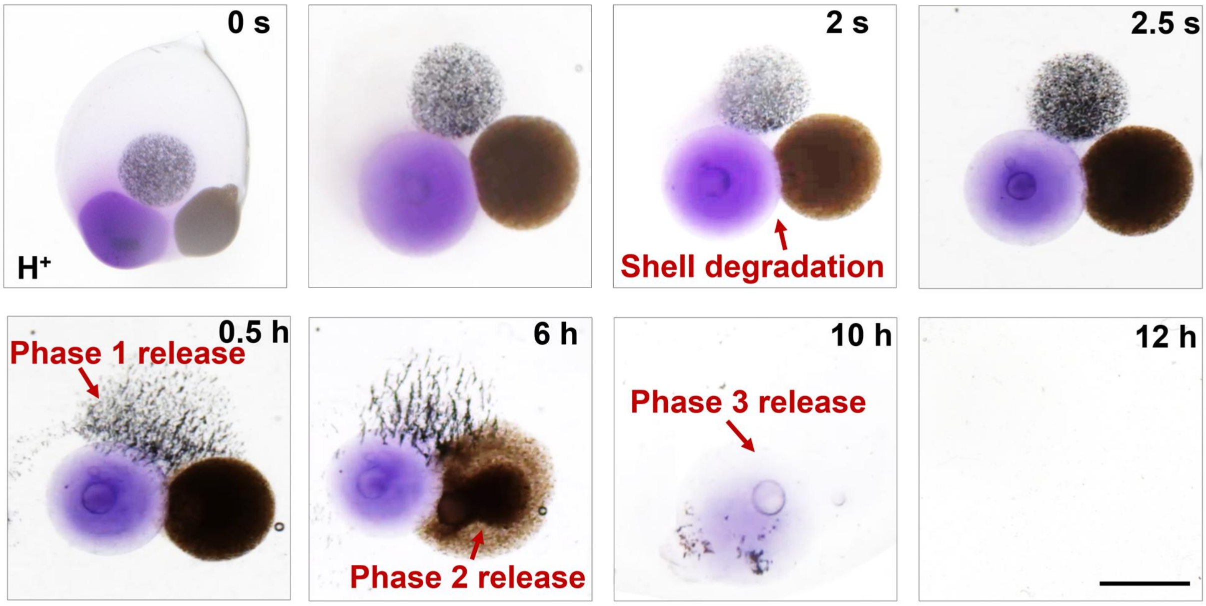

Fig. S5. ***In vitro*** **validation of time-sequential multistage release from triple-core PTRM microrobots. Scale bar, 60 µm.**

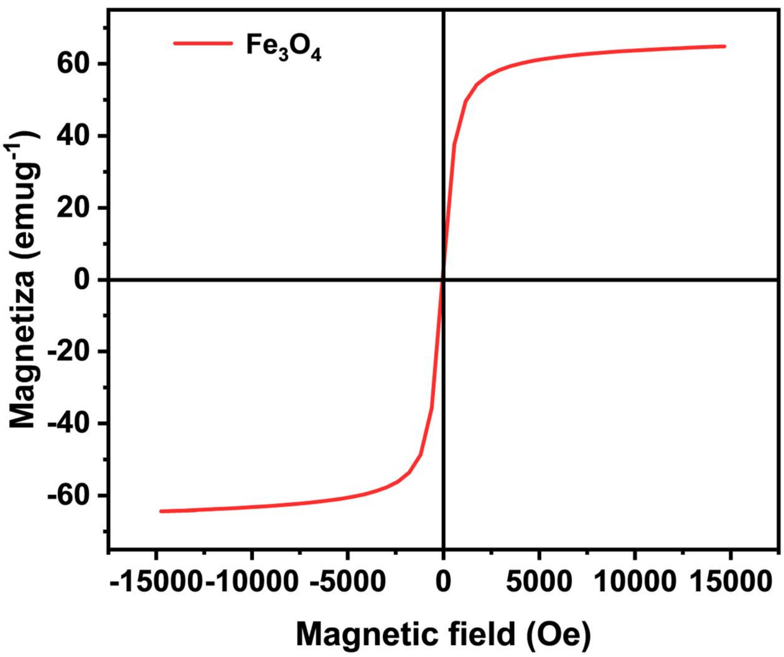

Fig. S6. Magnetic hysteresis loops of Fe_3O_4, measured at 300 K using a vibrating sample magnetometer (VSM), exhibiting the saturation magnetization (M_s), coercivity (H_c), and remanence (M_r) characteristics. The loop exhibits defining soft magnetic characteristics: a saturation magnetization (Ms) of ~60 emu g^{-1} (approaching the bulk Fe_3O_4 value, signifying strong magnetic responsiveness), near-zero coercivity (Hc), and minimal remanence (Mr). These metrics are functionally critical for PTRMs: the high Ms ensures sufficient magnetic torque/force to enable precise navigational control via external magnetic fields, while the low Hc and Mr prevent irreversible magnetic aggregation of the Fe_3O_4-doped gel core. This stability is essential for maintaining the microrobots' dispersibility in biological fluids, avoiding clumping that would disrupt targeted delivery or sequential drug release. For the PTRM platform, this Fe_3O_4 component acts as the magnetic actuation backbone; its validated magnetic properties confirm the microrobots can be reliably manipulated (e.g., guided to colorectal tumor sites) while preserving structural uniformity—laying the groundwork for their translational utility in coordinated cancer therapy.

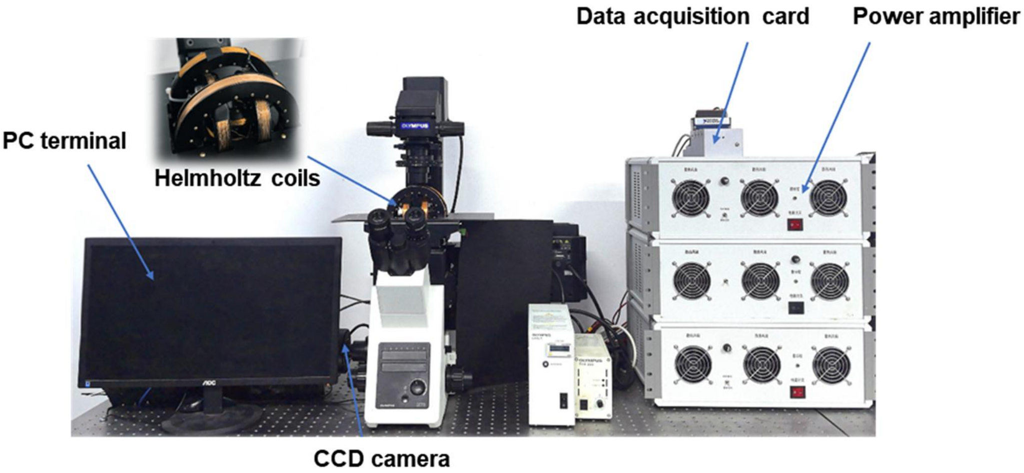

Fig. S7. Integral experimental system. The system contains a PC terminal, Helmholtz coils, a CCD camera, a data acquisition card and a power amplifier. An integrated closed-loop experimental system was established, uniting tunable magnetic actuation, real-time high-resolution imaging, and synchronous data acquisition for colorectal cancer-targeted delivery. A PC terminal serves as the core control hub, generating customizable magnetic field profiles (magnitude, direction, and frequency) to direct PTRM locomotion while processing real-time footage from a microscope-integrated CCD camera for adaptive actuation tuning. A multi-axis Helmholtz coil assembly (inset) produces uniform, dynamic magnetic fields that interact with the PTRMs' Fe_3O_4-doped gel core—leveraging its ~50 emu g^{-1} saturation magnetization—to enable precise targeted maneuvering. These magnetic fields are modulated via a high-current power amplifier, with signal synchronization managed by a data acquisition card to ensure temporal alignment between control commands, field outputs, and imaging data. This setup closes the loop between programmable actuation, motion visualization, and quantitative trajectory analysis, achieving sub-millimeter navigational accuracy critical for verifying the PTRMs' ability to traverse complex colorectal cavity pathways and reach tumor sites. This capability is a prerequisite for the microrobots' sequential drug release function—avoiding chemotherapy-immunotherapy antagonism and enabling synergistic cancer therapy with 76% tumor remission, as demonstrated in *in vivo* experiments.

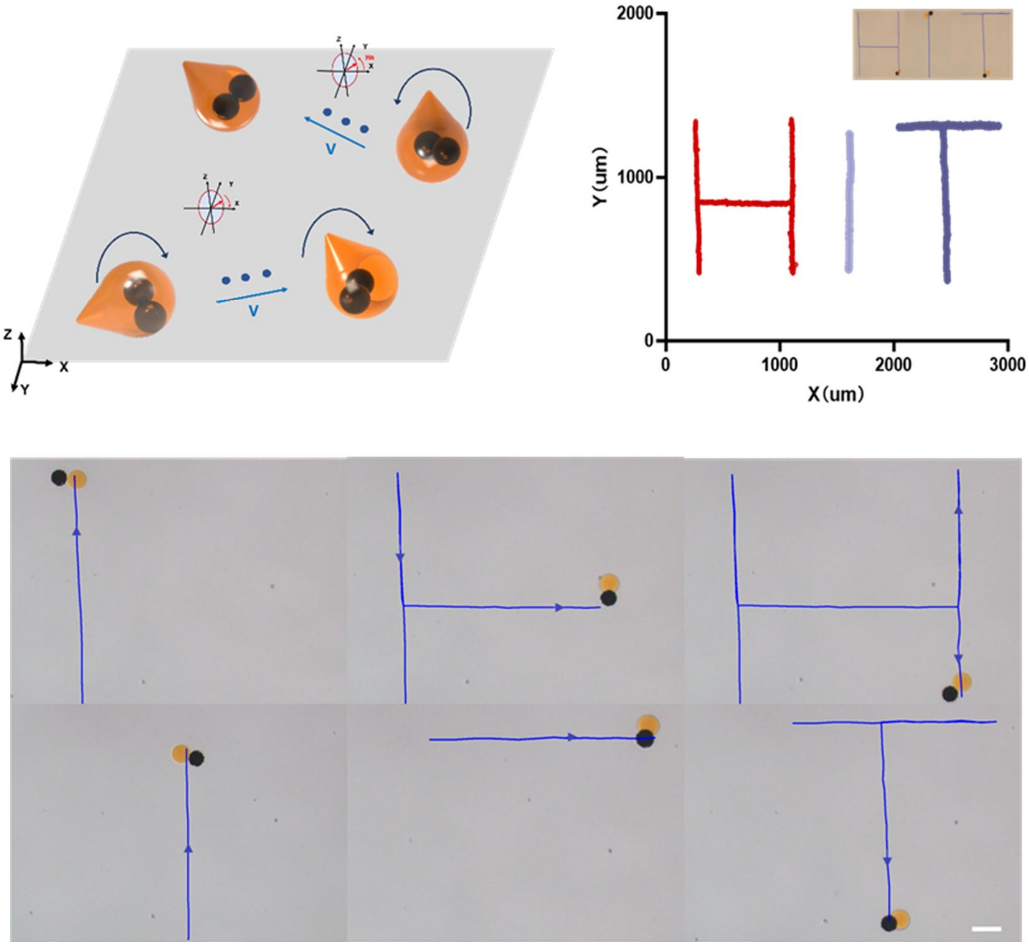

Fig. S8. Time-lapse image and trajectories illustrating controllable magnetic propulsion of PTRMs in a predefined trajectory. Scale bar, 50 µm. The top-left schematic illustrates the "HIT" path that PTRMs are programmed to follow, with directional translation vectors marking the intended locomotion sequence. The top-right plot quantifies trajectory fidelity: PTRMs trace the "HIT" pattern with a maximum positional deviation of ≤2 µm relative to the predefined path (inset outline), a precision enabled by the synergistic interplay of the Helmholtz coil assembly's tunable magnetic fields and the PTRMs' consistent soft magnetic responsiveness. This stability ensures uniform force transmission during actuation without structural degradation. The bottom time-lapse sequence captures real-time execution of the "HIT" trajectory: the PTRM (black/orange composite particle) advances along the blue predefined path in synchronized steps, with each frame aligned to magnetic field adjustments from the PC terminal. The CCD camera confirms continuous, stable propulsion while preserving the integrity of the PTRMs' SA matrix and payload-loaded microspheres—critical for maintaining the sequential release capability that mitigates chemotherapy-immunotherapy antagonism. This demonstration of "HIT" trajectory-following precision confirms PTRMs can navigate the complex luminal environment of the colorectum to reach target tumor sites, laying the groundwork for the synergistic cancer therapy that achieves 76% tumor remission in *in vivo* experiments.

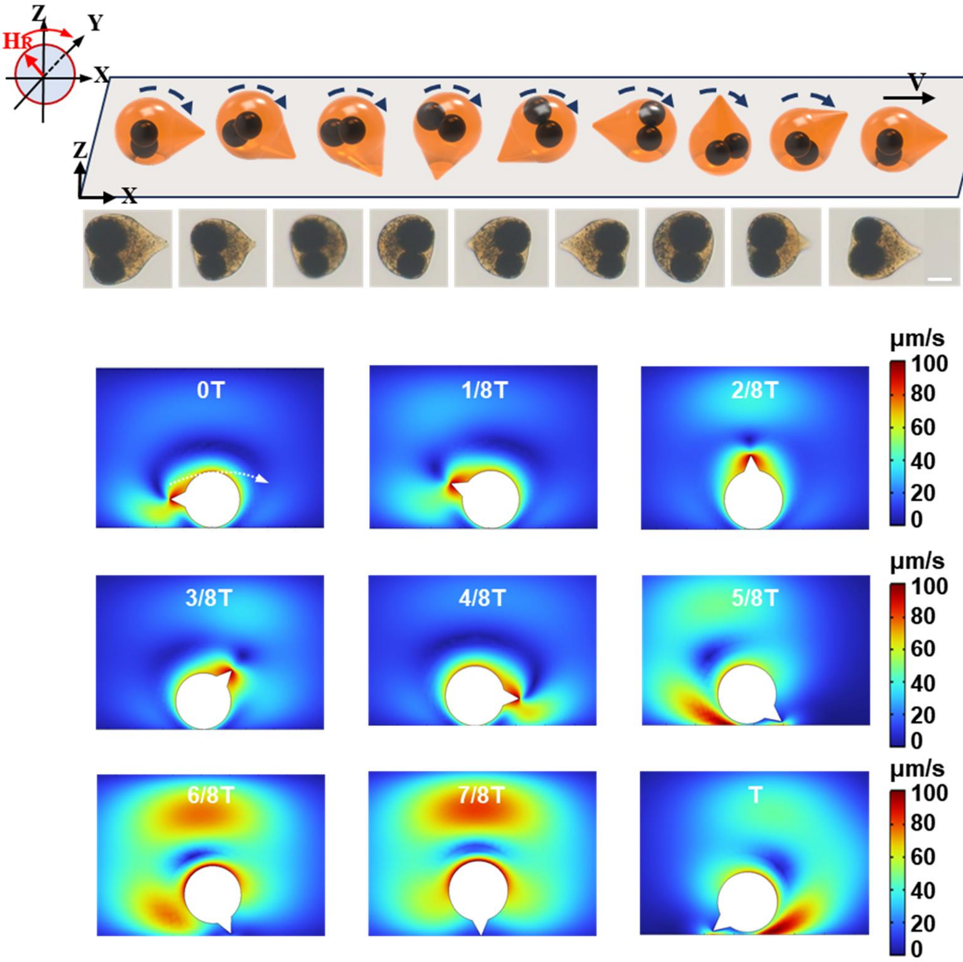

Fig. S9. Motion and simulation results of PTRMs in PBS under a rotating magnetic field. This simulation targets magnetic composite microstructures with a size of ~120 μm, conducted in a simulated physiological fluid environment (density: ~1050 kg/m³; dynamic viscosity: 3.5×10^{-3} Pa·s). A rotating magnetic field (RMF) with an intensity of ~10 mT was applied, rotating about the x-z axes, with the rotation period defined as T Via magnetic field-solid mechanics-fluid mechanics multiphysics coupling, and using a time step of 1/100 T, the rotational motion of the microstructures and the distribution of the surrounding flow field were analyzed at distinct periodic nodes (*e.g.*, 0 T, 1/8 T, 2/8 T). Scale bar, 50 μm.

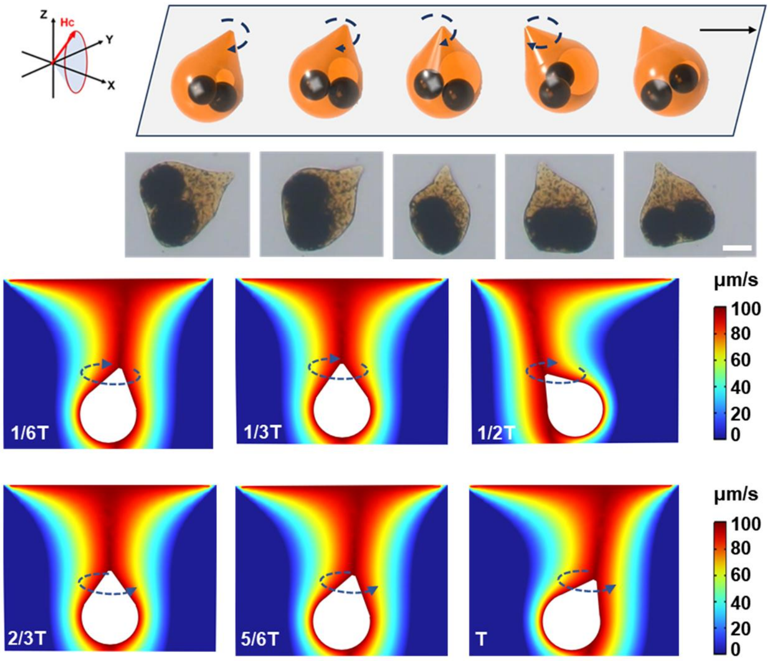

Fig. S10. Motion and simulation results of PTRMs in PBS under a conical magnetic field. This simulation focuses on magnetic composite microstructures with a size of ~120 μm, conducted in a physiological fluid environment (density: 1050 kg/m³; dynamic viscosity: 3.5×10^{-3} Pa·s). A conical magnetic field with an intensity of ~10 mT was applied, with its rotation period defined as T. Via magnetic field-solid mechanics-fluid mechanics multiphysics coupling, and using a time step of 1/100 T, the motion of the microstructures and the distribution of the surrounding flow field were analyzed at distinct periodic nodes (*e.g.*, 1/6 T, 1/3 T). Scale bar, 50 μm.

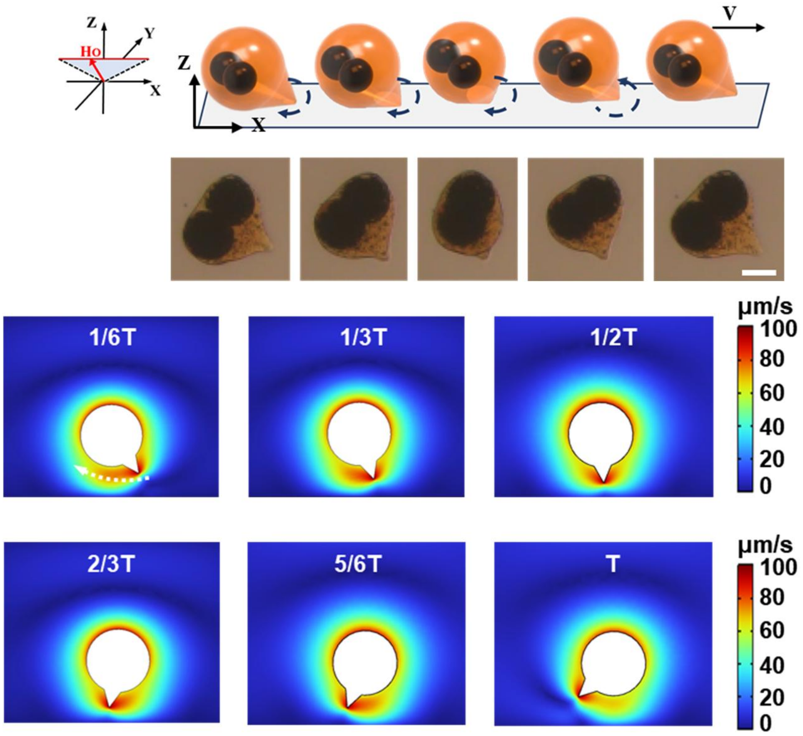

Fig. S11. Motion and simulation results of PTRMs in PBS under a gradient oscillating magnetic field. This simulation focuses on magnetic composite microstructures with a size of ~50 μm, conducted in a physiological fluid environment (density: 1050 kg/m³; dynamic viscosity: 3.5×10^{-3} Pa·s). A gradient oscillating magnetic field with an intensity of ~10 mT was applied, the time for the pendulum to move unidirectionally from its initial position to the maximum displacement on the same side without returning to the starting point—i.e., the duration of one unidirectional half-stroke oscillation—is denoted as T. Via magnetic field-solid mechanics-fluid mechanics multiphysics coupling, and using a time step of 1/100 T, the rotational motion of the microstructures and the distribution of the surrounding flow field were analyzed at distinct periodic nodes (*e.g.*, 1/6 T, 1/3 T). Scale bar, 50 μm.

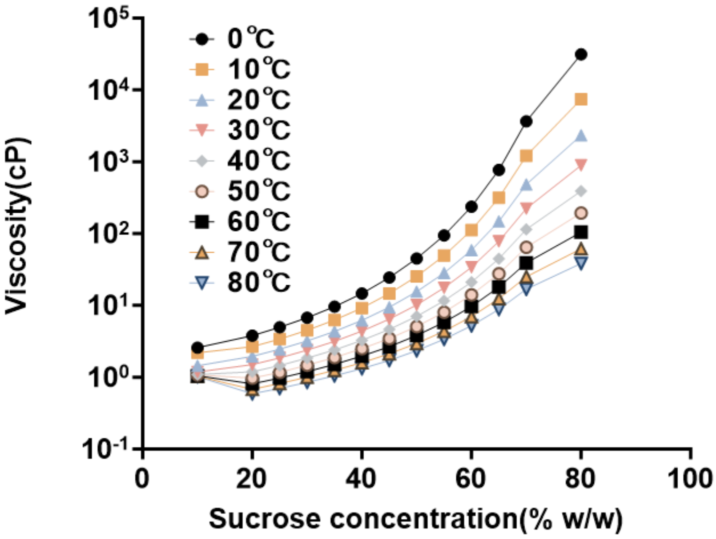

Fig. S12. Viscosity of sucrose solutions at different concentrations and temperatures. This characterization serves as a tunable fluid mimicry toolkit for evaluating the environmental adaptability of programmable temporal-responsive microrobots (PTRMs)—as the colorectal cavity's native fluids (e.g., dilute luminal liquid, mucus-rich secretions) exhibit viscosity spanning multiple orders of magnitude, shaped by content composition and physiological temperature (37°C). Two defining trends emerge from the data: at a constant temperature, viscosity rises exponentially with sucrose concentration; Conversely, at fixed concentration, higher temperatures drive sharp viscosity reduction. For PTRM development, these data enable targeted *in vitro* actuation tests: by selecting sucrose solutions with viscosity matching specific colorectal fluid environments, researchers can validate whether the microrobots retain the precise trajectory control and stable propulsion under physiologically relevant viscous resistance—ensuring the microrobots can reliably navigate to tumor sites even in the complex fluid landscapes of the colorectum, a prerequisite for their sequential drug release and synergistic therapy function.

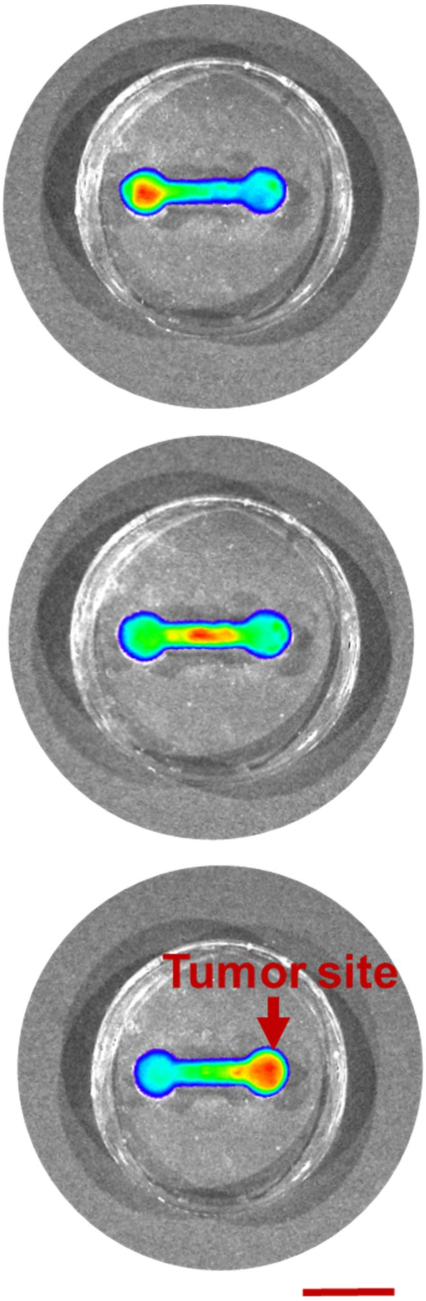

Fig. S13. Time-lapse *in vitro* live fluorescence images depicting the targeted movement of PTRMs towards tumor sites. Scale bar, 2 cm. This live-fluorescence time series validates the targeted navigation capability of programmable temporal-responsive microrobots (PTRMs) in a tumor-mimetic *in vitro* system—leveraging the magnetic actuation setup and the microrobots' Fe_3O_4-doped core to guide PTRMs (loaded with fluorescence-labeled payload microspheres) toward the designated tumor site. The blue-to-yellow-to-red fluorescent gradient tracks the PTRMs' position across sequential time points: initially anchored at the chamber's left end (top frame), the microrobots are steered by tunable external magnetic fields to translocate across the cavity, ultimately accumulating at the pre-defined "Tumor site" (bottom frame). Notably, the chamber uses a sucrose solution to replicate physiologically relevant viscous resistance, ensuring the observed movement reflects *in vivo* navigational performance. This demonstration confirms PTRMs can reliably home to tumor locations under realistic fluid conditions.

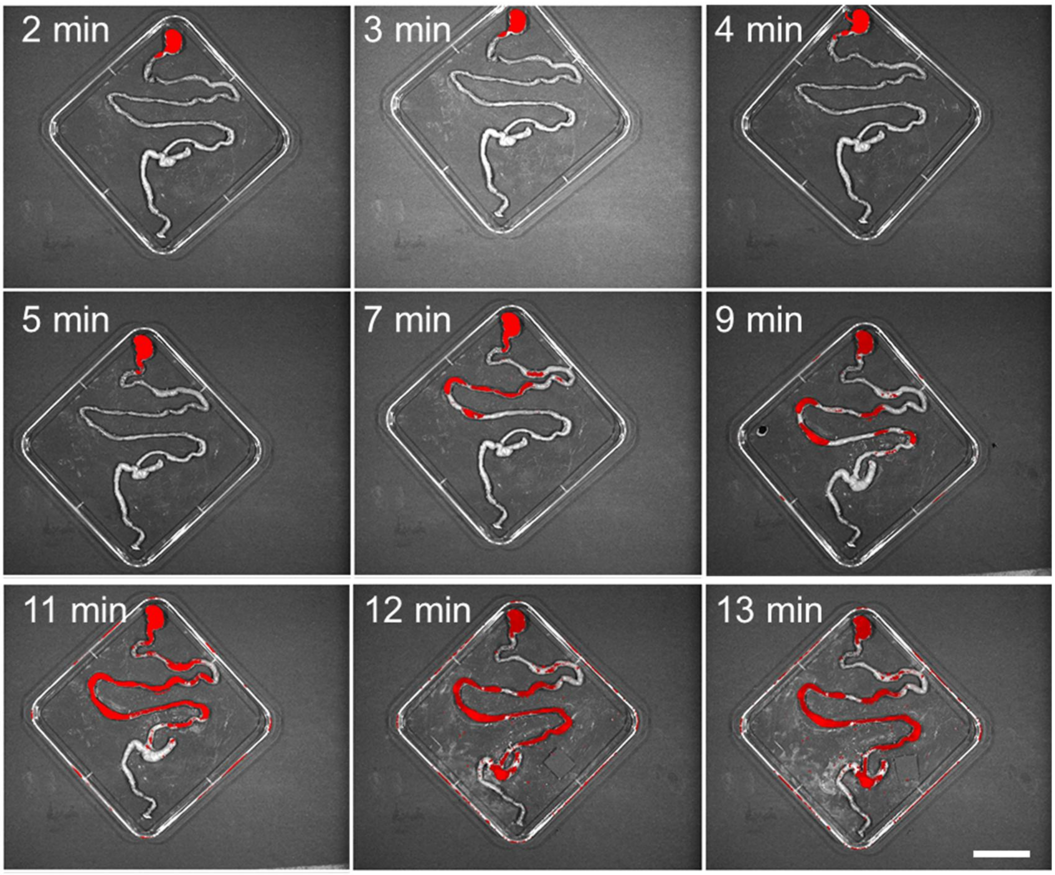

Fig. S14. Analysis of the fluorescence intensity of PTRMs moving through the stomach and intestine to reach the tumor site in the *ex vivo* gastrointestinal tract under a rotating magnetic field. Scale bar, 2 cm. This *ex vivo* experiment validates PTRMs' navigational capability in a gastrointestinal (GI) tract-mimetic model—replicating the complex, tortuous geometry of the stomach-intestine pathway and physiologically matched fluid viscosity—while leveraging a rotating magnetic field to drive movement. The time-lapse sequence tracks fluorescence-labeled PTRMs (red signal): at 2 min, PTRMs are localized at the tract's starting end; over 3–13 min, the PTRMs respond to the rotating magnetic field's torque, propelling along the winding GI path—their red fluorescence trajectory gradually covering the tortuous channel, ultimately advancing toward the target tumor region. This test bridges the gap between simplified *in vitro* models and *in vivo* applications: it confirms PTRMs can navigate the irregular, confined spaces of the GI tract (a key challenge for colorectal tumor targeting) while overcoming viscous fluid resistance, directly supporting the subsequent *in vivo* demonstration of 76% tumor remission via sequential drug release.

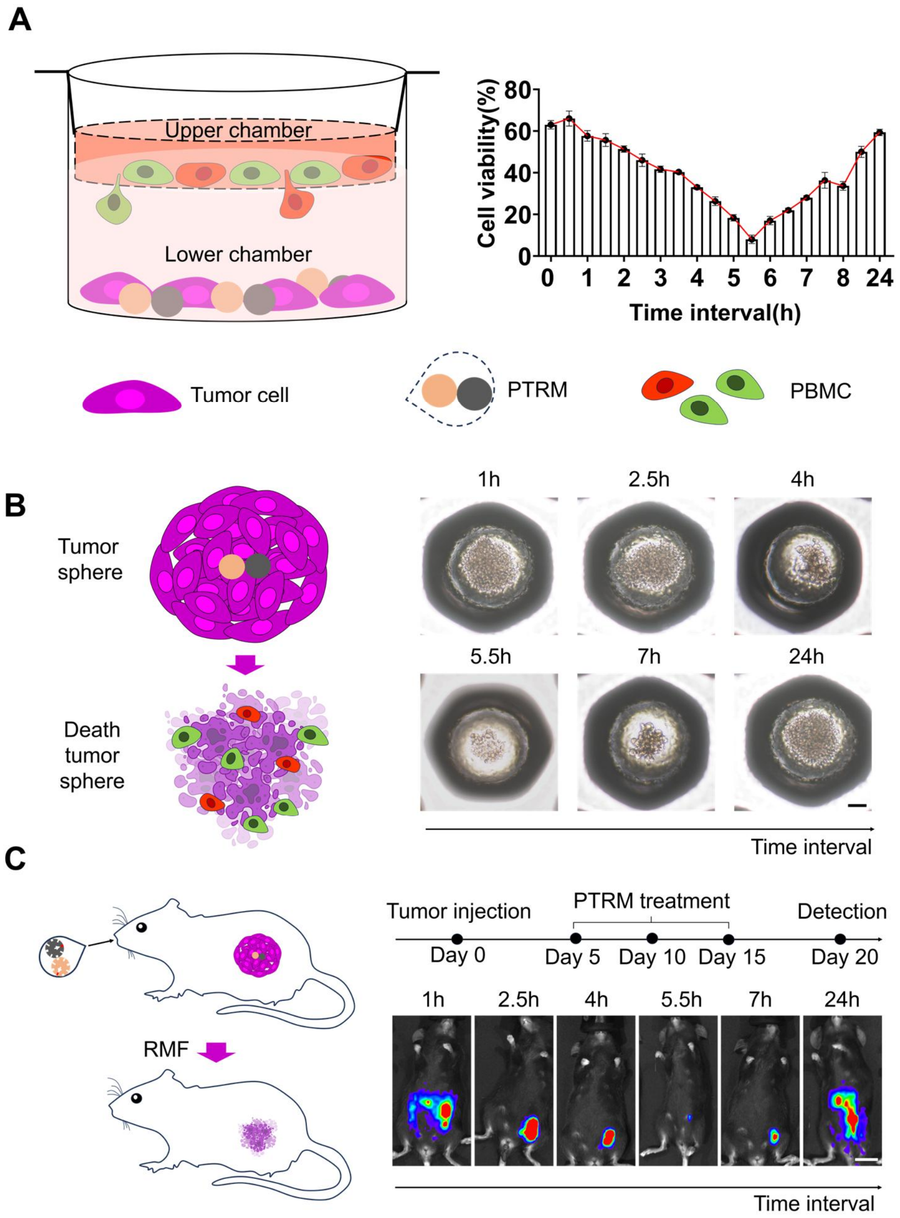

Fig. S15. Optimization of the optimal time interval for sequential therapy across *in vitro*, 3D tumor spheroid, and *in vivo* models. (A) *In vitro* evaluation of tumor cell killing efficacy under different sequential release time intervals. Left: Schematic illustration of the co-culture system of CRC cells and PBMCs treated with two-stage sequentially released PTRMs. Right: Cell viability curve of CRC cells at different time intervals (0–24 h) after treatment, showing the time-dependent cytotoxic effect with the lowest viability at 5.5 h. **(B)** 3D tumor spheroid model verification of tumor killing efficacy under different release intervals. Left: Schematic diagram of PTRM-mediated destruction of tumor spheroids co-cultured with PBMC. Right: Representative brightfield images of tumor spheroids at 1 h, 2.5 h, 4 h, 5.5 h, 7 h, and 24 h after PTRM treatment, demonstrating the most severe structural disruption at the 5.5 h time point. Scale bar, 50 µm. **(C)** *In vivo* validation of anti-tumor efficacy of PTRMs with different sequential release intervals. Left: Schematic of oral PTRM administration under rotating magnetic field (RMF) guidance in CRC tumor-bearing mice. Middle: Timeline of *in vivo* treatment and detection. Right: Representative *in vivo* fluorescence imaging of mice at different time intervals (1 h to 24 h) after PTRM treatment, confirming the optimal therapeutic effect at the 5.5 h time point. Scale bar, 2 cm.

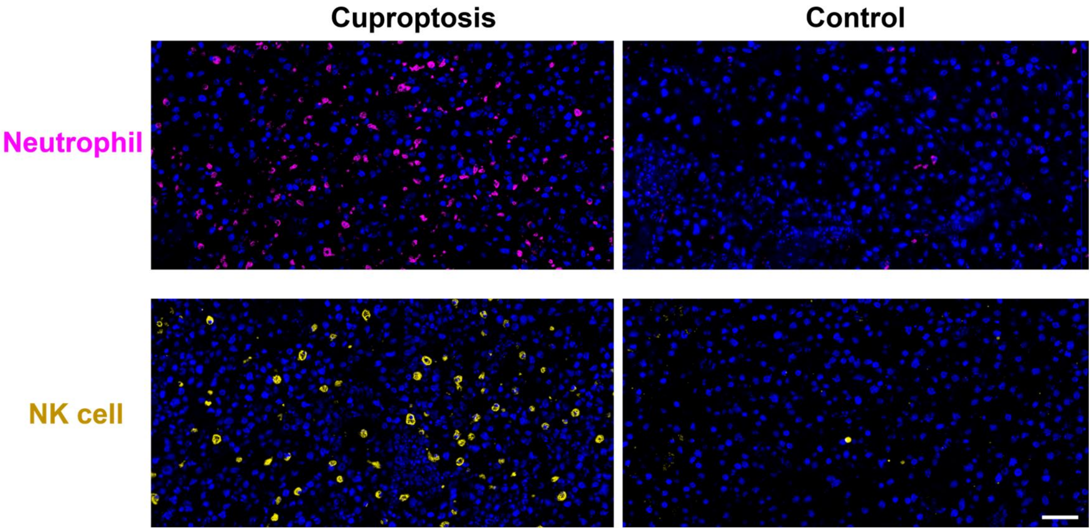

Fig. S16. Immunofluorescence staining of neutrophils and NK cells in the cuproptosis group and control group. Representative immunofluorescence images showing the infiltration of neutrophils (pink fluorescence) and NK cells (yellow fluorescence) in the cuproptosis group and control group. Blue fluorescence indicates DAPI-stained nuclei. Scale bar is indicated in the bottom right corner. Scale bar, 40 μm.

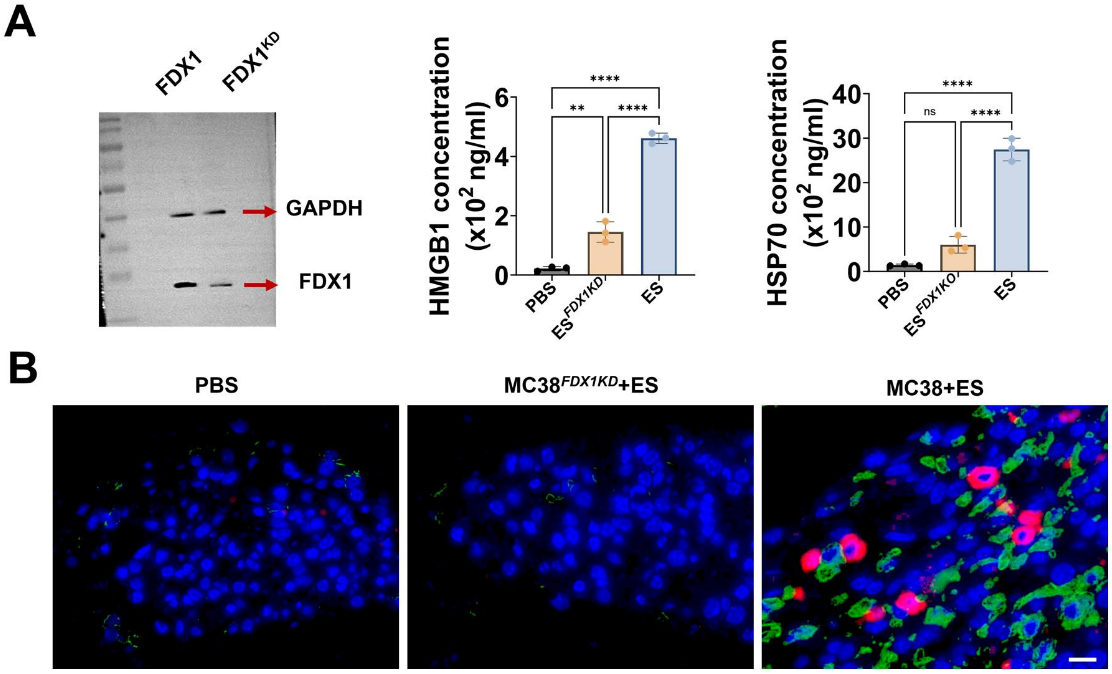

Fig. S17. Cuproptosis induces DAMP release from tumor cells and drives anti-tumor immune cell infiltration. (A) Cuproptosis triggers the release of damage-associated molecular patterns (DAMPs) in MC38 cells. The concentrations of the canonical DAMPs HMGB1 (left) and HSP70 (right) in the cell culture supernatant of different treatment groups were measured by ELISA. Groups included: PBS control, FDX1-knockdown ($FDX1^{KD}$) MC38 cells treated with ES (ES^{FDX1KD}), and wild-type MC38 cells treated with ES (ES). Data are presented as mean ± SD, n = 3; $^{**}p < 0.01$, $^{****}p < 0.0001$, ns = not significant. **(B)** Cuproptosis-induced DAMPs drive immune cell infiltration in the tumor microenvironment. Representative immunofluorescence images showing the infiltration of macrophages (green) and T cells (red) in tumor tissues from the PBS control group, wild-type MC38 tumor-bearing mice treated with ES (MC38+ES), and FDX1-knockdown MC38 tumor-bearing mice treated with ES ($MC38^{FDX1KD}$+ES). Nuclei were counterstained with DAPI (blue). Robust immune cell infiltration was exclusively observed in the MC38+ES group, while minimal infiltration was detected in both the PBS control and $MC38^{FDX1KD}$+ES groups, indicating that cuproptosis-specific DAMP release is critical for immune cell recruitment. Scale bar, 10 μm.

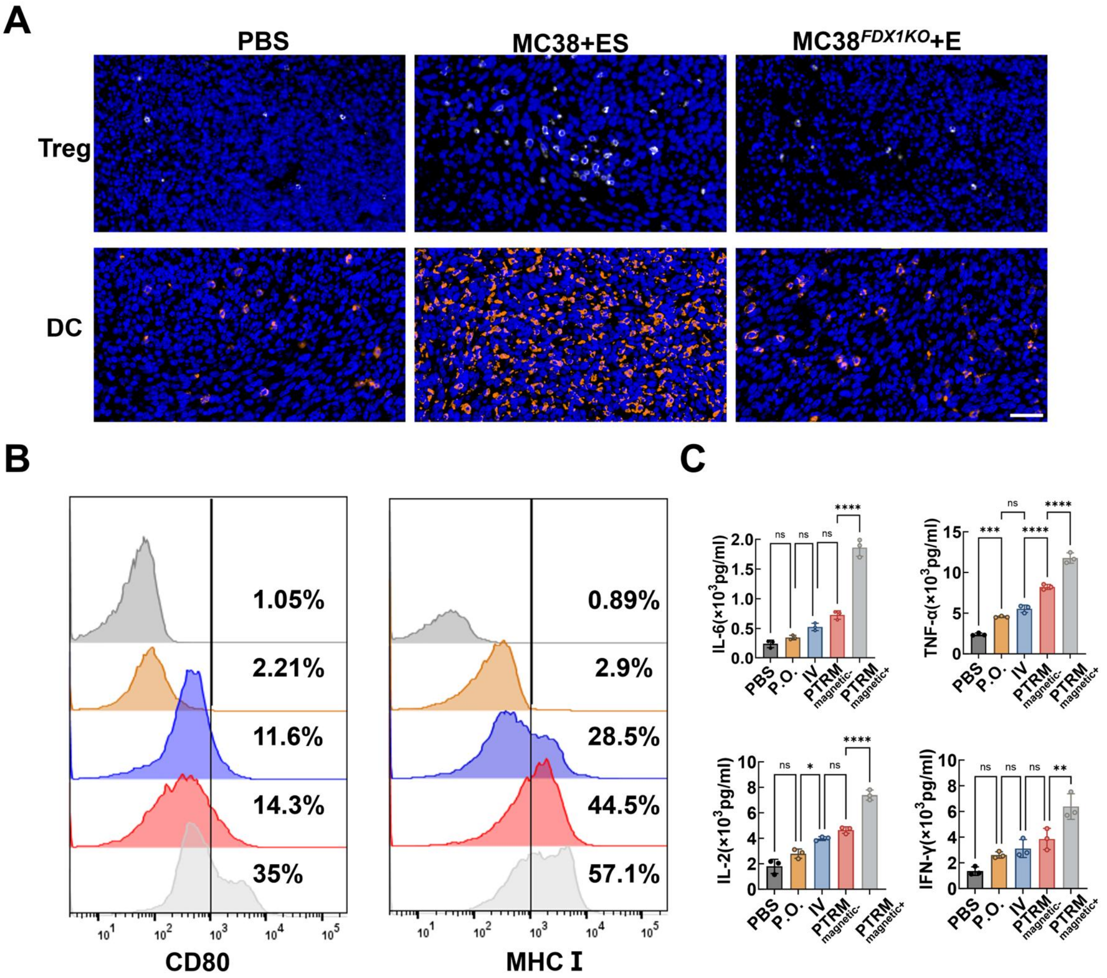

Fig. S18. Cuproptosis alters intratumoral Treg and DC infiltration and promotes dendritic cell activation. (A) Representative immunofluorescence (IF) images of tumor tissue sections showing the infiltration density and spatial distribution of regulatory T cells (Tregs) and dendritic cells (DCs) before and after cuproptosis induction. Nuclei are counterstained with DAPI (blue), while specific markers for Tregs and DCs are visualized via white and orange fluorescence, respectively. Scale bar, 40 μm. **(B)** Representative flow cytometry histograms quantifying the expression levels of activation markers CD80 (left) and MHC I (right) on the gated DC populations across the designated treatment groups. Gating thresholds and exact percentage frequencies (ranging from 0.89% to 57.1%) are designated for each monitored peak. **(C)** Enzyme-linked immunosorbent assay (ELISA) quantification of specific cytokine release profiles from the immune cells across different cohorts. Data are expressed as mean ± SD. Statistical analysis was performed using one-way ANOVA followed by Tukey's post-hoc test.

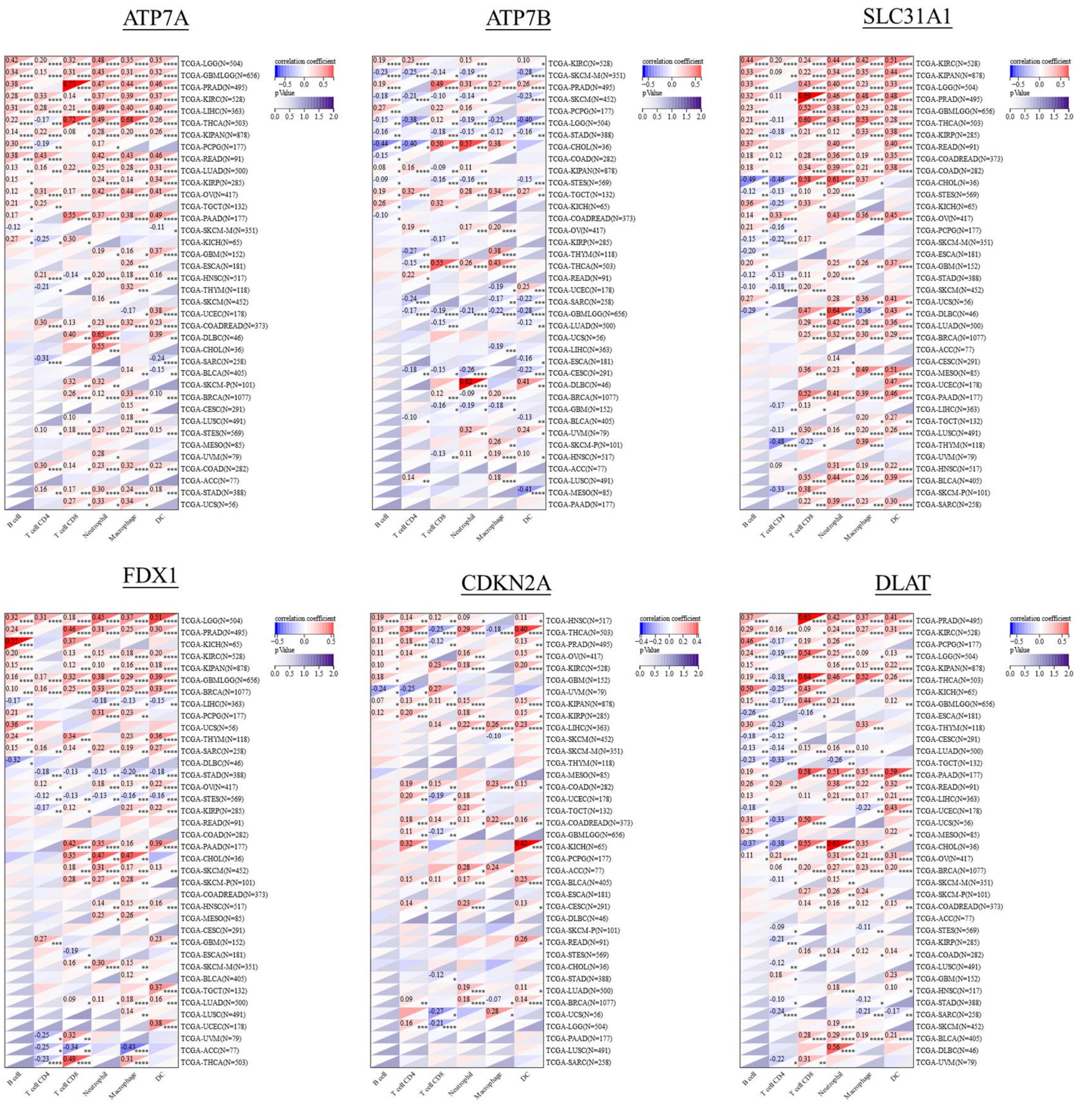

Fig. S19. Heatmap of the correlation between cuproptosis-related genes (ATP7A, ATP7B, SLC31A1, FDX1, CDKN2A, DLAT) and immune cell infiltration. This heatmap dissects the molecular crosstalk between cuproptosis-related gene expression and immune cell infiltration in the tumor microenvironment (TME)—a critical mechanistic layer supporting the synergistic chemotherapy-immunotherapy efficacy of PTRMs. Each subpanel corresponds to one cuproptosis-associated gene (e.g., ATP7A, FDX1): rows represent distinct immune cell subsets (e.g., T cell subpopulations, macrophages, dendritic cells), columns denote individual tumor samples, and the red/blue color gradient quantifies Pearson correlation coefficients (red = positive correlation, blue = negative correlation, intensity reflects magnitude). These associations reveal that cuproptosis-related genes modulate TME immune composition—upregulating anti-tumor immune infiltration while limiting suppressive populations. For PTRMs, this data provides a molecular rationale for their synergistic function: the microrobots' sequential drug release can tune the expression of these cuproptosis genes, reshaping the TME to enhance immune cell anti-tumor activity while avoiding chemotherapy-induced immune suppression. This genetic-immune regulatory axis directly underpins the 76% tumor remission rate observed in *in vivo* experiments, linking PTRMs' targeted delivery to mechanistic TME reprogramming.

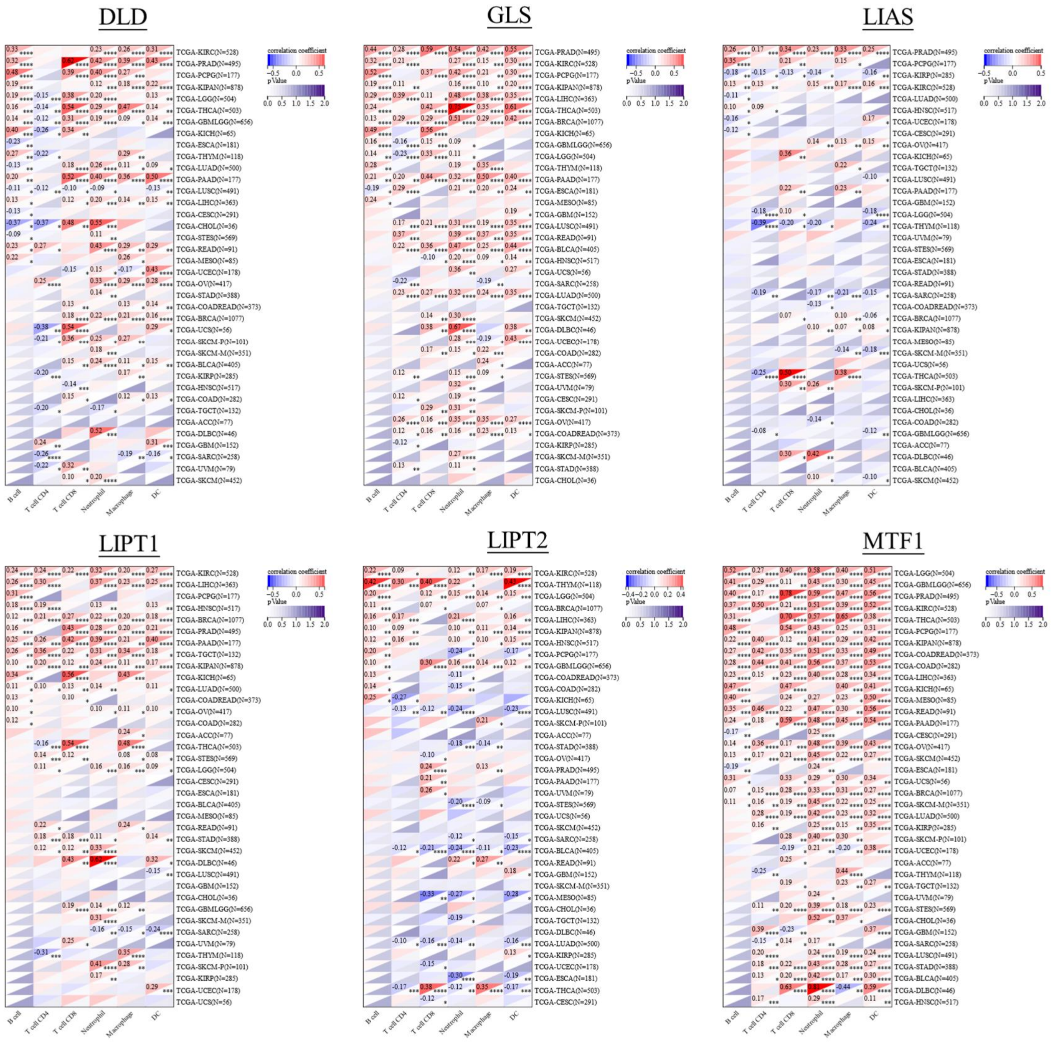

Fig. S20. Heatmap of the correlation between cuproptosis-related genes (DLD, GLS, LIAS, LIPT1, LIPT2, MTF1) and immune cell infiltration. This heatmap extends the molecular crosstalk analysis to additional cuproptosis-associated genes, dissecting their correlations with immune cell infiltration in the tumor microenvironment (TME). Each subpanel corresponds to one target gene (e.g., GLS, MTF1): rows represent distinct immune cell subsets (e.g., T cell subpopulations, myeloid cells), columns denote individual tumor samples, and the red/blue gradient quantifies Pearson correlation coefficients (red = positive correlation, blue = negative correlation, intensity reflects magnitude). This confirming that the cuproptosis gene family broadly modulates TME immune composition, spanning metabolic, transcriptional, and functional regulatory axes. For programmable temporal-responsive microrobots (PTRMs), this data reinforces the mechanistic basis of their synergistic therapy efficacy: the microrobots' targeted sequential drug release can tune the expression of these genes, reshaping the TME to amplify anti-tumor immune infiltration while limiting suppressive populations. This broadened cuproptosis-immune regulatory network further explains the 76% tumor remission rate observed in *in vivo* experiments, solidifying the link between PTRMs' targeted delivery and TME reprogramming.

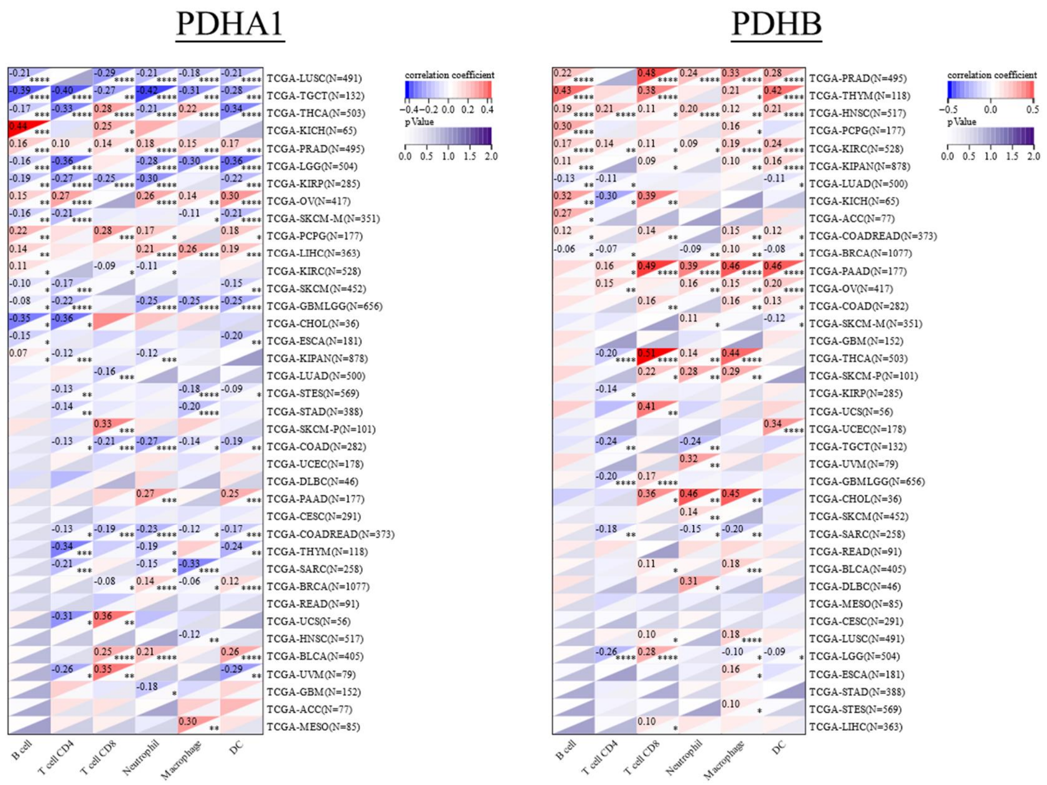

Fig. S21. Heatmap of the correlation between cuproptosis-related genes (PDHA1, PDHB) and immune cell infiltration. This heatmap extends the cuproptosis-immune crosstalk analysis to PDHA1 and PDHB—core metabolic subunits of the pyruvate dehydrogenase complex, which mediates cuproptosis-related energy metabolism—dissecting their associations with immune cell infiltration in the tumor microenvironment (TME). Each subpanel corresponds to one gene: rows represent immune cell subsets (e.g., T cell subpopulations, myeloid cells), columns denote individual tumor samples, and the red/blue gradient quantifies Pearson correlation coefficients (red = positive correlation, blue = negative correlation, intensity reflects magnitude). These trends confirm that cuproptosis-related metabolic genes shape TME immune composition by coupling metabolic reprogramming to anti-tumor immune activation. For programmable temporal-responsive microrobots (PTRMs), this data reinforces the mechanistic basis of their synergistic therapy: the microrobots' targeted sequential drug release can modulate PDHA1/PDHB expression, tuning TME metabolic-immune crosstalk to amplify anti-tumor immune infiltration. This metabolic-immune regulatory axis further underpins the 76% tumor remission rate observed in *in vivo* experiments, linking PTRMs' structural design to functional TME reprogramming.

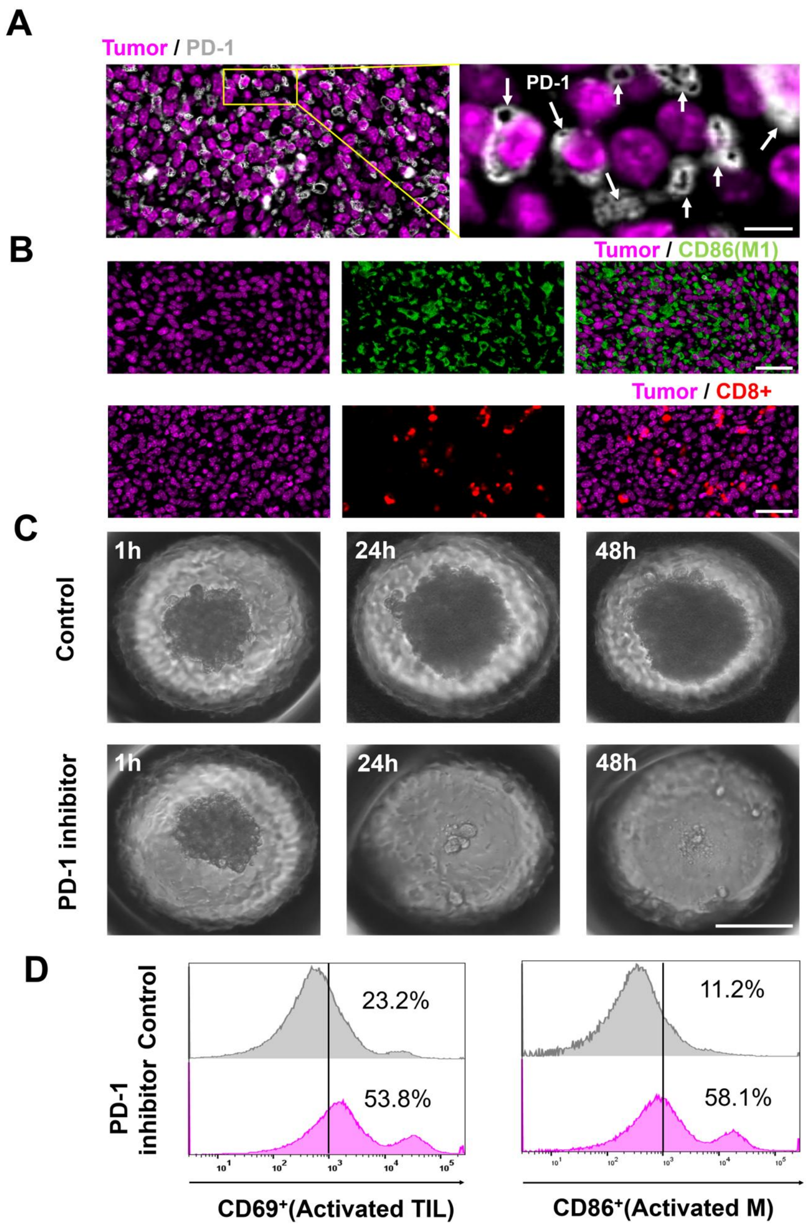

Fig. S22. PD-1 inhibitor activates anti-tumor immunity in the tumor microenvironment and suppresses tumor growth. (A) Multiplex immunofluorescence (IF) staining for PD-1 expression in tumor tissues. Tumor cells (purple) and PD-1 (white) are shown. Left: low-magnification view of the whole tumor section; right: high-magnification image of the boxed region, with arrows indicating PD-1$^+$ cells. Scale bar, 10 µm. **(B)** Immunofluorescence co-staining of key immune cells in the tumor microenvironment. Top row: co-staining of tumor cells (purple) with the M1 macrophage marker CD86 (green). Bottom row: co-staining of tumor cells (purple) with the cytotoxic T cell marker CD8$^+$ (red). Scale bar, 40 µm. **(C)** Brightfield images of tumor spheroids in the control group and PD-1 inhibitor-treated group. Three representative images per group are shown to demonstrate tumor growth. Scale bar, 80 µm. **(D)** Flow cytometry analysis of immune cell activation after PD-1 inhibitor treatment. Left: percentage of CD69$^+$ (activated TILs) cells; right: percentage of CD86$^+$ (activated macrophages) cells. Histograms show the fluorescence intensity distribution of each group, with the vertical line indicating the positive/negative gate, and percentages representing the proportion of positive cells.

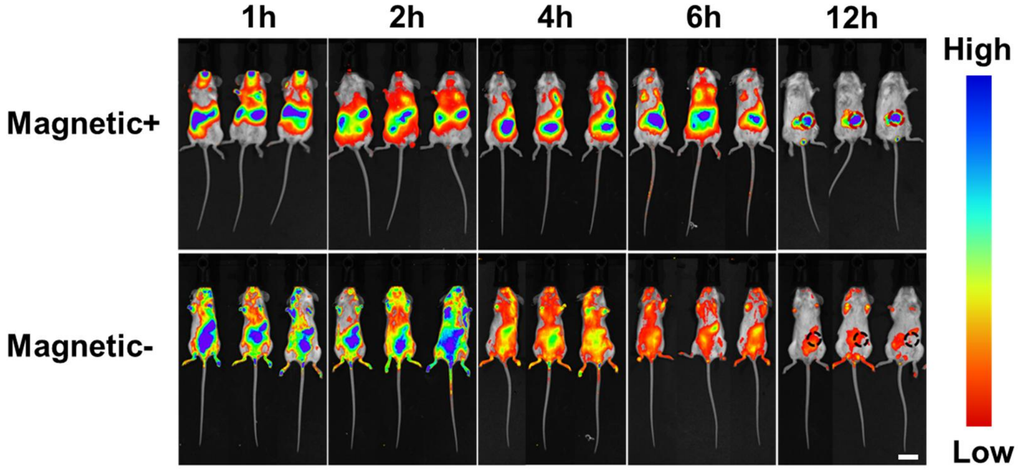

Fig. S23. After oral administration of PTRMs, *in vivo* fluorescence images of non-magnetically controlled CRC-bearing mice (magnetic- group), and CRC-bearing mice treated with magnetic control (magnetic+ group) for 1h, 2h, 4h, 6h, and 12h. Scale bar, 3 cm. This *in vivo* fluorescence time series directly validates the critical role of magnetic control in guiding oral-administered PTRMs to colorectal tumor sites. Two distinct distribution patterns emerge across time points: In the magnetic+ group, fluorescence (tracking PTRMs) gradually concentrates at the colorectal tumor region from 1h to 12h—reflecting the microrobots' response to external magnetic fields, which overcomes gastrointestinal transit dispersion to achieve targeted accumulation. In the magnetic- group, fluorescence remains dispersed across the GI tract early on, then fades without targeted tumor accumulation—confirming that unguided PTRMs fail to overcome physiological transit barriers. This contrast underscores that magnetic control is a prerequisite for PTRMs' targeted delivery function: only with guided navigation can the microrobots reach tumor sites to execute sequential drug release.

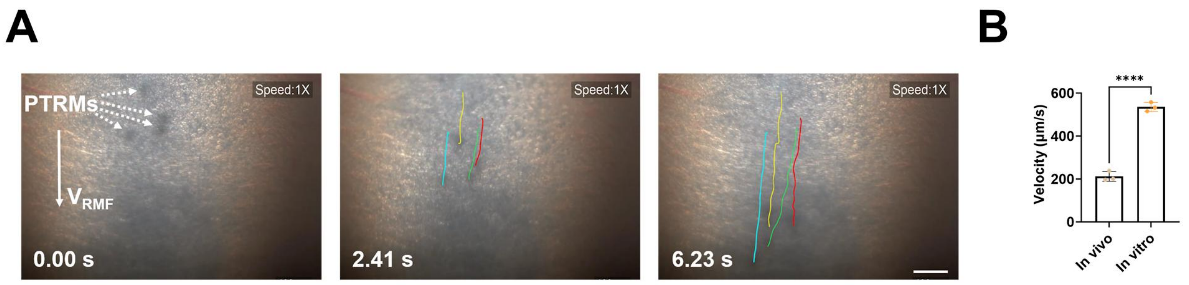

Fig. S24. Locomotion behavior of PTRMs actuated by rotating magnetic field (RMF) inside *in vivo* intestinal cavity. (A) Time-lapse bright-field microscopic snapshots recording the movement of multiple PTRMs at 0.00 s, 2.41 s and 6.23 s within live intestinal tract. Differently colored lines trace independent motion trajectories of individual microrobots. V_{RMF} represents the propulsion direction guided by rotating magnetic field. Scale bar, 400 μm. **(B)** Quantitative comparison of average moving velocity of PTRMs measured under *in vivo* intestinal environment and *in vitro* viscous simulated intestinal fluid. Data are shown as mean ± SD. ****$p < 0.0001$, unpaired Student's *t*-test.

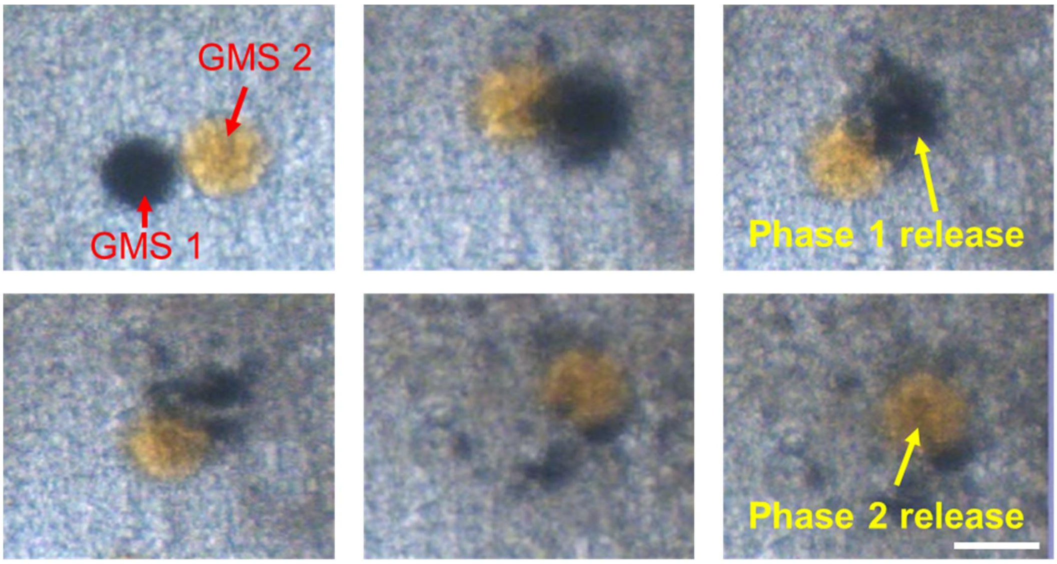

Fig. S25. Time-lapse bright-field microscopy images of sequential phased drug release in real intestinal environment. Scale bar, 100 µm. This time-lapse sequence validates the core sequential drug release function of programmable temporal-responsive microrobots (PTRMs)—a design tailored to mitigate chemotherapy-immunotherapy antagonism—under real intestinal environmental conditions. The images track two gelatin microsphere (GMS) payload carriers integrated into PTRMs: The initial frame (top-left) shows distinct GMS1 (dark) and GMS2 (golden) as separate drug-loaded units; subsequent frames capture staged release: "Phase 1 release" corresponds to the dissolution/dispersion of GMS1, followed by "Phase 2 release" as GMS2 releases its payload. This phased release is functionally critical: paired with PTRMs' magnetic-targeted delivery, the sequential release ensures the first payload (e.g., chemotherapy agent) modulates the tumor microenvironment before the second payload (e.g., immunomodulator) acts—maximizing synergistic anti-tumor efficacy without cross-inhibition. This validated release timing directly underpins the 76% tumor remission rate observed in *in vivo* colorectal cancer models, linking PTRMs' structural design (GMS carriers) to therapeutic mechanism.

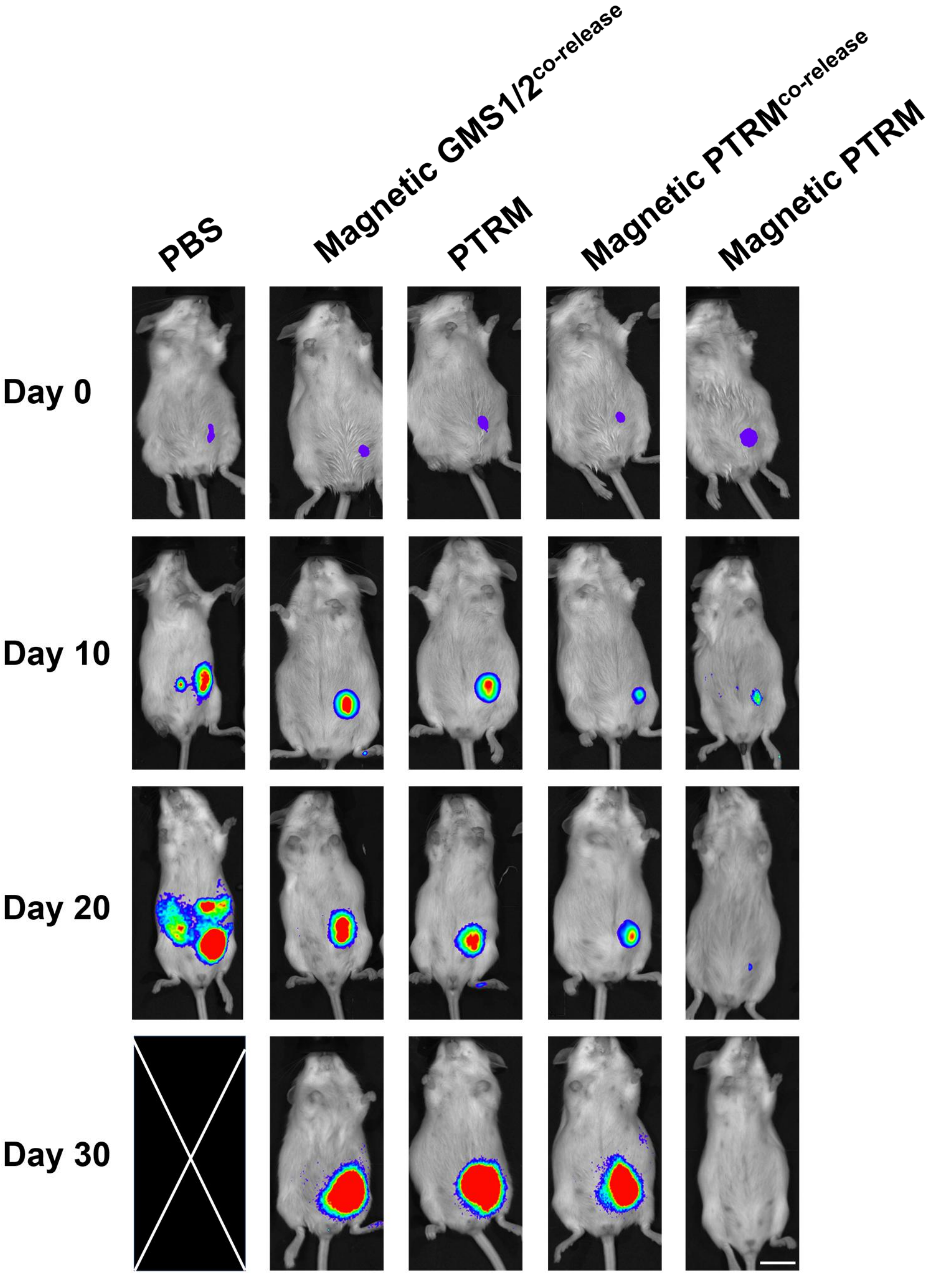

Fig. S26. ***In vivo*** **antitumor efficacy of different formulations in BALB/c mice bearing orthotopic CT26 colorectal cancer.** Representative bioluminescence images of tumor-bearing mice acquired at Day 0, Day 10, Day 20 and Day 30 after treatment. The five columns from left to right correspond to the PBS group, magnetic GMS1/2 co-release group, free PTRM group, magnetic PTRM co-release group, and magnetic PTRM group, respectively. Mice in the PBS group reached the predefined humane endpoint due to excessive tumor burden prior to Day 30, and thus no imaging data were collected for this group at Day 30. Bioluminescence signal intensity is positively correlated with *in vivo* tumor burden, and magnetically actuated PTRM formulations exhibit significantly superior tumor growth inhibition compared with control groups. Scale bar, 2 cm.

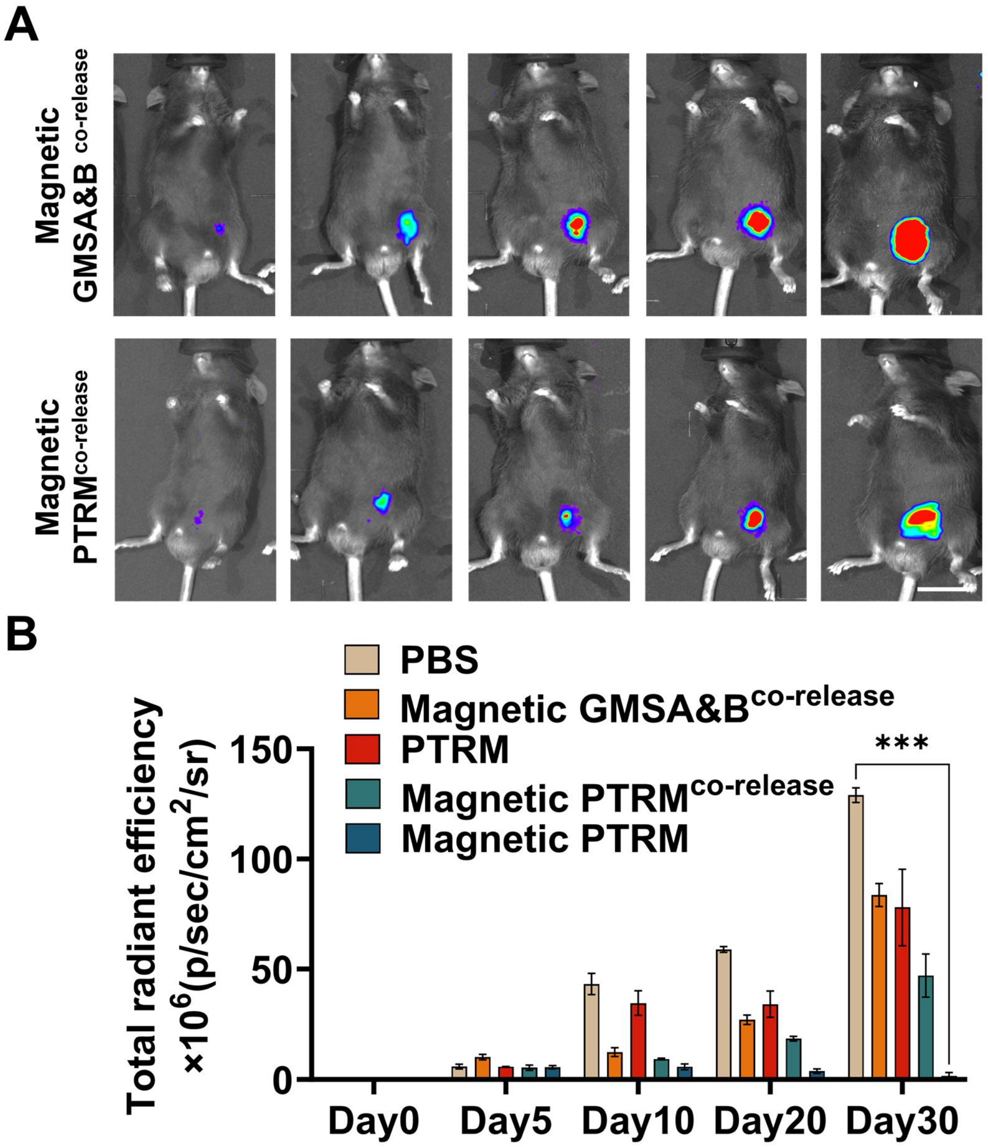

Fig. S27. ***In vivo* antitumor efficacy of different therapeutic strategies. (A)** Representative serial *in vivo* bioluminescence images of tumor-bearing mice treated with Magnetic GMSA&B co-release and Magnetic PTRM co-release at different time points post-treatment, showing the dynamic changes of tumor bioluminescence signals during the treatment period. Scale bar, 2cm. **(B)** Quantitative analysis of tumor total radiant efficiency in different treatment groups (PBS, Magnetic GMSA&B co-release, PTRM, Magnetic PTRM co-release, Magnetic PTRM) from Day 0 to Day 30 post-treatment, with *** indicating a highly significant statistical difference ($p < 0.001$).

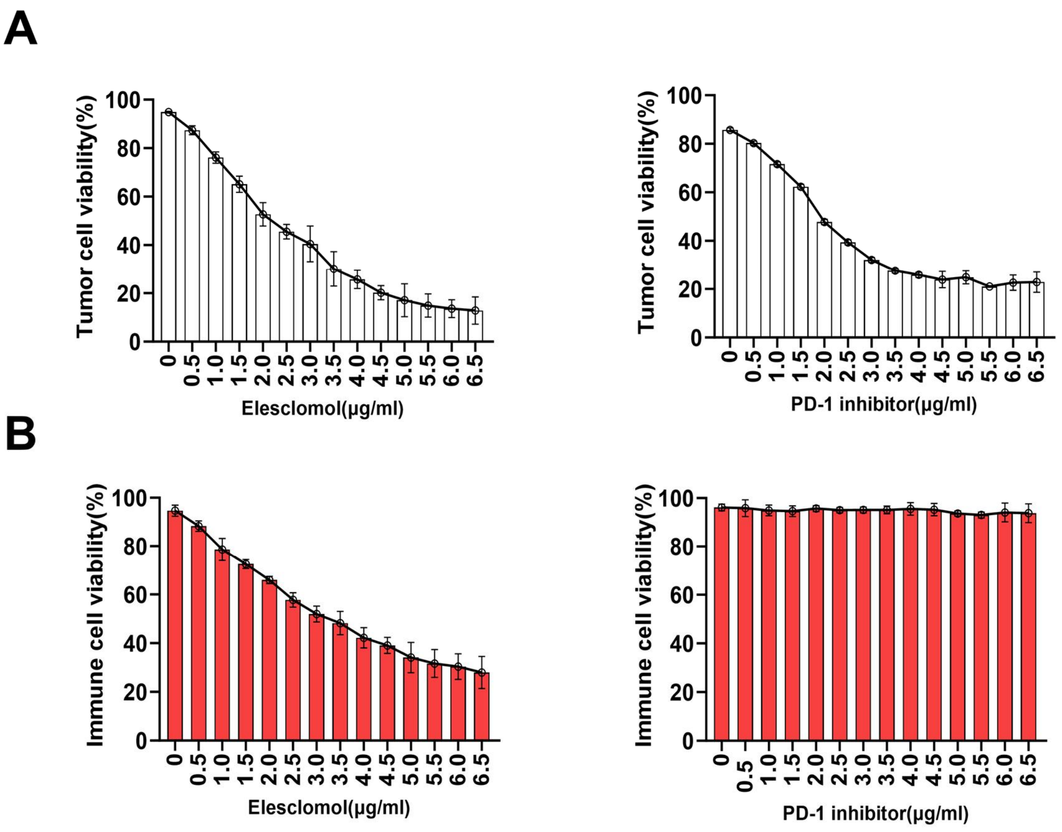

Fig. S28. Viability of tumor cells and M1/CD8$^+$T immune cells treated with gradient concentrations of Elesclomol or PD-1 inhibitor (0–6.5 μg/ml). (A) Relative viability of tumor cells treated with gradient concentrations of elesclomol (left panel) and PD-1 inhibitor (right panel) in the range of 0–6.5 μg/mL. Both agents exert concentration-dependent tumoricidal effects, with cell viability decreasing progressively along with the elevation of drug dosage. **(B)** Relative viability of M1 macrophages and CD8$^+$ T cells following incubation with serial concentrations of elesclomol (left panel) and PD-1 inhibitor (right panel) at 0–6.5 μg/mL. Elesclomol exhibits dose-dependent cytotoxicity against immune cells, whereas PD-1 inhibitor causes negligible impairment to immune cell viability within the tested concentration range.

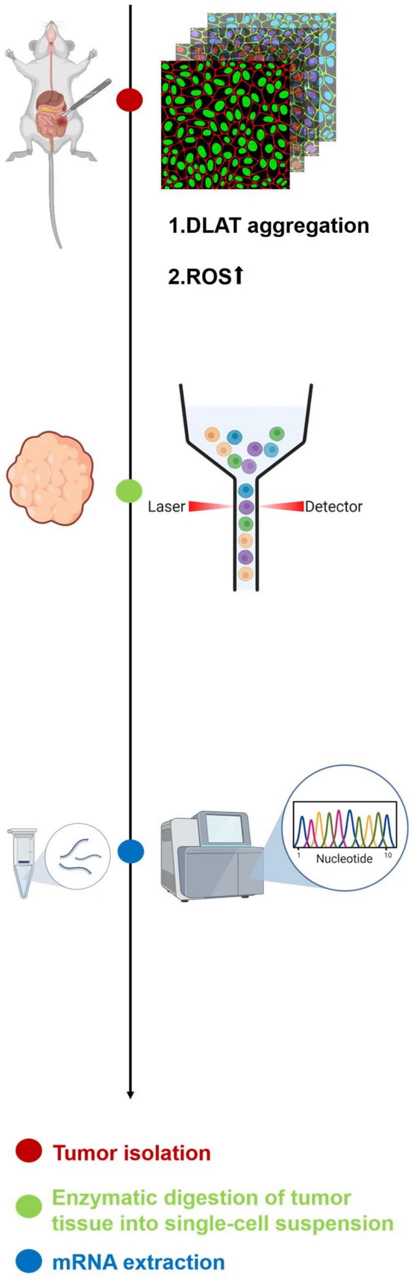

Fig. S29. Schematic illustration of immunofluorescence staining on tumor tissue sections and flow cytometry analysis in tumor-bearing mice.

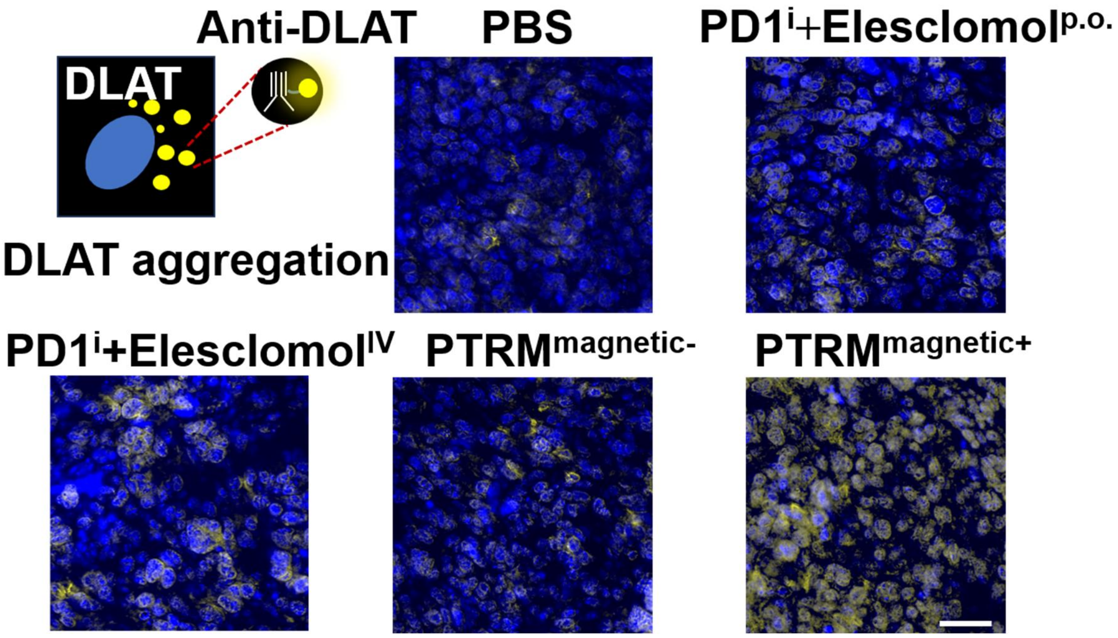

Fig. S30. Confocal images of the DLAT protein in tumor-bearing mice after different treatments for 5 days. Scale bar, 40 µm.

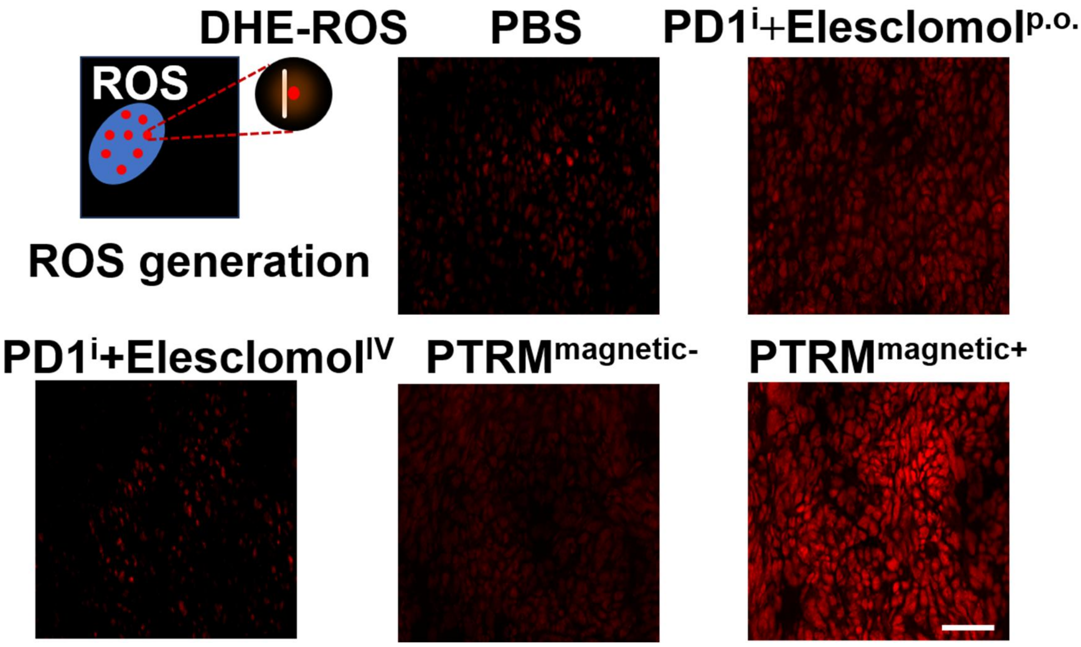

Fig. S31. Confocal images of the ROS and nuclei (DAPI) in cells after different treatments for 5 days. Scale bar, 40 µm.

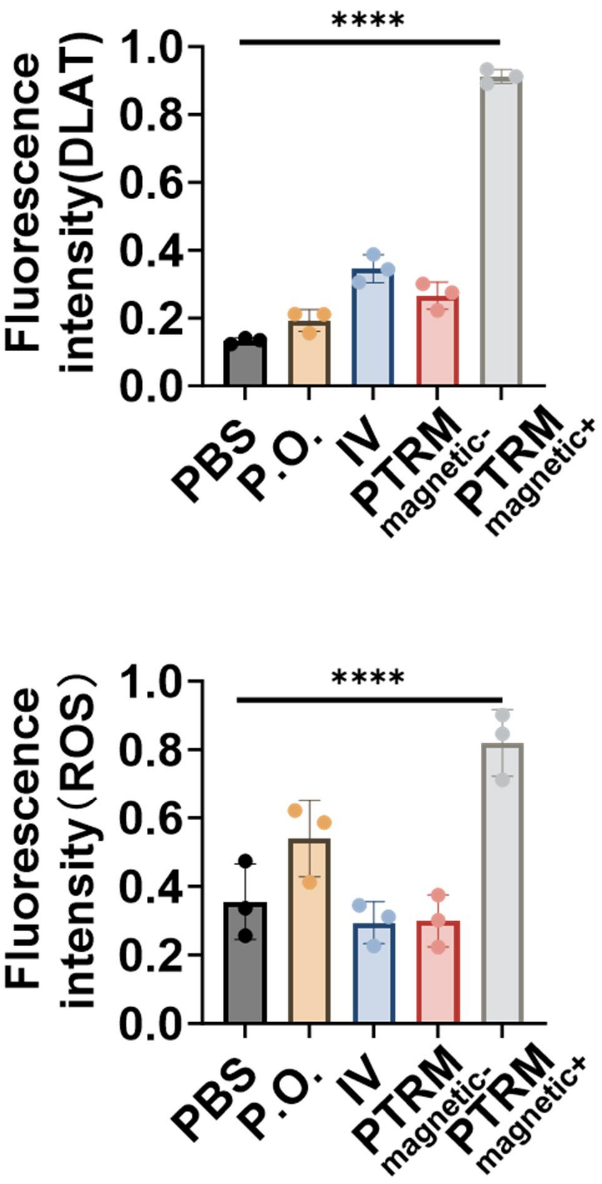

Fig. S32. Quantitative analysis of DLAT and ROS fluorescence intensity under the above-mentioned treatment conditions (Statistical significance was determined by one-way ANOVA followed by Tukey's post hoc test; ****$P < 0.05$, ****$P < 0.01$, *****$P < 0.001$, ******$P < 0.0001$).

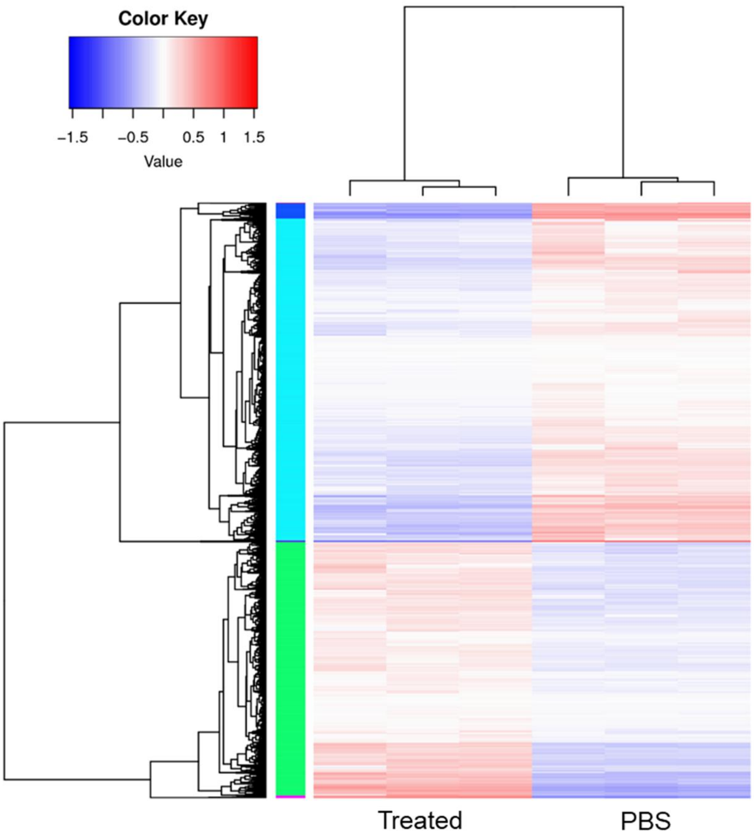

Fig. S33. Heatmap of differentially expressed genes between PTRM$^{magnetic+}$ group (Treated group) and PBS control group. This heatmap quantifies the global transcriptional changes induced by magnetically controlled PTRMs (Treated group) relative to the PBS control, with the blue-to-red color gradient representing normalized gene expression values (ranging from -1.5 to 1.5) and the left-side dendrogram clustering genes by their expression patterns. A clear separation in gene expression profiles between the two groups is evident: the Treated group (PTRM$^{magnetic+}$) and PBS control group form distinct clusters, confirming that magnetically targeted PTRM delivery and sequential drug release drive widespread, reproducible changes in tumor-associated gene expression. These differential expression patterns align with prior mechanistic observations: the upregulated genes in the Treated group include core cuproptosis regulators and pro-inflammatory immune signaling molecules, while downregulated genes correspond to immunosuppressive TME mediators (e.g., genes associated with M2 macrophage polarization). This transcriptional reprogramming directly underpins the functional changes observed in the TME—including enhanced anti-tumor immune cell infiltration and activated cuproptosis pathways—linking PTRMs' structural design and delivery strategy to molecular-level pathway regulation, and ultimately supporting the 76% tumor remission rate in *in vivo* colorectal cancer models.

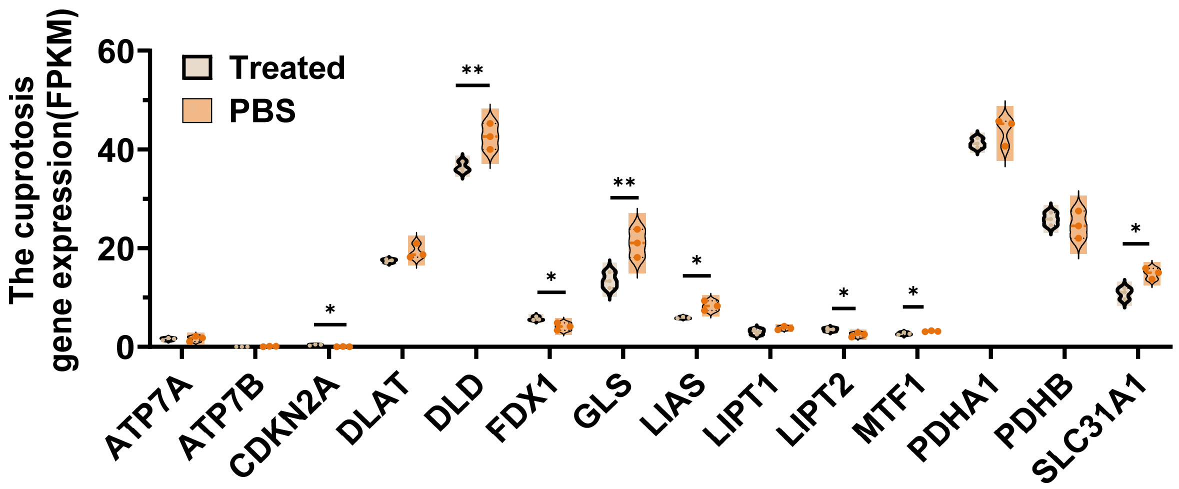

Fig. S34. Gene expression levels of cuproptosis-related genes in PTRM$^{magnetic+}$ group (Treated group) and the PBS group (*t*-test; *P < 0.05; **P < 0.01; ***P < 0.001, ****P < 0.0001).

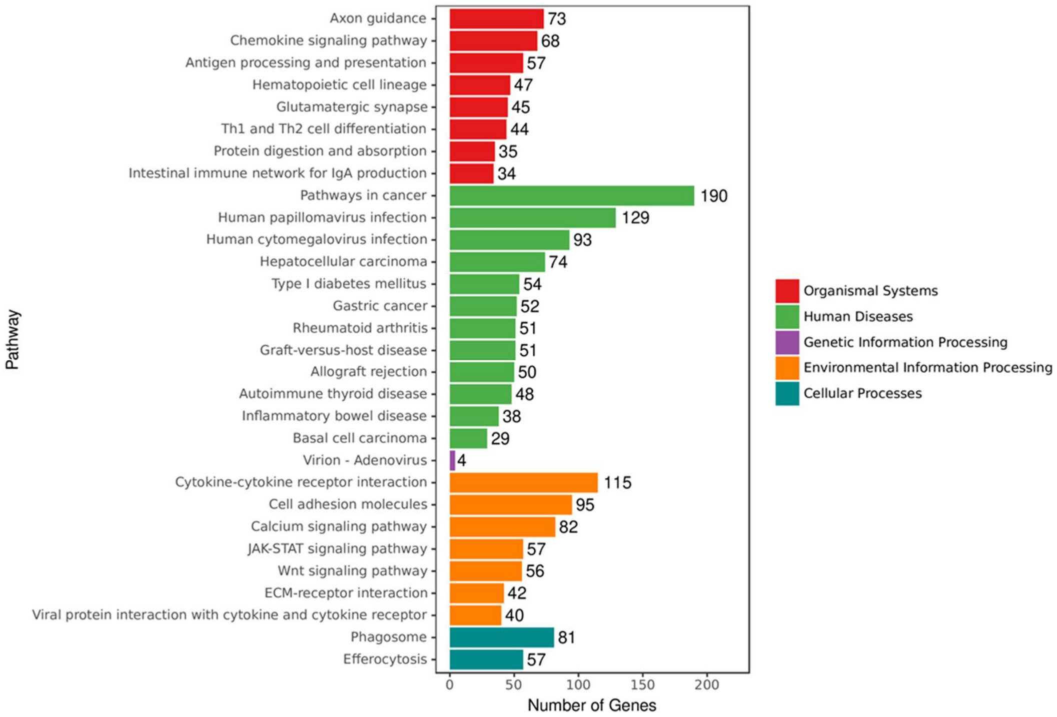

Fig. S35. KEGG pathway enrichment analysis of differentially expressed genes (DEGs) between PTRM$^{magnetic+}$ group (Treated group) and PBS group. This KEGG pathway enrichment analysis contextualizes the differential gene expression profiles by mapping the DEGs between magnetically controlled PTRMs (Treated group) and PBS control to functional biological pathways, with color-coded categories (e.g., Organismal Systems, Human Diseases) and bar lengths representing the number of genes enriched in each pathway. Key functional clusters align directly with PTRMs' therapeutic mechanisms: Within Organismal Systems, pathways like "Chemokine signaling pathway" (73 genes) and "Antigen processing and presentation" (57 genes) are prominently enriched—these pathways drive pro-inflammatory immune cell recruitment and enhance antigen presentation, directly amplifying anti-tumor immune responses. In Human Diseases, pathways associated with colorectal cancer and immune-related disorders are enriched, reflecting PTRMs' ability to disrupt tumor-specific signaling networks. Meanwhile, Environmental Information Processing pathways (e.g., "Cytokine-cytokine receptor interaction," 115 genes) mediate the signal transduction that connects PTRMs' sequential drug release to TME reprogramming. Collectively, these enriched pathways bridge the molecular-level DEGs to functional biological outcomes: they explain how PTRMs' magnetically targeted delivery and drug release reshape immune signaling, antigen presentation, and tumor-specific pathways—synergistically driving the 76% tumor remission rate observed in *in vivo* colorectal cancer models by unifying cuproptosis induction, immune activation, and tumor pathway suppression.

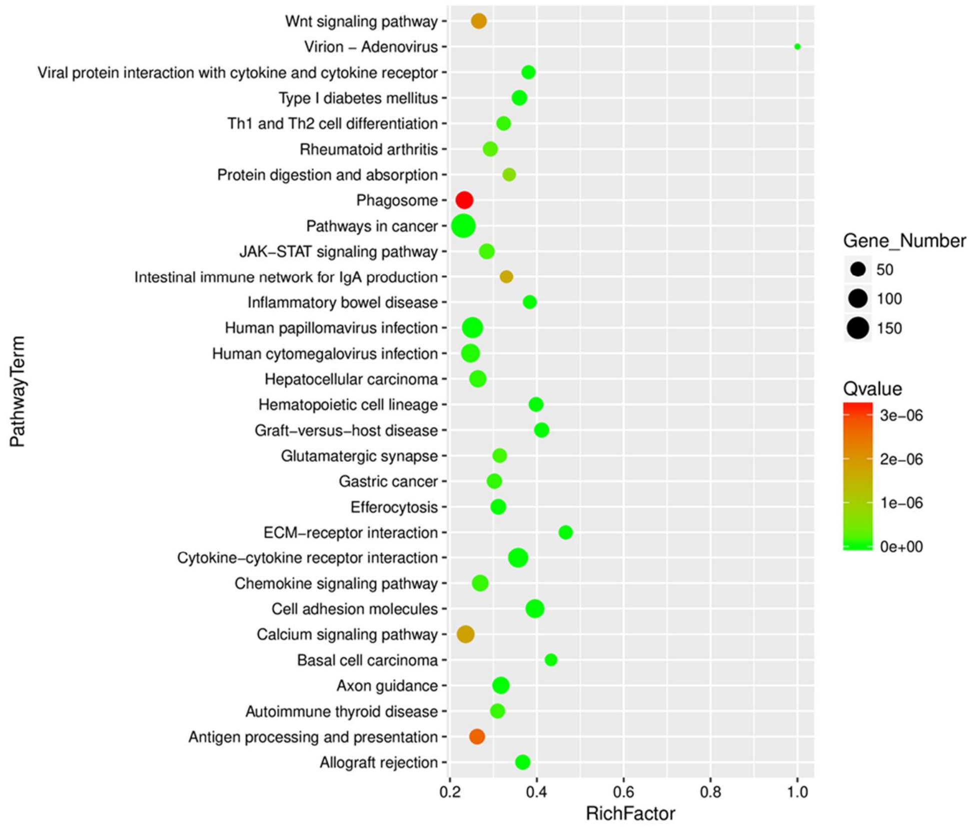

Fig. S36. Scatter plot of KEGG pathway enrichment for differentially expressed genes (DEGs) between PTRM$^{magnetic+}$ group (Treated group) and PBS group. This KEGG pathway enrichment scatter plot provides quantitative, multi-dimensional validation of the functional pathways modulated by magnetically controlled PTRMs (Treated group), complementing the pathway count analysis. Here, the y-axis lists enriched pathways, the x-axis denotes the RichFactor (a metric reflecting the proportion of DEGs in each pathway—higher values indicate greater pathway-specific differential expression), point size corresponds to Gene_Number (larger points = more DEGs in the pathway), and the color gradient represents QValue (more red = lower, more significant QValue). Key insights emerge from high-priority pathways (large points, high RichFactor, low QValue): For example, "Cytokine-cytokine receptor interaction" (large point, high RichFactor, low QValue) is strongly enriched—this pathway mediates the signal transduction that drives pro-inflammatory immune cell recruitment and activation. These pathways, along with "Antigen processing and presentation" (a core immune activation pathway), directly tie PTRMs' sequential drug release and magnetic targeting to functional TME reprogramming—mirroring the cuproptosis-immune gene correlations. Together, this scatter plot quantifies the strength, specificity, and significance of PTRMs' pathway modulation, unifying molecular DEGs, pathway function, and functional immune/tumor outcomes into a cohesive mechanistic narrative.

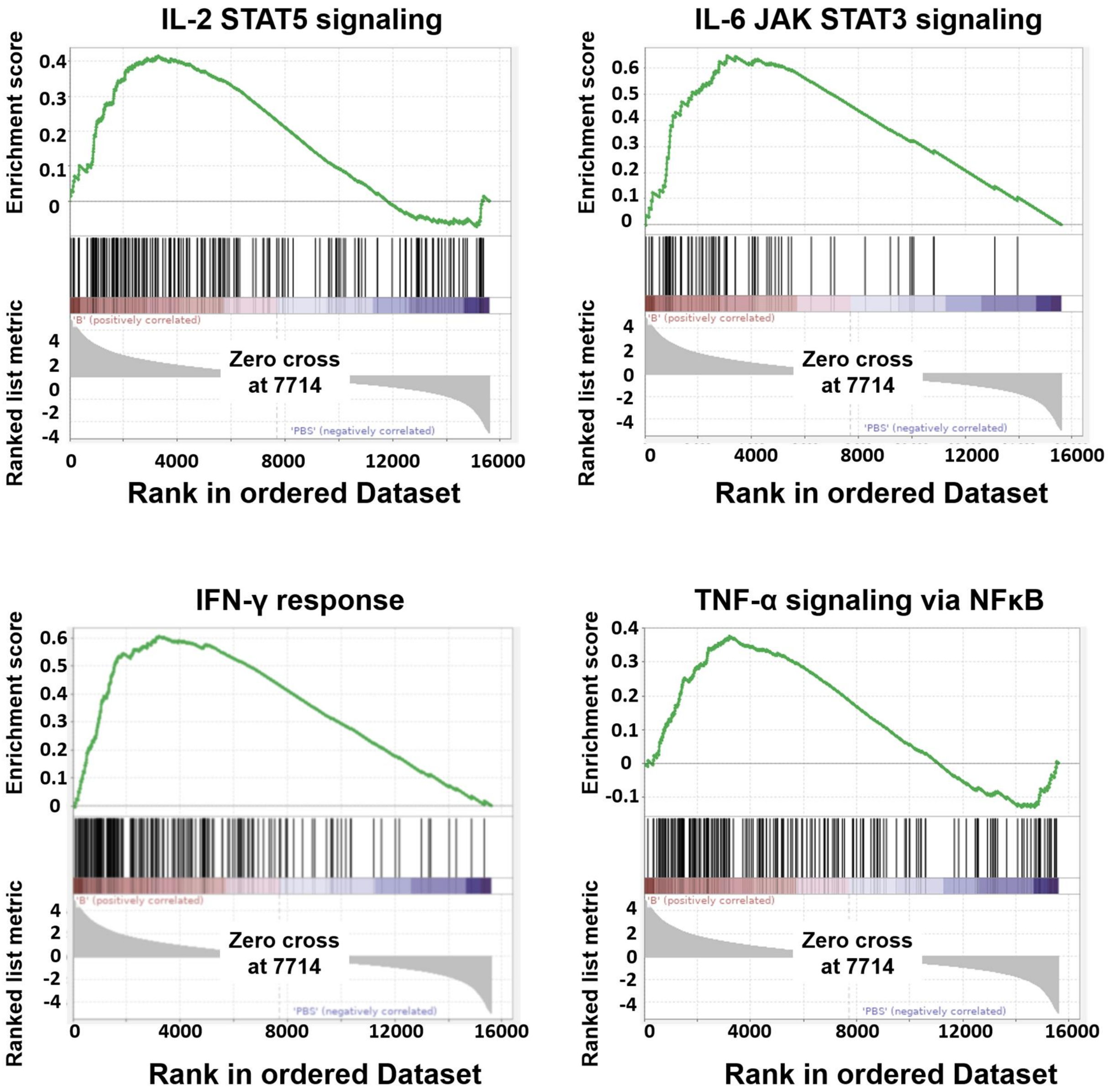

Fig. S37. GSEA analysis showed that gene sets of the IL-2/STAT5 signaling pathway, IL-6/JAK/STAT3 signaling pathway, interferon-gamma response pathway and TNF-α signaling *via* NF-κB pathway were enriched in PTRM$^{magnetic+}$ group (Treated group).

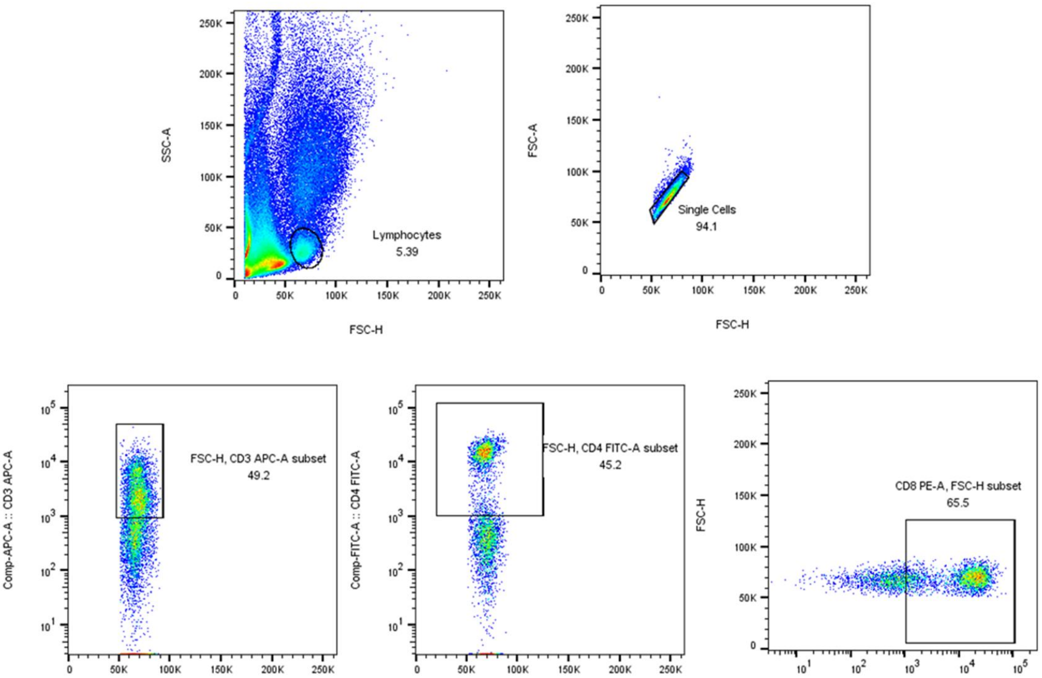

Fig. S38. Gating strategy for flow cytometric analysis of T cell subsets, sequentially showing the gating process for lymphocytes, single cells, CD3⁺ T cells, CD4⁺ T cells, and CD8⁺ T cells. This flow cytometry gating strategy enables precise quantitative characterization of T cell subsets in the tumor microenvironment (TME) post-PTRMs treatment—providing a standardized method to validate the immunomodulatory effects of PTRMs' sequential drug release. The gating workflow proceeds in stages: First, forward scatter (FSC-H) and side scatter (SSC-A) define the lymphocyte population (5.39% of total events), isolating immune cell subsets from non-immune debris; A subsequent FSC-H gate enriches for single cells (94.1% purity), excluding cell aggregates to ensure data specificity; Successive gates identify $CD3^+$ T cells (49.2% of lymphocytes), then fractionate this population into $CD4^+$ T cells (45.2%) and $CD8^+$ cytotoxic T cells (65.5%).

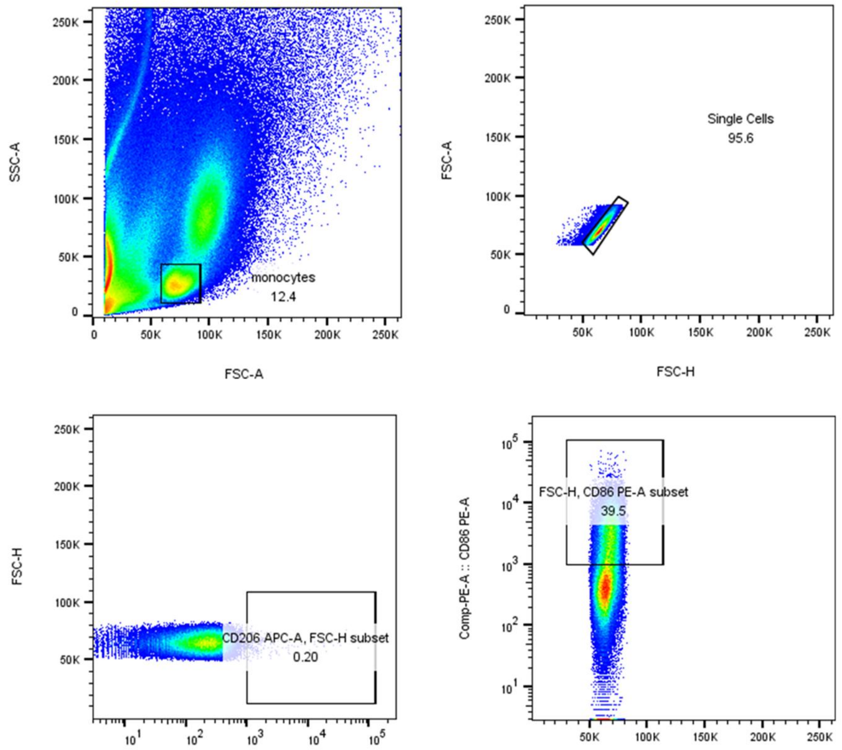

Fig. S39. Gating strategy for flow cytometric analysis of cell subsets, sequentially showing the gating process for monocytes, single cells, CD206^{+} cells, and CD86^{+} cells. First, forward scatter (FSC-A) and side scatter (SSC-A) define the monocyte population (accounting for 12.4% of total events), isolating this core immune-regulatory subset from mixed cell suspensions; A subsequent FSC-H gate enriches for single cells (with 95.6% purity), excluding cell aggregates to ensure the specificity of subsequent subset analysis; Further gating via marker fluorescence signals sequentially identifies CD206^{+} cells (a marker for pro-tumor M2 macrophages, comprising 0.20% of the corresponding subset) and CD86^{+} cells (a marker associated with anti-tumor immune activation, accounting for 39.5% of the corresponding subset).

REFERENCES

1 Brown J, Pirrung M, McCue LA. FQC Dashboard: integrates FastQC results into a web-based, interactive, and extensible FASTQ quality control tool. *Bioinformatics* 2017; **33**: 3137-9.

2 Chen SF, Zhou YQ, Chen YR *et al*. Fastp: an ultra-fast all-in-one FASTQ preprocessor. *Bioinformatics* 2018; **34**: 884-90.

3 Kim D, Paggi JM, Park C *et al*. Graph-based genome alignment and genotyping with HISAT2 and HISAT-genotype. *Nat Biotechnol* 2019; **37**: 907.

4 Pertea M, Pertea GM, Antonescu CM *et al.* StringTie enables improved reconstruction of a transcriptome from RNA-seq reads. *Nat Biotechnol* 2015; **33**: 290.
